# Carbon storage in China’s terrestrial ecosystems: A synthesis

**DOI:** 10.1038/s41598-018-20764-9

**Published:** 2018-02-12

**Authors:** Li Xu, Guirui Yu, Nianpeng He, Qiufeng Wang, Yang Gao, Ding Wen, Shenggong Li, Shuli Niu, Jianping Ge

**Affiliations:** 1 Key Laboratory of Ecosystem Network Observation and Modeling, Institute of Geographic Sciences and Natural Resources Research, Chinese Academy of Sciences, Beijing, 100101 China; 2 College of Resources and Environment, University of Chinese Academy of Sciences, Beijing, 100049 China; 3 College of Life Sciences, Beijing Normal University, Beijing, 100875 China

## Abstract

It is important to accurately estimate terrestrial ecosystem carbon (C) storage. However, the spatial patterns of C storage and the driving factors remain unclear, owing to lack of data. Here, we collected data from literature published between 2004 and 2014 on C storage in China’s terrestrial ecosystems, to explore variation in C storage across different ecosystems and evaluate factors that influence them. We estimated that total C storage was 99.15 ± 8.71 PgC, with 14.60 ± 3.24 PgC in vegetation C (Veg-C) and 84.55 ± 8.09 PgC in soil organic C (SOC) storage. Furthermore, C storage in forest, grassland, wetland, shrub, and cropland ecosystems (excluding vegetation) was 34.08 ± 5.43, 25.69 ± 4.71, 3.62 ± 0.80, 7.42 ± 1.92, and 15.17 ± 2.20 PgC, respectively. In addition to soil nutrients and texture, climate was the main factor regulating the spatial patterns of C storage. Climate influenced the spatial patterns of Veg-C and SOC density via different approaches, Veg-C was mainly positively influenced by mean annual precipitation (MAP), whereas SOC was negatively dependent on mean annual temperature (MAT). This systematic estimate of C storage in China provides new insights about how climate constrains C sequestration, demonstrating the contrasting effects of MAP and MAT on Veg-C and SOC; thus, these parameters should be incorporated into future land management and C sequestration strategies.

## Introduction

Terrestrial ecosystems are the main component of carbon (C) pools in the Earth’s system, and contribute considerably to the global C balance^1–3^. Furthermore, terrestrial ecosystems are a major C sink, sequestering approximately 28% of CO_2_ emissions originating from anthropogenic activity^4,5^. Enhancing C storage in terrestrial ecosystems is widely considered as an effective and environmentally friendly measure to sequester anthropogenic CO_2_ emissions^6,7^. Therefore, it is important for policy makers to obtain accurate estimates of C storage and to understand what factors influence its spatial distribution across ecosystems.

China covers 6.4% of the global terrestrial area, and is crucial in determining the global C balance in terms of both C emissions and C uptake^3,8,9^. Some studies have estimated the C storage capacity of China’s terrestrial ecosystem by using inventory data or remote sensing data^10–12^. However, most of these studies focused on assessing vegetation C (Veg-C) or soil organic C (SOC) separately, or only focused on one specific type of ecosystem (e.g., forest, grassland)^13–17^.

Some studies have also explored C storage in China’s terrestrial ecosystems using alternative approaches, such as modeling or statistical methods^6,11–43^ (Table 1). Yet, uncertainty remains high among the various studies, especially with respect to estimating SOC storage in China’s terrestrial ecosystems, with values ranging from 50.0 to 185.7 Pg C (Table 1). This large uncertainty is mainly attributed to variation in the collection periods, data validity, and estimation methods^19^. Thus, it is essential to obtain robust estimates of C storage at large scales using comprehensive data and optimized methods. In fact, field investigations represent a source of credible and reliable data, and might reliably reflect the status of Veg-C and SOC, with the required monitoring instruments and operating methods of field investigations being simple^3,13,35,44–46^. However, few studies have used field investigation data to estimate C storage in China’s terrestrial ecosystems, because a synthetic field investigation of C storage in vegetation and soil at a national scale is time-consuming, laborious, and difficult to implement, especially in remote areas^44^.

**Table 1:** Carbon density and storage reported from different ecosystems in China and China’s terrestrial ecosystems.

**Ecosystems**	**Period**	Approach	Data source	Area	Vegetation	C storage (Pg C)	Soil^†^	C storage (Pg C)	Ecosystem	References
				(×10^4^ km^2^)	C density (kg C m^−2^)		C density (kg C m^−2^)		C storage (Pg C)	
Forest	1999–2003	Statistics	National forest inventory data	142.80	4.10	5.85				^6^
	1994–1998	Statistics	National forest inventory data	105.82	4.49	4.75				^13^
	2004–2008	Statistics	National forest inventory data	182.48	4.28	7.81				^16^
	1989–1993	Statistics	National forest inventory data and investigation data	127.06	7.17	9.11				^18^
	1989–1993	Statistics	National forest inventory data	108.64	3.87	4.20				^20^
	1989–1993	Statistics	Published data and national forest investigation data	108.62	5.71	6.20	19.36	21.02	27.22	^21^
	1989–1993	Statistics	National forest inventory data	108.60	4.00	4.34				^22^
	1979–1985	Statistics	China’s second national soil survey	150.00			11.59	17.39		^23^
	1989–1993	Statistics	National forest inventory data	91.43	4.13	3.78				^24^
	1982–1999	Modeling	National forest inventory data and NDVI data	127.89	4.53	5.79				^25^
	1979–1985	Statistics	China’s second national soil survey	249.32			13.73	34.23		^26^
	1999–2003	Statistics	National forest inventory data	142.79	3.86	5.51				^27^
	1979–2004	Statistics	China’s second national soil survey and investigation data	197.13			10.50	20.7		^28^
	1999–2003	Statistics	National forest inventory data			5.16				^29^
	2004–2008	Statistics	National forest inventory data	186.21	4.08	7.59				^30^
	2004–2008	Modeling (HASM)	National forest inventory data	195.45	4.73	9.24				^31^
	1979–1985	Statistics	China’s second national soil survey	179.48			10.63	19.08		^32^
	**2004–2014**	**Statistics**	**Published literature and investigation data**	**195.89**	**5.86 ± 1.62**	**11.49 ± 3.18**	**11.53 ± 2.24**	**22.59 ± 4.40**	**34.08 ± 5.43**	**This study**
Grassland	1981–1998	Modeling (CEVSA)	FAO database and NDVI data	166.96	0.34	0.56	9.99	16.69	17.25	^12^
	1981–1988	Statistics	National grassland resource survey data	298.97	1.15	3.06	13.16	41.03	44.09	^14^
	1979–1985	Statistics	China’s second national soil survey	223.00			8.83	19.68		^23^
	1979–1985	Statistics	China’s second national soil survey	278.51			13.54	37.71		^26^
	1979–1985	Statistics	China’s second national soil survey and investigation data	268.35			9.17	24.60		^28^
	1979–1985	Statistics	China’s second national soil survey	296.70			9.29	27.58		^32^
	1982–1999	Statistics	National grassland resource survey data and NDVI data	331.41	0.31	1.04				^33^
	1981–1988	Statistics	National grassland resource survey data and NDVI data	334.10	0.32	1.05				^34^
	2003–2004	Statistics	Investigation data	331.00	1.00	3.32				^35^
	1981–1988	Statistics	Published literature	331.41	0.30	0.99	8.48	28.11	29.1	^36^
	2003–2014	Statistics	Published literature	355.05	0.50	1.61	7.96	29.37	30.98	^37^
	**2004–2014**	**Statistics**	**Published literature and investigation data**	**280.44**	**0.69 ± 0.20**	**1.94 ± 0.55**	**8.47 ± 1.67**	**23.75 ± 4.68**	**25.69 ± 4.71**	**This study**
Cropland	1981–1998	Modeling (CEVSA)	FAO database and NDVI data	172.89	0.57	0.98	10.84	18.73	19.71	^12^
	1979–1985	Statistics	China’s second national soil survey	182.00			8.07	14.67		^23^
	1979–1985	Statistics	China’s second national soil survey and investigation data	167.03			7.57	12.65		^28^
	1979–1985	Statistics	China’s second national soil survey	178.51			8.43	15.04		^32^
	**2004–2014**	**Statistics**	**Published literature**	**171.53**			**8.85 ± 1.17**	**15.17 ± 2.00**		**This study**
Wetland		Statistics	Published literature	22.50		0.13–0.50		5.04–6.19		^17^
	1979–1985	Statistics	China’s second national soil survey	11.89			14.76	1.75		^32^
	**2004–2014**	**Statistics**	**Published literature**	**14.46**	**1.40 ± 0.43**	**0.20 ± 0.06**	**23.60 ± 5.51**	**3.41 ± 0.80**	**3.62 ± 0.80**	**This study**
Shrub	1981–1998	Modeling (CEVSA)	FAO database and NDVI data	216.53	1.47	2.32	9.17	11.78	14.10	^12^
		Statistics	Published literature	154.62	10.88	1.68				^15^
	1979–1985	Statistics	China’s second national soil survey	188.00			7.25	13.62		^23^
	**2004–2014**	**Statistics**	**Published soil organic carbon data**	**77.69**	**0.56 ± 0.13**	**0.44 ± 0.10**	**8.98 ± 2.47**	**6.98 ± 1.92**	**7.42 ± 1.92**	**This study**
Terrestrial		Modeling(OBM)	WOSCN database	968	5.98	57.90	10.33	100.00	157.90	^11^
	1981–1998	Modeling (CEVSA)	FAO database and NDVI data	901.14	1.47	13.33	9.17	82.65	95.98	^12^
		Modeling(BIOME3)	WOSCN database	959.63	6.00	57.57	12.48	119.80	177.37	^18^
			Published literature			35.23		119.76	154.99	^19^
	1979–1985	Statistics	China’s second national soil survey	870.94			10.29	89.61		^26^
	1979–2004	Statistics	China’s second national soil survey and investigation data	880.37			7.80	69.10		^28^
	1979–1985	Statistics	China’s second national soil survey	938.79			9.31	87.36		^32^
	1958–1960	Statistics	China’s first national soil survey and forest inventory data	944.86	0.65	6.10	20.3	185.70	191.70	^38^
	1979–1985	Statistics	China’s second national soil survey	915			4.86	50.00		^39^
	1979–1985	Statistics	China’s second national soil survey	877.63			10.53	92.42		^40^
	1979–1985	Statistics	China’s second national soil survey	881.81			8.01	70.31		^41^
	1979–1985	Statistics	China’s second national soil survey	928.10			9.60	89.14		^42^
	1979–1985	Statistics	China’s second national soil survey	928.10			9.46	87.78		^43^
	**2004–2014**	**Statistics**	**Published soil organic carbon data**	**925.64**	**1.58 ± 0.35**	**14.60 ± 3.24**	**9.13 ± 0.87**	**84.55 ± 8.09**	**99.15 ± 8.71**	**This study**

^†^Soil depths at which soil organic carbon (SOC) density and storage were estimated was approximately 100 cm in this table.

Understanding the spatial patterns and key influencing factors of C storage at large scales could help us to adopt effective sequestration strategies. Theoretically, several factors, such as site conditions (climate), vegetation type, soil properties (clay content, soil moisture, pH, nutrient status), and land use, could influence the spatial patterns of C storage in vegetation and soil via different processes or mechanisms^25,47–50^. Among these factors, climate (principally mean annual temperature (MAT) and mean annual precipitation (MAP)) and vegetation type are generally assumed to be the major factors influencing the spatial distribution of Veg-C and SOC^47,49,51,52^. In natural ecosystems, vegetation C (Veg-C) storage is determined by the balance between C absorption during photosynthesis and the release of C by respiration and dead biomass, with MAT and MAP influencing the net primary productivity of vegetation by regulating the supply of energy and water, which, in turn, influence Veg-C^53^. Soil C storage is determined by the balance between C input by litterfall and rhizodeposition, in addition to the output of C during decomposition^47,54,55^; however, changing temperature might affect the decomposition of soil organic matter (SOM)^56^. Therefore, different factors (MAP *vs*. MAT) are expected to influence the patterns of Veg-C and SOC via different approaches at large scales.

In this study, we collected field-measured C storage data in China’s terrestrial ecosystems from literature published between 2004 and 2014. These data encompassed the main ecosystems in China (forest, grassland, cropland, wetland, and shrub ecosystems), and included different components (above-ground biomass, below-ground biomass, and soil C content at depths of 0–20 cm and 0–100 cm). We estimated the Veg-C and SOC density and storage of different ecosystems, and then summed them to evaluate the C storage in China, based on the statistical method of ecosystem type, which has been widely used to evaluate C storage at a regional and national scale^3,12,50^. To allow our results to be compared with most previous studies, we estimated SOC storage in the 0–20 cm and 0–100 cm soil layers. The main objectives of this study were to: (1) generate a comprehensive C density dataset from which to estimate C storage in China’s terrestrial ecosystems; (2) explore the spatial patterns of Veg-C and SOC density and the main factors influencing these patterns; and (3) test the assumption that climate influences the patterns of Veg-C and SOC density through different approaches (MAT *vs*. MAP).

## Results

### C storage in China’s terrestrial ecosystems

**Figure 1:**
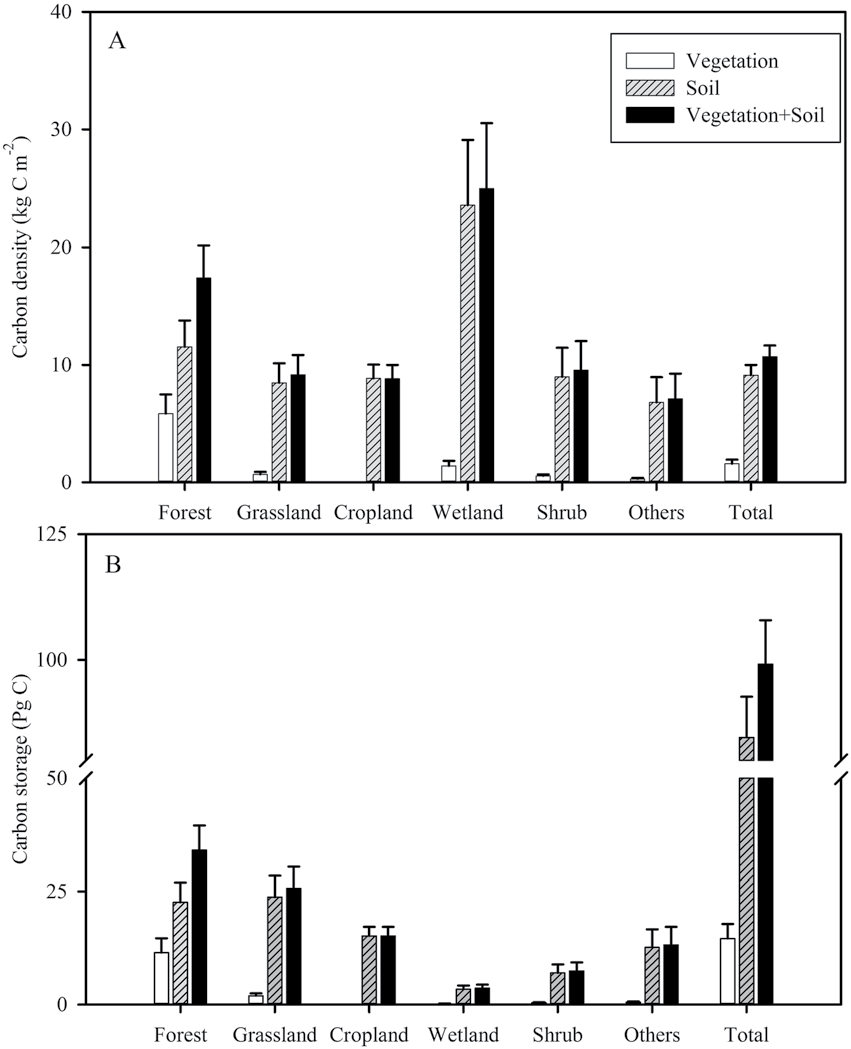
Carbon density (kg C m^−2^) and storage (Pg C) in the different ecosystems of China. The total soil depth used to estimate soil organic carbon (SOC) density and storage was 100 cm in these figures.

Veg-C and SOC (0–100 cm soil layer) storage were estimated as 14.60 ± 3.24 and 84.55 ± 8.09 Pg C, respectively, with a sum of 99.15 ± 8.71 Pg C storage in China’s terrestrial ecosystems (Fig. 1; Supplementary Table S1). The average density of Veg-C and SOC (0–100 cm) was approximately 1.58 ± 0.35 and 9.13 ± 0.87 kg C m^−2^, respectively. AGBC and BGBC storage was 10.01 ± 3.11 and 4.59 ± 0.90 Pg C, respectively. SOC storage in the topsoil (0–20 cm) was estimated as 34.32 ± 3.37 Pg C, and represented 40.59% of SOC storage in the 0–100 cm soil layer (Supplementary Table S1).

C storage in forest, grassland, and shrub ecosystems was 34.08 ± 5.43, 25.69 ± 4.71, and 7.42 ± 1.92 Pg C, with an average density of 17.40 ± 2.77, 9.16 ± 1.68, and 9.55 ± 2.48 kg C m^−2^, respectively (Fig. 1; Supplementary Table S1). For croplands, SOC storage was 15.17 ± 2.00 Pg C. Despite wetlands having high C density (25.69 ± 4.71 kg C m^−2^), they had low C storage (3.62 ± 0.80 Pg C), due to their smaller area (14.46 × 10^4^ km^2^).

### Spatial distribution of C density in China’s terrestrial ecosystems

The spatial distribution of Veg-C differed from that of SOC density. Specifically, Veg-C density declined with increasing latitude, and high Veg-C density and storage was detected in the southeastern regions of China. SOC density increased with increasing latitude, with high SOC density and storage being observed in the northeastern and southeastern regions of China (Supplementary Fig. S2; Table 2). Veg-C density ranged from 0.35 to 4.72 kg C m^−2^ among the 18 ecological regions (Table 2). Veg-C density was generally higher in cold humid regions (R1), temperate humid regions (R2), south subtropical humid regions (R17), and tropical humid regions (R18). Unlike other regions, temperate arid regions (R5) and warm temperate arid regions (R6), which were located in the northwestern region of China, had the lowest Veg-C density (Supplementary Fig. S2; Table 2; Supplementary Table S2). The distribution of SOC density was similar in the 0–20 cm and 0–100 cm soil layers. Cold humid regions (R1) had the highest SOC density, with average densities of 8.88 ± 2.50 and 17.76 ± 7.17 kg C m^−2^ in the 0–20 cm and 0–100 cm soil layer, respectively. In comparison, the lowest SOC density occurred in warm temperate arid regions (R6) (2.12 ± 1.10 kg C m^−2^) for the 0–20 cm layer, and in the Qinghai-Tibet Plateau semi-frigid semi-arid regions (R12) (5.06 ± 1.47 kg C m^−2^) for the 0–100 cm layer.

**Table 2:** Estimates of carbon density (kg C m^−2^) and storage (Pg C) in different regions of China.

Regions	Area	Vegetation						Soil				Vegetation + soil (0–100 cm)	
	(×10^4^ km^2^)	AGBC		BGBC		Total		0–20 cm		0–100 cm			
		Density	Storage	Density	Storage	Density	Storage	Density	Storage	Density	Storage	Density	Storage
		(kg C m^−2^)	(Pg C)	(kg C m^−2^)	(Pg C)	(kg C m^−2^)	(Pg C)	(kg C m^−2^)	(Pg C)	(kg C m^−2^)	(Pg C)	(kg C m^−2^)	(Pg C)
Cold humid regions (R1)	14.53	3.69±2.78	0.54±0.40	1.03±0.54	0.15±0.08	4.72±2.83	0.69±0.41	8.88±2.50	1.29±0.36	17.76±7.17	2.58±1.04	22.48±7.71	3.27±1.12
Temperate humid regions (R2)	52.66	2.83±1.77	1.49±0.93	0.70±0.44	0.37±0.23	3.53±1.83	1.86±0.96	6.11±1.41	3.22±0.74	14.01±3.46	7.38±1.82	17.54±3.91	9.24±2.06
Temperate semi-humid regions (R3)	29.83	1.28±1.10	0.38±0.33	0.42±0.23	0.13±0.07	1.70±1.12	0.51±0.34	4.14±1.39	1.23±0.42	10.20±3.78	3.04±1.13	11.90±3.94	3.55±1.18
Temperate semi-arid regions (R4)	78.84	0.27±0.22	0.21±0.17	0.44±0.29	0.34±0.23	0.71±0.37	0.56±0.29	2.72±1.39	2.14±1.10	6.96±3.36	5.49±2.65	7.67±3.38	6.05±2.67
Temperate arid regions (R5)	91.78	0.12±0.08	0.11±0.07	0.23±0.20	0.21±0.18	0.35±0.21	0.32±0.20	2.31±1.23	2.12±1.13	6.66±3.12	6.12±2.86	7.01±3.12	6.43±2.87
Warm temperate arid regions (R6)	86.02	0.14±0.11	0.12±0.10	0.22±0.12	0.19±0.10	0.35±0.17	0.30±0.14	2.12±1.10	1.83±0.94	8.36±3.83	7.19±3.30	8.72±3.83	7.50±3.30
Qinghai-Tibet plateau frigid arid regions (R7)	41.34	0.09±0.06	0.04±0.03	0.47±0.31	0.20±0.13	0.56±0.32	0.23±0.13	2.36±1.63	0.98±0.67	7.36±3.98	3.04±1.65	7.92±3.99	3.27±1.65
Warm temperate semi-humid regions (R8)	70.67	0.57±0.37	0.40±0.26	0.21±0.10	0.15±0.07	0.78±0.38	0.55±0.27	2.83±0.57	2.00±0.40	7.34±1.38	5.19±0.98	8.12±1.43	5.74±1.01
Warm temperate humid regions (R9)	3.49	0.75±0.53	0.03±0.02	0.22±0.13	0.01±0.00	0.97±0.55	0.03±0.02	2.80±0.66	0.10±0.02	7.39±1.63	0.26±0.06	8.36±1.71	0.29±0.06
Qinghai-Tibet plateau temperate arid regions (R10)	37.08	0.07±0.08	0.03±0.03	0.41±0.51	0.15±0.19	0.48±0.52	0.18±0.19	2.25±1.38	0.84±0.51	7.28±3.80	2.70±1.41	7.76±3.83	2.88±1.42
Qinghai-Tibet plateau temperate semi-arid regions (R11)	41.86	0.16±0.09	0.07±0.04	0.75±0.56	0.31±0.24	0.91±0.57	0.38±0.24	5.03±2.29	2.11±0.96	11.90±4.63	4.98±1.94	12.81±4.67	5.36±1.95
Qinghai-Tibet plateau subfrigid semi-arid regions (R12)	62.80	0.05±0.05	0.03±0.03	0.35±0.42	0.22±0.26	0.40±0.42	0.25±0.26	2.14±1.14	1.34±0.72	5.06±1.47	3.18±0.92	5.46±1.53	3.43±0.96
Qinghai-Tibet plateau subfrigid semi-humid regions (R13)	28.51	0.12±0.05	0.03±0.02	0.76±0.55	0.22±0.16	0.88±0.55	0.25±0.16	6.20±2.24	1.77±0.64	12.70±4.90	3.62±1.40	13.58±4.93	3.87±1.41
North subtropical humid regions (R14)	42.43	1.34±0.81	0.57±0.34	0.43±0.21	0.18±0.09	1.76±0.84	0.75±0.36	4.03±0.99	1.71±0.42	9.78±2.34	4.15±0.99	11.54±2.48	4.90±1.05
Qinghai-Tibet plateau temperate humid and semi-humid regions (R15)	37.71	1.28±0.78	0.48±0.29	0.83±0.36	0.31±0.14	2.11±0.86	0.79±0.32	6.36±1.47	2.40±0.56	11.58±3.51	4.37±1.33	13.68±3.61	5.16±1.36
Mid-subtropical humid regions (R16)	142.72	2.44±1.85	3.49±2.64	0.63±0.41	0.91±0.58	3.08±1.90	4.40±2.71	4.63±1.32	6.61±1.89	10.27±2.79	14.67±3.98	13.35±3.37	19.07±4.81
South subtropical humid regions (R17)	45.01	3.01±2.06	1.36±0.93	0.82±0.51	0.37±0.23	3.83±2.12	1.73±0.96	4.25±1.21	1.92±0.54	10.31±3.39	4.65±1.53	14.13±4.00	6.37±1.80
Tropical humid regions (R18)	18.17	3.53±3.29	0.64±0.60	1.01±0.74	0.18±0.14	4.53±3.37	0.83±0.61	3.99±1.46	0.73±0.27	10.73±4.05	1.95±0.74	15.27±5.27	2.78±0.96
Total	925.64	1.08±0.34	10.01±3.11	0.50±0.10	4.59±0.90	1.58±0.35	14.60±3.24	3.71±0.36	34.32±3.37	9.13±0.87	84.55±8.09	10.71±0.94	99.15±8.71

### Factors influencing the spatial distribution of C density

Climate, soil nutrients, and soil texture jointly explained 68.16% of total variance in spatial Veg-C density in the GLM analysis (Table 3). Climate (MAP and MAT) was the most important factor influencing this variation, explaining 50.49% of total variance (of which MAP explained 45.21% and MAT explained 5.28%). Climate, vegetation, soil nutrients, and soil texture jointly explained 95.31% and 90.01% of total variance in the spatial patterns of SOC density for the 0–20 cm and 0–100 cm soil layers, respectively (Table 4). Among these factors, vegetation and climate (especially MAT) were the most important factors explaining the spatial patterns of SOC density. Climate (especially MAT) significantly influenced the spatial patterns of SOC density in the topsoil (0–20 cm), but only minimally influenced it in the 0–100 cm soil layer. Climate was the most important factor influencing the spatial pattern of C density across ecosystems (Veg-C + SOC), explaining 34.72% of the total variance.

**Table 3:** The contribution of climate, soil texture, and soil nutrients to the spatial patterns of vegetation carbon (Veg-C) density in China’s terrestrial ecosystems. ^†^AGBC, above-ground biomass carbon; BGBC, below-ground biomass carbon; Veg-C, AGBC + BGBC; ^‡^***p <0.001, **p <0.01, *p <0.05, d.f., degree of freedom, m.s., mean square, % s.s., proportion of variance explained by a given variable.

	AGBC^†^	**d.f**.	**m.s**.	**% s.s**.	BGBC	**d.f**.	**m.s**.	**% s.s**.	Veg-C	**d.f**.	**m.s**.	**% s.s**.
Climate	Mean annul precipitation (MAP)	1	13.488**^‡^	46.56	MAP	1	0.270*	21.51	MAP	1	17.572**	45.21
	Mean annual temperature (MAT)	1	0.675	2.33	MAT	1	0.373**	29.72	MAT	1	2.051	5.28
Soil texture	Clay	1	0.420	1.45	Clay	1	0.005	0.40	Clay	1	0.514	1.32
	Silt	1	0.026	0.09	Silt	1	0.023	1.83	Silt	1	0.001	<0.01
	Sand	1	0.512	1.77	Sand	1	0.110	8.76	Sand	1	1.098	2.83
Soil nutrient	Soil nitrogen content (Soil N)	1	0.816	2.82	Soil N	1	0.003	0.24	Soil N	1	0.725	1.87
	Soil phosphorus content (Soil P)	1	0.181	0.62	Soil P	1	0.108	8.61	Soil P	1	0.571	1.47
	Soil potassium content (Soil K)	1	3.091	10.67	Soil K	1	0.054	4.30	Soil K	1	3.959	10.18
	Residuals	9	1.085	33.69	Residuals	9	0.034	24.62	Residuals	9	1.375	31.84

**Table 4:** Contribution of vegetation, climate, soil texture, and soil nutrients to the spatial patterns of soil organic carbon (SOC) density and ecosystem carbon density (Veg-C and SOC (0–100 cm)) in China’s terrestrial ecosystems.

	SOC (0–20 cm)	d.f.	m.s.	% s.s.	SOC (0–100 cm)	d.f.	m.s.	% s.s.	Veg-C + SOC (0–100 cm)	d.f.	m.s.	% s.s.
Vegetation	Vegetation C density (Veg-C)	1	27.515***^†^	45.73	Veg-C	1	86.276***	52.26				
Climate	Mean annul precipitation (MAP)	1	3.321*	5.52	MAP	1	15.544*	9.42	MAP	1	56.538	17.68
	Mean annual temperature (MAT)	1	9.956***	16.55	MAT	1	9.910	6.00	MAT	1	54.483	17.04
Soil texture	Clay	1	0.001	<0.01	Clay	1	0.846	0.51	Clay	1	8.673	2.71
	Silt	1	1.371	2.28	Silt	1	3.529	2.14	Silt	1	3.445	1.08
	Sand	1	2.698*	4.48	Sand	1	3.706	2.24	Sand	1	23.139	7.24
Soil nutrient	Soil nitrogen content (Soil N)	1	6.030**	10.02	Soil N	1	17.756*	10.76	Soil N	1	3.347	1.05
	Soil phosphorus content (Soil P)	1	5.160**	8.58	Soil P	1	11.005*	6.67	Soil P	1	1.138	0.36
	Soil potassium content (Soil K)	1	1.292	2.15	Soil K	1	0.021	0.01	Soil K	1	35.429	11.08
	Residuals	8	0.353	4.69	Residuals	8	2.062	9.99	Residuals	9	14.842	41.77

**Figure 2:**
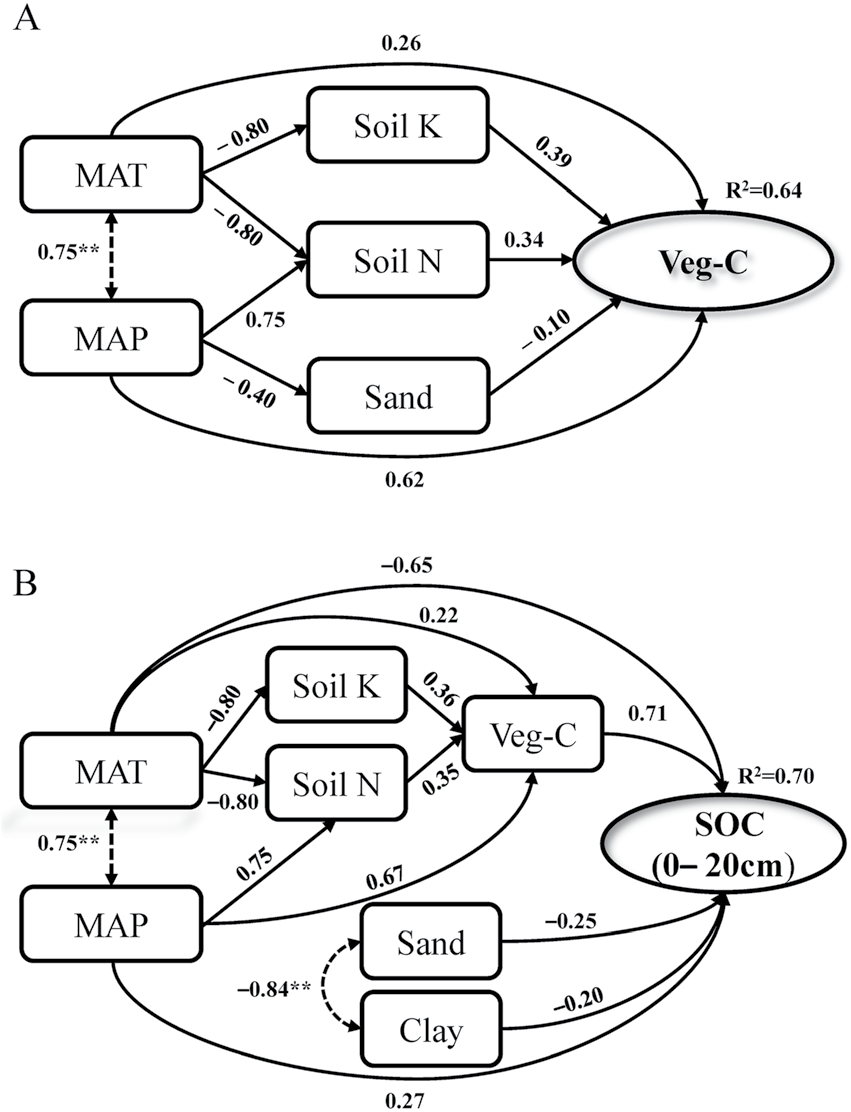
Mechanisms influencing the spatial patterns of vegetation carbon density (Veg-C) (**A**) and soil organic carbon density (SOC) in the topsoil (0–20 cm) (**B**) in China’s terrestrial ecosystems. Minus (−) values represent a negative impact in structural equation modeling (SEM). Dotted lines represent correlation coefficients and solid lines represent direct path coefficients. MAT, mean annual temperature (°C); MAP, mean annual precipitation (mm). Clay, sand, soil K and soil N represent soil clay content (%), soil sand content (%), soil potassium content (%), and soil nitrogen content (%), respectively. (**P < 0.01).

Climate, soil nutrients, and soil texture also explained 64% of variation in Veg-C density (Fig. 2A) when using path analysis, with MAP having the highest direct path coefficient (0.65). For the topsoil (0–20 cm), climate, vegetation, soil nutrients and soil texture explained 70% of variation in the spatial pattern of SOC density (Fig. 2B). The direct path coefficients were −0.65 and 0.27 for MAT and MAP, respectively, whereas the coefficient was 0.71 for Veg-C.

## Discussion

The present study estimated C storage in China’s terrestrial ecosystems as 99.15 ± 8.71 Pg C (Fig. 1; Fig. 2). Our estimate is similar to that obtained by Li *et al*. (2004), but lower than that obtained by Fang *et al*.^57^, Peng and Apps^11^, and Ni^18,19^ (Table 1). Differences in vegetation and soil datasets appear to be the main factors explaining variation in the C density of China’s terrestrial ecosystems (10.64–20.95 kg C m^−2^; Table 1). Most previous studies at a national scale used national inventory (forest and grassland) data and remote sensing data to estimate Veg-C storage^12,21,27,34^, and used Second National Soil Survey (1979–1985) data to calculate SOC storage^26,32,40,41^. In contrast, we collected a large amount of field-measured data for Veg-C and SOC (2004–2014). Compared with previous studies, our datasets contain the most recent and comprehensive information, facilitating more accurate estimates.

Differences in the methods used to estimate C storage values might partly contribute to the large uncertainty that exists at large scales^28,58^. Most previous studies calculated Veg-C storage and SOC storage based on ecosystem type, vegetation type, or soil type, separately^6,25,26,41^. In particular, the current study incorporated information on climate, vegetation, and land use. Thus, our results provide the first estimate of Veg-C and SOC storage in each region for each ecosystem type, which was then summed up to the national scale to generate more accurate estimates. Furthermore, different key parameters selected for evaluation (e.g., plant C content, soil bulk density, soil depth, and areas) might also cause large uncertainties in C storage estimates^47,49,50,58^. Our estimates of SOC storage were lower than those obtained by Fang *et al*.^38^ and higher than those obtained by Pan^39^, but were similar to those obtained by other studies^12,26,32,40,42,45^. Of note, vegetation in cropland and litter was excluded from this analysis, but was previously reported as 2.00 Pg C^43^ and 0.52 Pg C^59^, respectively, for China. To some extent, the different sampling time might lead to uncertainties in estimation accuracy. In future, we need to take the sampling time into consideration, in parallel to improving the precision of C estimation by developing observation techniques and increasing observation frequency and parameters (e.g., soil bulk density, soil gravel content). Therefore, field investigations in the northwest regions need to be strengthened, because fewer samples have been collected in these regions.

For forest ecosystems, Veg-C storage (11.49 ± 3.18 Pg C) estimates were higher in this study compared to those obtained by Zhou *et al*.^21^ (6.20 Pg C), Xu *et al*.^27^ (5.51 Pg C), and Li *et al*.^16^ (7.81 Pg C), which were calculated based on previous national forest inventory data. Our estimates of SOC storage (22.59 ± 4.40 Pg C) were also higher than those obtained in most previous studies. The higher estimates obtained here might be due to the implementation of key ecological restoration projects (e.g., Three-North Shelter Forest Program 4th Phase, Yangtze River Shelter Forest Project and Zhujiang River Shelter Forest Project 2nd Phase, Natural Forest Protection Project), which have contributed to C sequestration in the vegetation and soil in recent decades^3,60,61^. For grassland ecosystems, Veg-C and SOC storage were 1.94 ± 0.55 and 23.75 ± 4.68 Pg C, with an average density of 0.69 ± 0.20 and 8.47 ± 1.67 kg C m^−2^, respectively. Although our estimates of Veg-C density in grassland ecosystems were similar to those of previous studies, a large difference in C storage was detected, mainly due to the large difference in the surface area covered by this system^14,35^. For cropland ecosystems, SOC density in the 0–100 cm soil layer (8.85 ± 1.17 kg C m^−2^) was lower than average SOC density for China (9.13 ± 0.87 kg C m^−2^). This difference might be explained by the influence of intensive and long-term agricultural activity in China^28,41^. Of note, there are limited field data for shrub ecosystems; thus, the estimates of Veg-C and SOC storage in shrub ecosystems have high uncertainty.

Both GLM analysis and path analysis showed that climate, soil nutrients, and soil texture significantly influenced the spatial pattern of Veg-C and SOC density in China’s terrestrial ecosystems, with climate being the most important factor. Interestingly, we found that climate influenced the spatial pattern of Veg-C and SOC density through different processes and approaches. Specifically, MAP was the most important factor explaining the spatial pattern of Veg-C density, solely explaining 45.21% (GLM analysis) or 62% (path coefficient, path analysis) of variation. In contrast, MAT only explained 5.28% (GLM analysis) or 26% (path coefficient, path analysis) of variation for this parameter (Table 4 and Fig. 2). Some studies have reported that MAT and MAP affect net primary productivity and the spatial distribution of vegetation through direct and indirect impacts on water demand, water balance, and vegetation photosynthesis^53,62–64^. At regional and global scales, MAP has a more significant influence on the net primary productivity of vegetation than MAT^64,65^. The spatial patterns of Veg-C density in China are roughly consistent with China’s precipitation patterns. Higher precipitation leads to an increase in vegetation productivity and, thus, an increase in Veg-C density. This phenomenon might partially explain why Veg-C density is higher in cold humid regions (R1) and temperate humid regions (R2) than in some warmer regions of China, such as warm temperate arid regions (R6) and north subtropical humid regions (R14). Therefore, it is necessary for policy- makers in China to consider which climate factors influence Veg-C when they designate locations and select of tree or grass species for reforestation and returning croplands to forest and grassland in future ventures. Areas with high Veg-C should be protected and maintained (e.g., cold humid regions (R1), temperate humid regions (R2), south subtropical humid regions (R17), and tropical humid regions (R18)), whereas areas where Veg-C is likely to increase should be selected for reforestation or protection.

Besides vegetation, MAT was the most important factor influencing the spatial patterns of SOC density for the topsoil (0–20 cm), whereas the contribution of MAP was relatively small. Several studies have demonstrated that climate exerts significant impacts on the spatial patterns of SOC density, reflecting the balance between SOM inputs from plant production and outputs through decomposition in soil^47,49,52,53,66–70^. In general, new SOM input to the soil mainly originates from litterfall and rhizodeposition, which tend to be positively related to vegetation productivity (influenced by MAT and MAP, collectively), whereas SOM decomposition is mainly controlled by temperature and soil moisture^52,53,68,69^, with the influence of temperature on SOM decomposition being more obvious^53,71^. In brief, SOC density in the topsoil (0–20 cm) reflects the stronger effect of MAT on SOM decomposition, with SOC density increasing from tropical to cold temperate zones. However, for the 0–100 cm soil layer, MAT has a relatively small influence on the spatial pattern of SOC, because soil condition is relatively stable at this range (0–100 cm), and MAT impacts SOC decomposition less with increasing soil depth^49^. These findings indicate that areas with higher SOM input and relatively lower SOM decomposition accumulate more SOC. Such areas should be prioritized for protection. Meanwhile, we should keep eyes on the dynamics change of SOC storage, especially for the region with high SOC density but being under threats, such as land use change (a conversion from forest or grassland to cropland), deforestation, and overgrazing. Furthermore, many studies have also showed that soil in cold regions is more sensitive to temperature^71^; thus, policy- makers in China should strengthen land management (e.g., land use, fertilization) in areas with relatively low-temperature to mitigate the negative influence of climate change.

As expected, climate, soil nutrients, and texture significantly influenced the spatial distribution of ecosystem C density (Veg-C + SOC), with climate being the most important factor. However, climate (MAT + MAP) appeared to have a weaker capacity to explain the observed spatial distribution (Table 4). Thus, policy-makers should focus on understanding how climate factors influence ecosystem C density (Veg-C + Soil-C) to increase C storage in terrestrial ecosystems through rational ecological restoration projects (e.g., reforestation, returning croplands to forest and grassland) and land management policy. Areas with high ecosystem C density should be treated as key protection regions. In comparison, areas with relative low Veg-C density but high SOC density (e.g., Qinghai-Tibet plateau temperate semi-arid regions (R11) and Qinghai-Tibet plateau subfrigid semi-humid regions (R13)), require reasonable land use management and vegetation protection measures to maintain the current status and to increase Veg-C and SOC storage.

Our findings provide a more robust estimate of ecosystem C storage, and reveal the causes underlying the spatial patterns of Veg-C and SOC density in terrestrial ecosystems. We explored how climate influences Veg-C density and SOC density at a national scale; however, the specific processes and mechanisms involved remain unclear at the large scale. To improve terrestrial C sequestration, future studies should focus on how climate (MAT *vs*. MAP) differentially affects Veg-C density and SOC density. In practice, policy- makers in China should implement ecological restoration projects and more rational land management in relation to the climate to maximize the potential capacity of China’s terrestrial ecosystems to offset anthropogenic CO_2_ emissions in the future.

## Materials and Methods

### Data sources

#### Data collection and compilation

**Figure 3:**
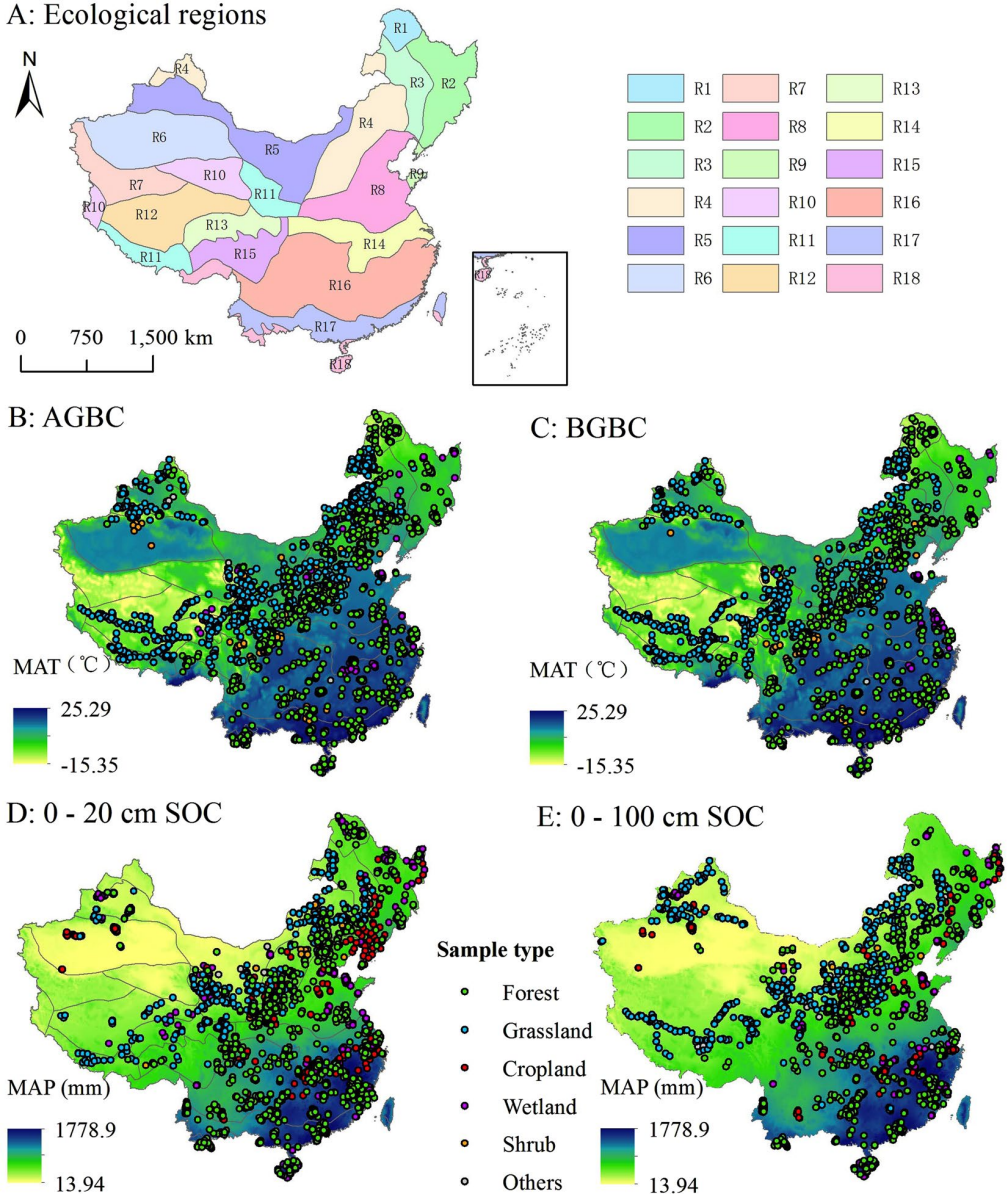
Regional division of China’s terrestrial ecosystems (**A**) and the distribution of sampling plots for vegetation (**B, C**) and soil samples (**D, E**). R1, Cold humid regions; R2, Temperate humid regions; R3, Temperate semi-humid regions; R4, Temperate semi-arid regions; R5, Temperate arid regions; R6, Warm temperate arid regions; R7, Qinghai-Tibet plateau frigid arid regions; R8, Warm temperate semi-humid regions; R9, Warm temperate humid regions; R10, Qinghai-Tibet plateau temperate arid regions; R11, Qinghai-Tibet plateau temperate semi-arid regions; R12, Qinghai-Tibet plateau subfrigid semi-arid regions; R13, Qinghai-Tibet plateau subfrigid semi-humid regions; R14, North subtropical humid regions; R15, Qinghai-Tibet plateau temperate humid and semi-humid regions; R16, Mid-subtropical humid regions; R17, South subtropical humid regions; R18, Tropical humid regions. AGBC, Above-ground biomass carbon; BGBC, Below-ground biomass carbon; SOC, soil organic carbon. The figure was generated using ArcGIS software (version 10.0, ESRI, USA).

We collected information on vegetation and soil through two approaches: (1) field-measured results from papers publicly published from 2004 to 2014 in the China National Knowledge Infrastructure (CNKI) (http://www.cnki.net) and Institute for Scientific Information (ISI) (http://apps.webofknowledge.com) databases (Supplementary Appx. S1–S5), using “SOC”, “biomass”, “C density” or “C storage” as key words; and (2) unpublished field-measured data obtained by personal correspondence (Supplementary Appx. S6–S9). The collected papers were further screened based on the following criteria: (1) data on biomass/biomass C density and SOC content/concentration should be obtained through field investigations; (2) field investigations should have been performed after 2000; and (3) biomass and SOC determination methods should be comparable. A total of 1036 papers were selected. The collected data encompassed the main ecosystems in China, including forest, grassland, cropland, wetland, and shrub ecosystems (Supplementary Appx. S1–S5). Specifically, the collected data included records for 7927 vegetation samples (4485 samples for above-ground biomass (AGB) and 3442 samples for below-ground biomass (BGB)) and 7683 soil samples (4536 samples for the 0–20 cm soil layer, and 3147 samples for the 0–100 cm soil layer; Fig. 3). Vegetation C storage in croplands was not considered in this study owing to periodic harvests.

**Figure 4:**
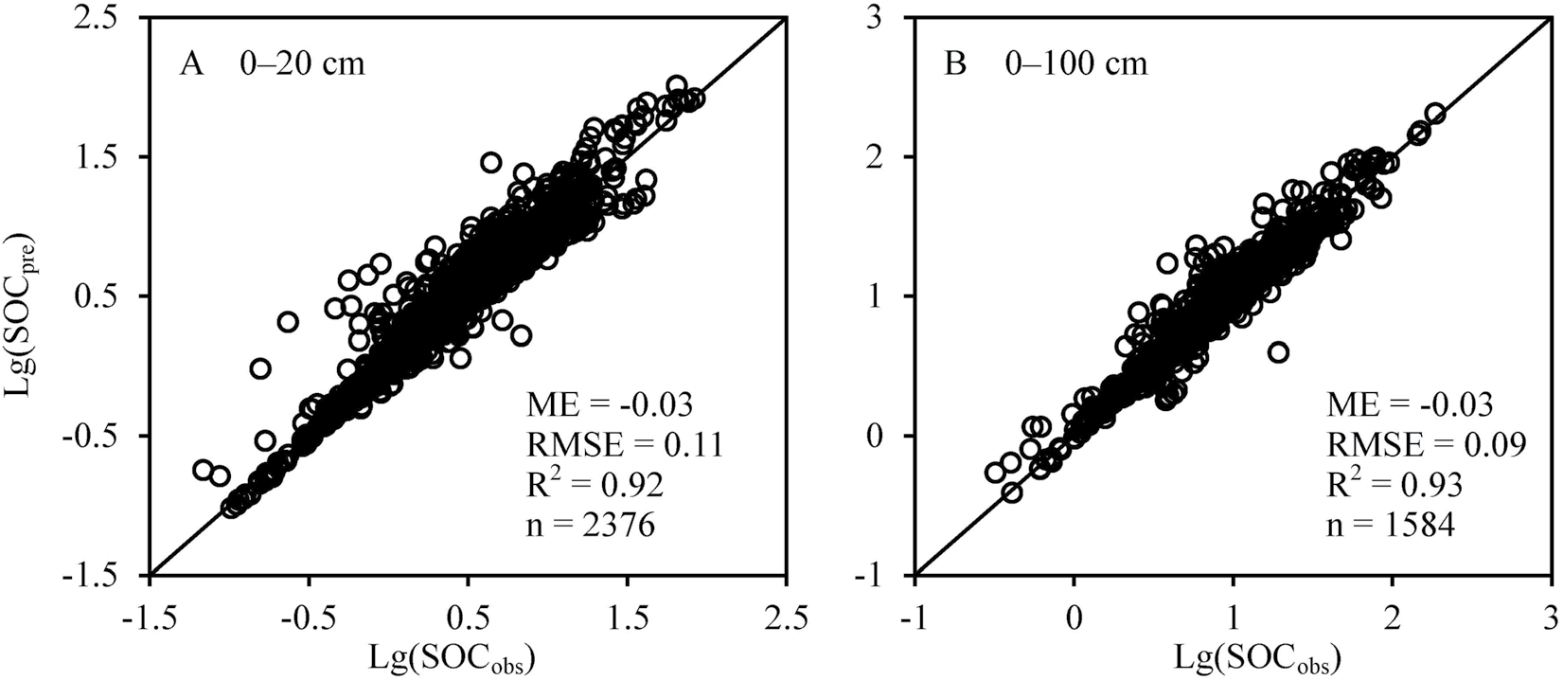
Comparison between observed and predicted soil organic carbon (SOC) density (kg C m^−2^) by pedotransfer function. These data of observed SOC density and predicted SOC density were taken a logarithmic transformation to reduce the impact of a few high-value data. SOC_obs_ (kg C m^−2^) represented the observed SOC density, SOC_pre_ (kg C m^−2^) represented the observed SOC density, ME represented mean error, and RMSE represented root mean square error.

For vegetation and soil samples that had no detailed geographical information, we extracted their latitude and longitude with a digital map (http://map.tianditu.com), based on the description of the study site. Reported field measurements of above-ground biomass C (AGBC) and below-ground biomass C (BGBC) density were used directly. For samples that were reported only as vegetation biomass (AGB or BGB), a coefficient of 0.45 was used to convert vegetation biomass density to C density (kg C m^−2^)^57^. When SOC density (kg C m^−2^) was not reported in the original studies, it was calculated using Eq. 1:

1 $${\rm{SOC}}\,{\rm{density}}={\sum }_{i=1}^{{\rm{n}}}SO{C}_{i}\times B{D}_{i}\times {D}_{i}\times (1-{\delta }_{i})\times 0.1$$

where *SOC*_*i*_, *BD*_*i*_, *D*_*i*_, and *δ*_*i*_ represented SOC content (%), bulk density (g cm^−3^), soil depth (cm), and the volumetric percentage of the fraction >2 mm (%), respectively, in soil layer *i*; and *n* was the number of soil layers. SOM was converted to SOC using a constant of 0.58^26^. A classic pedotransfer function was used to estimate bulk density from SOC concentration, when records were not available^28^. To validate the prediction accuracy of the pedotransfer function, soil samples with the data of bulk density and SOC content were used to calculate the observed SOC density and the predicted SOC density. Then, these data were taken a logarithmic transformation to reduce the impact of a few high-value data, and compared by the 1:1 relationship, mean error (ME), and root mean square error (RMSE)^72,73^. The result showed that the pedotransfer function can well predict SOC density, with the ME and RMSE equal to −0.03 and 0.11 kg C m^−2^ for 0–20 cm soil layer (R^2^ = 0.92), and −0.03 and 0.09 kg C m^−2^ for 0–100 cm soil layer (R^2^ = 0.93), respectively (Fig. 4). In this study, the volumetric percentage of the fraction > 2 mm (*δ*_*i*_,%) was 0 for soil for which bulk density records were available. For soil with no records of rock fragment and bulk density, the mean value of the rock fragment volume was used to substitute the same soil type.

#### Division of ecological regions

China spans a huge geographic and environmental range, extending from tropical to boreal zones, from rain forests to desert^74^. Considering China’s broad environmental gradients and highly heterogeneous topography, China’s terrestrial ecosystems were divided into 18 zones based on climate and topography^75^ to investigate differences in C storage among different regions. The zones were designated as follows: cold humid regions (R1), temperate humid regions (R2), temperate semi-humid regions(R3), temperate semi-arid regions (R4), temperate arid regions (R5), warm temperate arid regions (R6), Qinghai-Tibet plateau frigid arid regions (R7), warm temperate semi-humid regions (R8), warm temperate humid regions (R9), Qinghai-Tibet plateau temperate arid regions (R10), Qinghai-Tibet plateau temperate semi-arid regions (R11), Qinghai-Tibet plateau subfrigid semi-arid regions (R12), Qinghai-Tibet plateau subfrigid semi-humid regions (R13), north subtropical humid regions (R14), Qinghai-Tibet plateau temperate humid and semi-humid regions (R15), mid-subtropical humid regions (R16), south subtropical humid regions (R17), and tropical humid regions (R18) (Fig. 3).

#### Data on climate and soil properties

Based on long-term temperature and precipitation monitoring data (1961–2010) from 722 meteorological stations in China, we obtained the mean annual temperature (MAT, °C) and mean annual precipitation (MAP, mm) for each region^76^. The Second National Soil Survey in China was used to provide data on soil nitrogen (Soil N, %), phosphorus (Soil P, %), and potassium (Soil K, %) content, which represent soil nutrients, and the proportion of soil clay (%), silt (%), and sand (%), which represent soil texture. The spatial resolution of the Second National Soil Survey is 10 km × 10 km. Areas of different ecosystems (forest, grassland, cropland, shrub, wetland, and others) for each region were extracted from the Chinese land cover data (2010)^77^. The area of terrestrial ecosystems in China, except for Taiwan Province and inland waters, covered approximately 9.25 × 10^6^ km^2^.

### Calculating vegetation and soil C storage at different scales

Two steps were used to estimate C storage in China’s terrestrial ecosystems (see Supplementary Fig. S1 for details). The first step was from point scale to regional scale, and the second was from regional scale to national scale. For the first step, we estimated the Veg-C (AGBC and BGBC) and SOC density and storage of different ecosystems in each ecological region. Then, we used the Veg-C and SOC storage of different ecosystems in an ecological region to estimate the C storage in each region. For ecological regions where the sample number of one ecosystem was less than 10, or the spatial distribution of samples was extremely uneven (i.e., samples were concentrated in a single area), we combined the samples of the same ecosystem in adjacent regions with similar climatic conditions to estimate C density.

For the second step, we used the Veg-C and SOC storage of different ecological regions to estimate C storage at the national scale. The C storage of vegetation, soil, and terrestrial ecosystems (vegetation + soil) in China was calculated as:

2 $$\mathrm{Veg}-C\,{\rm{storage}}={\sum }_{i=1}^{m}{\sum }_{j=1}^{n}(AGBC{D}_{ij}+BGBC{D}_{ij})\times {S}_{ij}$$

3 $${\rm{SOC}}\,{\rm{storage}}={\sum }_{i=1}^{m}{\sum }_{j=1}^{n}(SOC{D}_{ij}\times {S}_{ij})$$

4 $${\rm{Ecosystem}}\,{\rm{C}}\,{\rm{storage}}={\sum }_{i=1}^{m}(Veg-C{S}_{i}+SOC{S}_{i})$$

where *m* and *n* are the number of ecological regions and ecosystems. *AGBCD*_*ij*_, *BGBCD*_*ij*_, and *SOCD*_*ij*_ are AGBC density, BGBC density, and SOC density of ecosystem *j* in ecological region *i*, respectively. *S*_*ij*_ is the surface area of ecosystem *j* in ecological region *i*. *Veg-CS*_*i*_ and *SOCS*_*i*_ are C storage in the vegetation and soil of region *i*, respectively. For the purposes of this study, we estimated SOC storage at two soil depths (0–20 cm and 0–100 cm). SOC storage at the 0–100 cm soil depth was used to calculate C storage in terrestrial ecosystems, whereas SOC storage at 0–20 cm was used to characterize C storage in the surface soil.

### Statistical analysis

A general linear model (GLM) was used to assess variation in AGBC, BGBC, Veg-C (AGBC + BGBC), SOC (0–20 cm and 0–100 cm soil layers), and the ecosystem (Veg-C + SOC) explained by climate (MAT and MAP), soil nutrient (soil N, P, and K), and soil texture (clay, silt, and sand). Path analysis was used to investigate the main factors influencing the spatial patterns of Veg-C and SOC (0–20 cm soil layer) quantitatively. For Veg-C storage, the analyzed factors included climate (MAT and MAP), soil nutrient (soil N, P, and K), and soil texture (clay, silt, and sand); for SOC storage, the analyzed factors included SOM input (Veg-C), climate (MAT and MAP) and soil properties (clay, silt, and sand, soil N, P, and K). Because some predictors are correlated, we used path analysis to determine significant direct predictors for Veg-C and SOC, as well as indirect pathways. The initial models of path analysis for Veg-C and SOC density were fully identified, including all possible causal links between observed predictors (e.g., MAT and MAP) and response variables (e.g., soil N, K), and all correlations among predictors. These models provided estimates and significance tests for all potential paths among variables. We trimmed the initial models by retaining significant direct predictor variables. The fitted significance and goodness of the trimmed models were assessed with the following indices: *χ*^2^ test, Bentler’s comparative fit index (CFI) (>0.95), and the standardized root mean residual (<0.08)^53,78,79^. We built path diagrams using standardized path coefficients between the predictors and response variables, and correlation coefficients between predictors. The GLM analysis was conducted using the lm function in the R package (R project 3.1.2, R development team, 2014). Path analysis was performed by SPSS software (version 18.0, Chicago, IL, USA) (Supplementary Appx. S10). Significant differences were defined at the *p* = 0.05 level.

## Electronic supplementary material

Supplementary materials

Supplementary data
