# Supplementary material for: Carbon storage in China’s terrestrial ecosystems: A synthesis
Source: Sci Rep. 2018 Feb 12;8:2806. doi: 10.1038/s41598-018-20764-9 (PMC5809558; doi:10.1038/s41598-018-20764-9)
Supplement: Supplementary file 1 — Supplementary materials [file 41598_2018_20764_MOESM1_ESM.doc]

**Carbon storage in China’s terrestrial ecosystems: A synthesis**

**Running title:** Carbon storage in China

Li Xu ^1,2^, Guirui Yu ^1^,* , Nianpeng He ^1^,*, Qiufeng Wang^1^, Yang Gao^1^, Ding Wen^1,2^, Shenggong Li ^1^, Shuli Niu ^1^, Jianping Ge ^3^,

^1^ Key Laboratory of Ecosystem Network Observation and Modeling, Institute of Geographic Sciences and Natural Resources Research, Chinese Academy of Sciences, Beijing 100101, China

^2^ College of Resources and Environment, University of Chinese Academy of Sciences, Beijing 100049, China

^3^ College of Life Sciences, Beijing Normal University, Beijing 100875, China

* For correspondence.

E-mail: [yugr@igsnrr.ac.cn](mailto:yugr@igsnrr.ac.cn) (G. Y.); [henp@igsnrr.ac.cn](mailto:henp@igsnrr.ac.cn) (N. H.)

Tel.: +861064889263

Fax: +861064889399

**Supplementary Fig. S1**

**
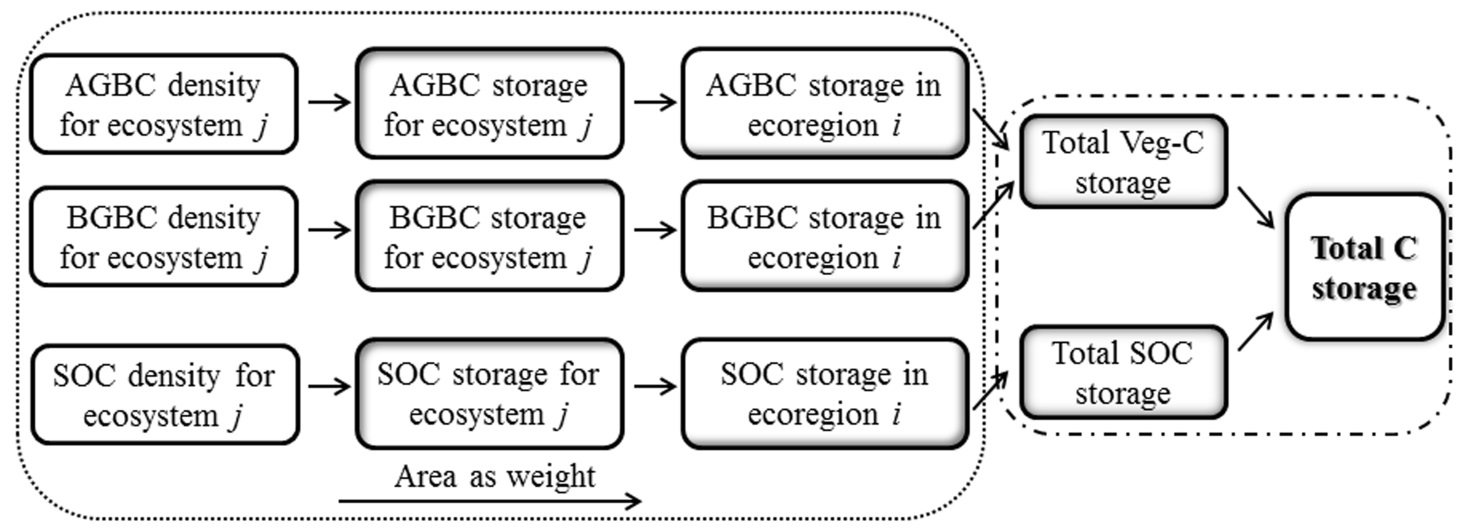
**

Fig.S1 Flow diagram of total C (Veg-C + SOC) storage calculation. AGBC, above-ground biomass carbon; BGBC, below-ground biomass carbon; SOC, soil organic carbon.

**Supplementary Fig. S2**

**
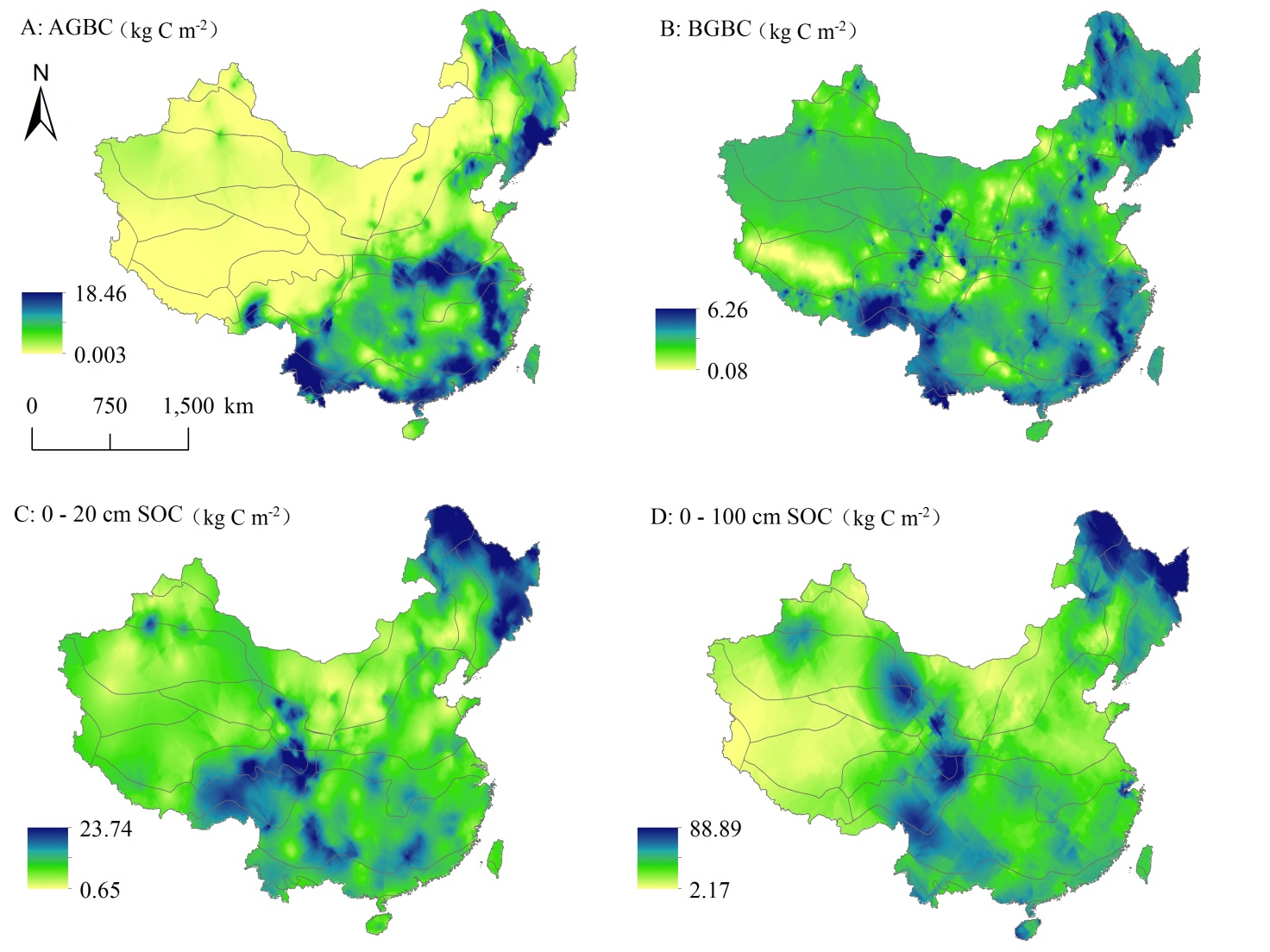
**

Fig.S2 Spatial distribution of vegetation carbon density (A and B) (kg C m^–2^) and soil organic carbon density (C and D) (kg C m^–2^) in China. AGBC, above-ground biomass carbon; BGBC, below-ground biomass carbon; SOC, soil organic carbon. The figures were generated using ArcGIS software (version 10.0, ESRI, USA).

**Supplementary Table S1**

Table S1 The estimates of carbon density (kg C m^-2^) and storage (Pg C) in different ecosystems in China

| Ecosystem | Area  (×10^4^km^2^) | Vegetation | | | | | | | |  | | SOC | | | | |  | Total | |
| --- | --- | --- | --- | --- | --- | --- | --- | --- | --- | --- | --- | --- | --- | --- | --- | --- | --- | --- | --- |
|  |  | AGBC^†^ | |  | BGBC | |  | Total | |  | 0–20 cm | | |  | 0–100 cm | |  |  | |
|  |  | Density  (kg C m^–2^) | Storage  (Pg C) |  | Density  (kg C m^–2^) | Storage  (Pg C) |  | Density  (kg C m^–2^) | Storage  (Pg C) |  | Density  (kg C m^–2^) | | Storage  (Pg C) |  | Density  (kg C m^–2^) | Storage  (Pg C) |  | Density  (kg C m^–2^) | Storage  (Pg C) |
| Forest | 195.89 | 4.77±1.58^*^ | 9.35±3.11 |  | 1.09±0.35 | 2.14±0.69 |  | 5.86±1.62 | 11.49±3.18 |  | 5.27±1.05 | | 10.32±2.06 |  | 11.53±2.24 | 22.59±4.40 |  | 17.40±2.77 | 34.08±5.43 |
| Grassland | 280.44 | 0.06±0.02 | 0.18±0.06 |  | 0.63±0.20 | 1.76±0.55 |  | 0.69±0.20 | 1.94±0.55 |  | 3.63±0.79 | | 10.18±2.22 |  | 8.47±1.67 | 23.75±4.68 |  | 9.16±1.68 | 25.69±4.71 |
| Cropland | 171.53 | —^‡^ | — |  | — | — |  | — | — |  | 3.28±0.46 | | 5.63±0.78 |  | 8.85±1.17 | 15.17±2.00 |  | 8.85±1.17 | 15.17±2.00 |
| Wetland | 14.46 | 0.32±0.08 | 0.05±0.01 |  | 1.08±0.42 | 0.16±0.06 |  | 1.40±0.43 | 0.20±0.06 |  | 8.10±1.67 | | 1.17±0.24 |  | 23.60±5.51 | 3.41±0.80 |  | 25.01±5.53 | 3.62±0.80 |
| Shrub | 77.69 | 0.34±0.09 | 0.26±0.07 |  | 0.23±0.09 | 0.18±0.07 |  | 0.56±0.13 | 0.44±0.10 |  | 4.13±0.71 | | 3.21±0.56 |  | 8.98±2.47 | 6.98±1.92 |  | 9.55±2.48 | 7.42±1.92 |
| Others | 185.63 | 0.10±0.06 | 0.18±0.11 |  | 0.19±0.06 | 0.35±0.11 |  | 0.29±0.09 | 0.53±0.16 |  | 2.04±0.59 | | 3.79±1.09 |  | 6.81±2.15 | 12.65±3.98 |  | 7.10±2.15 | 13.18±3.99 |
| Total | 925.64 | 1.08±0.34 | 10.01±3.11 |  | 0.50±0.10 | 4.59±0.90 |  | 1.58±0.35 | 14.60±3.24 |  | 3.71±0.36 | | 34.31±3.37 |  | 9.13±0.87 | 84.55±8.09 |  | 10.71±0.94 | 99.15±8.71 |

^†^ AGBC, above-ground biomass carbon; BGBC, below-ground biomass carbon; SOC, soil organic carbon.

^‡^ Vegetation carbon storage in cropland was not calculated in this study.

^*^ According to error calculation methods (Ku, 1966; Phillip et al., 2000), we added the errors estimates of different ecosystem C storage and the C storage in China. The standard error (SE) is calculated using

SE=
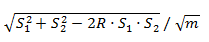


where
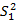
 and
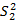
 C sampling variance of C storage for different ecosystems; *R* is the correlation of storage between the different ecosystems; and *m* is the number of samples. Here, different ecosystems were assumed to be statistically independent (*R*=0), and *m*=1 since there were unique value of C storage for different ecosystem.

**Reference:**

1. Ku, H. H. (1966), Notes on the use of propagation of error formulas, *Journal of Research of the National Bureau of Standards*, 70(4), 263-273.
2. Phillips, D. L., S. L. Brown, P. E. Schroeder, and R. A. Birdsey (2000), Toward error analysis of large-scale forest carbon budget. *Global Ecology and Biogeography,* 9(4), 305-313.

**Supplementary Table S2**

Table S2 The estimates of carbon density (kg C m^–2^) and storage (Tg C) in different ecosystems at the scale of 18 ecoregions

| Ecosystem | Regions | Area  (×10^4^km^2^) | Vegetation | | | | | | Soil | | | | Total | |
| --- | --- | --- | --- | --- | --- | --- | --- | --- | --- | --- | --- | --- | --- | --- |
|  |  |  | AGBC | | BGBC | | Total | | 0–20 cm | | 0–100 cm | |  | |
|  |  |  | Density  (kg C m^–2^) | Storage  (Tg C) | Density  (kg C m^–2^) | Storage  (Tg C) | Density  (kg C m^–2^) | Storage  (Tg C) | Density  (kg C m^–2^) | Storage  (Tg C) | Density  (kg C m^–2^) | Storage  (Tg C) | Density | Storage |
|  |  |  |  |  |  |  |  |  |  |  |  |  | (kg C m^–2^) | (Tg C) |
| Forest | R1^†^ | 11.758 | 4.47±3.43 | 526.64 | 1.18±0.67 | 139.18 | 5.66±3.50 | 665.81 | 8.48±3.04 | 996.50 | 17.33±8.30 | 2037.43 | 22.99±9.01 | 2703.24 |
| Forest | R2 | 26.548 | 5.58±3.51 | 1481.78 | 1.34±0.88 | 355.27 | 6.92±3.62 | 1837.05 | 7.55±2.49 | 2003.09 | 14.62±5.63 | 3882.55 | 21.54±6.70 | 5719.60 |
| Forest | R3 | 7.128 | 5.03±4.60 | 358.55 | 1.23±0.91 | 87.72 | 6.26±4.69 | 446.27 | 4.34±3.79 | 309.67 | 11.94±11.76 | 850.93 | 18.20±12.66 | 1297.20 |
| Forest | R4 | 5.956 | 2.74±2.82 | 162.93 | 0.67±0.67 | 39.65 | 3.40±2.90 | 202.59 | 2.65±2.42 | 157.93 | 6.55±4.84 | 389.96 | 9.95±5.64 | 592.55 |
| Forest | R5 | 1.194 | 3.73±3.38 | 44.49 | 0.60±0.61 | 7.12 | 4.32±3.43 | 51.61 | 3.79±3.55 | 48.23 | 10.27±11.94 | 122.68 | 14.59±12.42 | 174.28 |
| Forest | R6 | 1.226 | 3.75±3.40 | 45.99 | 0.60±0.61 | 7.27 | 4.35±3.45 | 53.26 | 3.62±3.50 | 44.35 | 10.27±11.94 | 125.90 | 14.62±12.43 | 179.17 |
| Forest | R7 | 0.055 | 3.75±3.40 | 2.07 | 0.64±0.63 | 0.36 | 4.40±3.46 | 2.43 | 3.62±3.50 | 2.00 | 10.27±11.94 | 5.67 | 14.67±12.43 | 8.09 |
| Forest | R8 | 11.376 | 3.12 ±2.30 | 354.76 | 0.79±0.57 | 89.92 | 3.91±2.37 | 444.68 | 3.90±2.35 | 443.86 | 8.75±5.19 | 995.38 | 12.66±5.70 | 1440.06 |
| Forest | R9 | 0.809 | 3.12±2.29 | 25.25 | 0.79±0.57 | 6.40 | 3.91±2.36 | 31.65 | 3.90±2.35 | 31.56 | 8.75±5.19 | 70.76 | 12.66±5.70 | 102.41 |
| Forest | R10 | 0.010 | 3.75±3.40 | 0.38 | 0.59±0.59 | 0.06 | 4.35±3.45 | 0.43 | 3.62±3.50 | 0.36 | 12.15±13.28 | 1.21 | 16.49±13.72 | 1.65 |
| Forest | R11 | 0.851 | 3.72±3.37 | 31.64 | 0.59±0.58 | 5.03 | 4.31±3.42 | 36.68 | 7.82±3.64 | 66.57 | 19.71±9.81 | 167.73 | 24.03±10.39 | 204.41 |
| Forest | R12 | 0.002 | 3.72±3.37 | 0.08 | 0.59±0.58 | 0.01 | 4.31±3.42 | 0.10 | 8.01±3.33 | 0.18 | 19.71±9.81 | 0.45 | 24.03±10.39 | 0.55 |
| Forest | R13 | 0.175 | 5.48±3.59 | 9.57 | 0.59±0.58 | 1.03 | 6.07±3.64 | 10.60 | 8.01±3.33 | 13.98 | 19.71±9.81 | 34.42 | 25.79±10.46 | 45.02 |
| Forest | R14 | 11.440 | 4.65 ±3.01 | 532.43 | 0.96±0.71 | 109.69 | 5.61±3.09 | 642.12 | 4.35±2.17 | 497.24 | 9.25±3.42 | 1058.13 | 14.86±4.61 | 1700.25 |
| Forest | R15 | 8.154 | 5.48±3.59 | 446.94 | 1.55±0.81 | 126.23 | 7.03±3.68 | 573.17 | 8.18±3.05 | 667.09 | 16.33±7.36 | 1331.85 | 23.36±8.23 | 1905.01 |
| Forest | R16 | 72.016 | 4.68±3.67 | 3369.39 | 0.97±0.79 | 700.93 | 5.65±3.75 | 4070.33 | 4.89±2.43 | 3520.67 | 10.43±4.77 | 7513.55 | 16.09±6.07 | 11583.88 |
| Forest | R17 | 25.422 | 5.20±3.65 | 1323.18 | 1.21±0.91 | 307.13 | 6.41±3.76 | 1630.31 | 4.24±2.01 | 1077.70 | 10.64±5.70 | 2705.15 | 17.05±6.83 | 4335.46 |
| Forest | R18 | 11.772 | 5.36±5.08 | 630.40 | 1.34±1.15 | 157.74 | 6.70±5.21 | 788.15 | 3.76±2.20 | 442.56 | 11.00±6.09 | 1294.59 | 17.69±8.01 | 2082.74 |
| Grassland | R1 | 0.178 | 0.08±0.08 | 0.15 | 0.48±0.42 | 0.85 | 0.56±0.43 | 0.99 | 4.29±2.70 | 7.64 | 8.71±5.60 | 15.50 | 9.26±5.62 | 16.50 |
| Grassland | R2 | 0.180 | 0.08±0.08 | 0.15 | 0.48±0.42 | 0.86 | 0.56±0.43 | 1.01 | 4.29±2.70 | 7.74 | 8.71±5.60 | 15.71 | 9.26±5.62 | 16.72 |
| Grassland | R3 | 5.009 | 0.08±0.07 | 4.00 | 0.48±0.42 | 23.81 | 0.56±0.43 | 27.81 | 4.17±2.64 | 208.82 | 8.22±5.40 | 411.89 | 8.78±5.42 | 439.70 |
| Grassland | R4 | 50.098 | 0.07±0.07 | 34.93 | 0.58±0.46 | 290.34 | 0.65±0.46 | 325.28 | 3.21±2.14 | 1609.47 | 7.91±5.12 | 3963.59 | 8.56±5.14 | 4288.88 |
| Grassland | R5 | 40.587 | 0.06±0.07 | 23.19 | 0.36±0.43 | 146.13 | 0.42±0.44 | 169.31 | 2.50±2.47 | 1012.97 | 6.62±5.23 | 2687.45 | 7.04±5.25 | 2856.76 |
| Grassland | R6 | 12.271 | 0.07±0.03 | 8.66 | 0.74±0.54 | 90.35 | 0.81±0.54 | 99.00 | 2.58±2.67 | 316.60 | 16.33±4.43 | 2004.02 | 17.14±4.46 | 2103.02 |
| Grassland | R7 | 23.522 | 0.07±0.03 | 16.60 | 0.74±0.54 | 173.17 | 0.81±0.54 | 189.77 | 2.58±2.67 | 606.85 | 7.59±5.90 | 1785.55 | 8.40±5.93 | 1975.32 |
| Grassland | R8 | 5.661 | 0.13±0.11 | 7.47 | 0.57±0.41 | 32.01 | 0.70±0.42 | 39.48 | 2.64±1.66 | 149.23 | 6.24±2.47 | 353.47 | 6.94±2.51 | 392.94 |
| Grassland | R9 | 0.108 | 0.13± 0.11 | 0.14 | 0.57±0.41 | 0.61 | 0.70±0.42 | 0.76 | 2.64±1.66 | 2.86 | 6.24±2.47 | 6.77 | 6.94±2.51 | 7.53 |
| Grassland | R10 | 15.688 | 0.05±0.03 | 7.88 | 0.79±1.20 | 123.38 | 0.84±1.20 | 131.26 | 2.58±2.67 | 404.74 | 7.59±5.90 | 1190.87 | 8.43±6.02 | 1322.13 |
| Grassland | R11 | 26.556 | 0.06±0.07 | 16.84 | 1.00±0.88 | 265.33 | 1.06±0.89 | 282.17 | 5.78±3.58 | 1534.48 | 12.24±7.05 | 3249.72 | 13.30±7.11 | 3531.89 |
| Grassland | R12 | 51.164 | 0.03±0.06 | 16.47 | 0.34±0.51 | 172.22 | 0.37±0.51 | 188.69 | 2.16±1.40 | 1107.36 | 3.78±1.73 | 1932.01 | 4.14±1.81 | 2120.70 |
| Grassland | R13 | 20.440 | 0.07±0.06 | 14.07 | 0.74±0.73 | 150.43 | 0.80±0.73 | 164.50 | 6.80±3.07 | 1390.18 | 12.30±6.71 | 2514.36 | 13.11±6.75 | 2678.86 |
| Grassland | R14 | 0.801 | 0.10±0.09 | 0.79 | 1.00±0.74 | 8.04 | 1.10±0.75 | 8.83 | 5.14±2.88 | 41.15 | 12.74±6.04 | 102.01 | 13.84±6.09 | 110.83 |
| Grassland | R15 | 16.576 | 0.09±0.09 | 15.68 | 1.02±0.72 | 169.07 | 1.11±0.73 | 184.75 | 7.27±2.85 | 1205.45 | 12.25±6.47 | 2031.29 | 13.37±6.51 | 2216.03 |
| Grassland | R16 | 8.268 | 0.10±0.09 | 7.82 | 1.00±0.74 | 82.30 | 1.10±0.75 | 90.80 | 5.01±2.62 | 414.38 | 12.74±6.25 | 1053.09 | 13.84±6.30 | 1143.89 |
| Grassland | R17 | 2.111 | 0.10±0.09 | 2.00 | 1.00±0.74 | 21.19 | 1.10±0.75 | 23.19 | 4.92±2.57 | 103.96 | 13.09±6.20 | 276.29 | 14.19±6.25 | 299.48 |
| Grassland | R18 | 1.219 | 0.10±0.09 | 1.15 | 1.00±0.74 | 12.24 | 1.10±0.75 | 13.39 | 4.92±2.57 | 60.05 | 13.09±6.20 | 159.59 | 14.19±6.25 | 172.98 |
| Cropland | R1 | 0.231 | – ^‡^ | – | – | – | – | – | 4.46±1.44 | 10.32 | 13.29±4.39 | 30.71 | 13.29±4.39 | 30.71 |
| Cropland | R2 | 21.478 | – | – | – | – | – | – | 4.46±1.44 | 958.71 | 13.29±4.39 | 2854.18 | 13.29±4.39 | 2854.18 |
| Cropland | R3 | 13.383 | – | – | – | – | – | – | 3.40±1.83 | 455.47 | 8.57±3.90 | 1146.26 | 8.57±3.90 | 1146.26 |
| Cropland | R4 | 13.138 | – | – | – | – | – | – | 1.82±1.36 | 239.24 | 5.37±4.28 | 705.53 | 5.37±4.28 | 705.53 |
| Cropland | R5 | 7.918 | – | – | – | – | – | – | 1.89±0.77 | 149.47 | 4.52±1.92 | 357.89 | 4.52±1.92 | 357.89 |
| Cropland | R6 | 3.676 | – | – | – | – | – | – | 1.96±1.12 | 71.87 | 6.17±3.30 | 226.68 | 6.17±3.30 | 226.68 |
| Cropland | R7 | 0.131 | – | – | – | – | – | – | 1.96±1.12 | 2.56 | 6.17±3.30 | 8.07 | 6.17±3.30 | 8.07 |
| Cropland | R8 | 38.540 | – | – | – | – | – | – | 2.43±0.65 | 938.06 | 7.26±1.83 | 2797.85 | 7.26±1.83 | 2797.85 |
| Cropland | R9 | 1.968 | – | – | – | – | – | – | 2.44±0.65 | 48.08 | 7.26±1.83 | 142.84 | 7.26±1.83 | 142.84 |
| Cropland | R10 | 0.070 | – | – | – | – | – | – | 1.96±1.12 | 1.37 | 6.17±3.30 | 4.31 | 6.17±3.30 | 4.31 |
| Cropland | R11 | 3.269 | – | – | – | – | – | – | 2.78±1.89 | 90.80 | 9.16±5.32 | 299.35 | 9.16±5.32 | 299.35 |
| Cropland | R12 | 0.005 | – | – | – | – | – | – | 2.86±1.81 | 0.14 | 9.16±5.32 | 0.44 | 9.16±5.32 | 0.44 |
| Cropland | R13 | 0.039 | – | – | – | – | – | – | 2.86±1.81 | 1.12 | 9.16±5.32 | 3.58 | 9.16±5.32 | 3.58 |
| Cropland | R14 | 20.618 | – | – | – | – | – | – | 3.86±1.55 | 796.55 | 10.25±4.01 | 2114.13 | 10.25±4.01 | 2114.13 |
| Cropland | R15 | 0.514 | – | – | – | – | – | – | 2.86±1.81 | 14.70 | 9.16±5.32 | 47.07 | 9.16±5.32 | 47.07 |
| Cropland | R16 | 34.622 | – | – | – | – | – | – | 3.98±1.40 | 1378.37 | 9.53±3.13 | 3298.14 | 9.53±3.13 | 3298.14 |
| Cropland | R17 | 9.522 | – | – | – | – | – | – | 4.00±1.41 | 381.36 | 9.51±3.08 | 905.78 | 9.51±3.08 | 905.78 |
| Cropland | R18 | 2.410 | – | – | – | – | – | – | 4.00±1.41 | 96.52 | 9.51±3.08 | 229.24 | 9.51±3.08 | 229.24 |
| Wetland | R1 | 2.140 | 0.42±0.18 | 9.01 | 0.45±0.32 | 9.71 | 0.88±0.37 | 18.79 | 12.75±3.11 | 272.57 | 22.59±16.98 | 483.43 | 23.47±16.98 | 502.21 |
| Wetland | R2 | 2.260 | 0.27±0.11 | 6.20 | 0.50±0.38 | 11.38 | 0.78±0.40 | 17.58 | 9.79±5.80 | 221.24 | 21.80±18.64 | 492.64 | 22.57±18.65 | 510.23 |
| Wetland | R3 | 2.560 | 0.66±0.38 | 17.00 | 0.51±0.40 | 13.07 | 1.17±0.55 | 30.07 | 9.47±5.82 | 242.52 | 20.62±18.08 | 527.81 | 21.79±18.09 | 557.87 |
| Wetland | R4 | 1.107 | 0.17± 0.14 | 1.86 | 0.26±0.53 | 2.88 | 0.43±0.55 | 4.74 | 2.08±2.41 | 23.01 | 5.52±9.38 | 61.16 | 5.95±9.39 | 65.90 |
| Wetland | R5 | 0.124 | 0.13±0.07 | 0.17 | 0.26±0.53 | 0.32 | 0.39±0.53 | 0.49 | 2.58±2.10 | 3.19 | 6.55±4.14 | 8.09 | 6.94±4.17 | 8.57 |
| Wetland | R6 | 0.287 | 0.13±0.07 | 0.39 | 0.26±0.53 | 0.75 | 0.40±0.54 | 1.14 | 2.66±2.19 | 7.65 | 6.67±4.10 | 19.17 | 7.07±4.14 | 20.30 |
| Wetland | R7 | 0.143 | 0.13±0.07 | 0.19 | 0.26±0.53 | 0.37 | 0.40±0.54 | 0.57 | 2.66±2.19 | 3.81 | 6.67±4.10 | 9.56 | 7.07±4.14 | 10.12 |
| Wetland | R8 | 0.260 | 0.38±0.42 | 1.00 | 0.21±0.41 | 0.54 | 0.59±0.59 | 1.54 | 2.09±2.04 | 5.43 | 3.91±2.90 | 10.15 | 4.50±2.96 | 11.69 |
| Wetland | R9 | 0.012 | 0.37±0.47 | 0.04 | 0.20±0.45 | 0.02 | 0.57±0.65 | 0.07 | 2.11±2.02 | 0.25 | 4.04±2.90 | 0.45 | 4.61±2.98 | 0.55 |
| Wetland | R10 | 0.235 | 0.13±0.07 | 0.32 | 0.26±0.53 | 0.61 | 0.40±0.54 | 0.93 | 2.66±2.19 | 6.27 | 6.67±4.10 | 15.71 | 7.07±4.14 | 16.64 |
| Wetland | R11 | 1.019 | 0.14±0.04 | 1.45 | 2.10±2.07 | 21.41 | 2.24±2.07 | 22.86 | 9.09±3.75 | 92.68 | 41.45±10.71 | 422.40 | 43.69±10.91 | 445.26 |
| Wetland | R12 | 1.498 | 0.11±0.15 | 1.59 | 2.18±2.01 | 32.64 | 2.28±2.02 | 34.23 | 3.94±2.42 | 59.01 | 39.92±12.78 | 597.99 | 42.21±12.94 | 632.21 |
| Wetland | R13 | 2.371 | 0.12±0.11 | 2.78 | 2.47±1.89 | 58.49 | 2.58±1.90 | 61.27 | 8.94±4.43 | 211.92 | 29.09±9.54 | 689.70 | 31.67±9.72 | 750.97 |
| Wetland | R14 | 0.236 | 1.30±0.58 | 3.08 | 0.87±0.91 | 2.05 | 2.17±1.08 | 5.13 | 3.49± 2.83 | 8.25 | 13.60±6.36 | 32.15 | 15.77±6.45 | 37.29 |
| Wetland | R15 | 0.096 | 0.18±0.11 | 0.17 | 1.58±1.37 | 1.52 | 1.76±1.38 | 1.69 | 8.93±4.36 | 8.58 | 29.09±9.54 | 27.95 | 30.85±9.64 | 29.64 |
| Wetland | R16 | 0.080 | 0.78±0.45 | 0.62 | 0.70±0.47 | 0.56 | 1.48±0.65 | 1.18 | 4.76±3.75 | 3.81 | 12.72±12.70 | 10.18 | 14.20±12.72 | 11.37 |
| Wetland | R17 | 0.028 | 1.48±0.82 | 0.41 | 0.81±0.45 | 0.22 | 2.29±0.94 | 0.63 | 3.99±1.30 | 1.11 | 12.48±12.15 | 3.46 | 14.77±12.19 | 4.09 |
| Wetland | R18 | 0.004 | 3.45±3.08 | 0.13 | 1.09±0.72 | 0.04 | 4.53±3.16 | 0.17 | 5.18±3.87 | 0.20 | 22.72±18.90 | 0.86 | 27.25±19.16 | 1.03 |
| Shrub | R1 | 0.099 | 0.35±0.34 | 0.34 | 0.16±0.18 | 0.16 | 0.51±0.39 | 0.50 | 1.80±1.64 | 1.78 | 6.11±4.02 | 6.04 | 6.61±4.04 | 6.55 |
| Shrub | R2 | 0.492 | 0.35±0.34 | 1.70 | 0.16±0.18 | 0.79 | 0.51±0.39 | 2.50 | 1.80±1.64 | 8.85 | 6.11±4.02 | 30.02 | 6.61±4.04 | 32.51 |
| Shrub | R3 | 0.082 | 0.35±0.34 | 0.29 | 0.16±0.18 | 0.13 | 0.51±0.39 | 0.42 | 1.80±1.64 | 1.48 | 6.11±4.02 | 5.02 | 6.61±4.04 | 5.44 |
| Shrub | R4 | 4.564 | 0.27±0.27 | 12.49 | 0.15±0.18 | 6.80 | 0.42±0.32 | 19.29 | 1.75±1.47 | 79.93 | 5.88±3.97 | 268.55 | 6.31±3.98 | 287.84 |
| Shrub | R5 | 6.004 | 0.14±0.15 | 8.17 | 0.16±0.17 | 9.77 | 0.30±0.23 | 17.94 | 1.39±0.95 | 83.50 | 6.62±7.39 | 397.33 | 6.92±7.40 | 415.27 |
| Shrub | R6 | 6.255 | 0.05±0.05 | 3.26 | 0.17±0.17 | 10.45 | 0.22±0.18 | 13.70 | 1.38±0.93 | 86.31 | 6.62±7.39 | 413.94 | 6.84±7.40 | 427.64 |
| Shrub | R7 | 0.354 | 0.05±0.05 | 0.18 | 0.16±0.17 | 0.58 | 0.22±0.17 | 0.76 | 1.38±0.93 | 4.89 | 6.62±7.39 | 23.43 | 6.83±7.40 | 24.20 |
| Shrub | R8 | 6.375 | 0.55±0.36 | 34.94 | 0.28±0.17 | 17.89 | 0.83±0.40 | 52.83 | 4.18±2.04 | 266.75 | 8.14±3.50 | 519.16 | 8.97±3.52 | 571.99 |
| Shrub | R9 | 0.063 | 0.55±0.36 | 0.35 | 0.28±0.17 | 0.18 | 0.83±0.40 | 0.52 | 4.18±2.04 | 2.64 | 8.14±3.50 | 5.14 | 8.97±3.52 | 5.66 |
| Shrub | R10 | 0.534 | 0.05±0.05 | 0.28 | 0.17±0.17 | 0.89 | 0.22±0.18 | 1.17 | 1.38±0.93 | 7.37 | 6.62±7.39 | 35.34 | 6.84±7.40 | 36.51 |
| Shrub | R11 | 4.894 | 0.26±0.15 | 12.77 | 0.30±0.19 | 14.80 | 0.56±0.24 | 27.57 | 5.10±2.36 | 249.60 | 8.14±3.50 | 398.33 | 8.70±5.92 | 425.90 |
| Shrub | R12 | 0.936 | 0.26±0.15 | 2.44 | 0.30±0.19 | 2.83 | 0.56±0.24 | 5.27 | 5.10±2.36 | 47.75 | 8.14±3.50 | 76.21 | 8.70±5.92 | 81.48 |
| Shrub | R13 | 2.006 | 0.19±0.10 | 3.82 | 0.12±0.08 | 2.50 | 0.31±0.13 | 6.31 | 5.10±2.36 | 102.31 | 8.14±3.50 | 163.28 | 8.45±5.92 | 169.59 |
| Shrub | R14 | 5.465 | 0.44±0.26 | 24.24 | 0.24±0.26 | 13.21 | 0.69±0.37 | 37.45 | 4.97±2.00 | 263.67 | 11.12±6.79 | 607.95 | 11.81±6.80 | 645.40 |
| Shrub | R15 | 8.219 | 0.19±0.10 | 15.63 | 0.12±0.08 | 10.22 | 0.31±0.13 | 25.85 | 5.43±1.83 | 446.61 | 8.14±5.91 | 668.92 | 8.45±5.92 | 694.77 |
| Shrub | R16 | 23.130 | 0.45±0.27 | 103.97 | 0.28±0.28 | 63.83 | 0.73±0.39 | 167.79 | 4.97±2.00 | 1150.28 | 10.82±6.94 | 2503.78 | 11.55±6.95 | 2671.57 |
| Shrub | R17 | 5.984 | 0.44±0.27 | 26.50 | 0.27±0.27 | 16.12 | 0.71±0.38 | 42.61 | 4.96±1.88 | 296.99 | 10.41±6.00 | 623.03 | 11.12±6.01 | 665.64 |
| Shrub | R18 | 2.232 | 0.44±0.27 | 9.88 | 0.27±0.27 | 6.01 | 0.60±0.38 | 15.90 | 5.00±1.87 | 111.55 | 10.41±6.00 | 232.42 | 11.12±6.01 | 248.32 |
| Others | R1 | 0.125 | 0.06±0.06 | 0.08 | 0.09±0.09 | 0.12 | 0.15±0.11 | 0.19 | 0.94±0.94 | 1.17 | 5.96±5.09 | 7.43 | 6.12±5.09 | 7.62 |
| Others | R2 | 1.697 | 0.06±0.06 | 1.03 | 0.09±0.09 | 1.57 | 0.15±0.11 | 2.60 | 0.94±0.94 | 15.96 | 5.96±5.09 | 101.21 | 6.12±5.09 | 103.81 |
| Others | R3 | 1.668 | 0.06±0.06 | 1.01 | 0.09±0.09 | 1.54 | 0.15±0.11 | 2.55 | 0.94±0.94 | 15.68 | 5.96±5.09 | 99.43 | 6.12±5.09 | 101.99 |
| Others | R4 | 3.981 | 0.06±0.06 | 2.51 | 0.10±0.09 | 3.86 | 0.16±0.11 | 6.37 | 0.79±0.75 | 31.53 | 2.51±1.97 | 99.81 | 2.67±1.97 | 106.17 |
| Others | R5 | 35.955 | 0.09±0.14 | 34.10 | 0.12±0.13 | 44.30 | 0.22±0.19 | 78.39 | 2.30±1.41 | 825.85 | 7.07±5.15 | 2542.11 | 7.29±5.16 | 2620.50 |
| Others | R6 | 62.307 | 0.09±0.14 | 59.08 | 0.12±0.13 | 76.76 | 0.22±0.19 | 135.85 | 2.09±1.41 | 1330.05 | 7.07±5.15 | 4405.25 | 7.29±5.16 | 4541.09 |
| Others | R7 | 17.137 | 0.09±0.14 | 16.25 | 0.12±0.13 | 21.11 | 0.22±0.19 | 37.36 | 2.09±1.41 | 357.58 | 7.07±5.15 | 1211.65 | 7.29±5.16 | 1249.02 |
| Others | R8 | 8.460 | 0.07±0.07 | 5.81 | 0.10±0.07 | 8.78 | 0.17±0.10 | 14.59 | 2.33±0.57 | 197.05 | 6.03±2.31 | 510.40 | 6.21±2.31 | 524.98 |
| Others | R9 | 0.529 | 0.07±0.07 | 0.36 | 0.10±0.07 | 0.55 | 0.17±0.10 | 0.91 | 2.33±0.57 | 12.33 | 6.03±2.31 | 31.93 | 6.21±2.31 | 32.84 |
| Others | R10 | 20.540 | 0.09±0.14 | 19.48 | 0.12±0.13 | 25.31 | 0.22±0.19 | 44.78 | 2.02±1.43 | 415.49 | 7.07±5.15 | 1452.20 | 7.29±5.16 | 1496.98 |
| Others | R11 | 5.269 | 0.09±0.14 | 5.00 | 0.12±0.13 | 6.49 | 0.22±0.19 | 11.49 | 1.38±0.62 | 72.97 | 8.43±6.53 | 444.07 | 8.65±6.53 | 455.56 |
| Others | R12 | 9.195 | 0.09±0.14 | 8.72 | 0.12±0.13 | 11.33 | 0.22±0.19 | 20.05 | 1.38±0.62 | 127.34 | 6.23±1.62 | 572.85 | 6.45±1.63 | 592.90 |
| Others | R13 | 3.479 | 0.09±0.14 | 3.30 | 0.12±0.13 | 4.29 | 0.22±0.19 | 7.59 | 1.38±0.62 | 48.18 | 6.23±1.62 | 216.76 | 6.45±1.63 | 224.34 |
| Others | R14 | 3.873 | 0.17±0.10 | 6.76 | 1.25±0.89 | 48.37 | 1.42±0.89 | 55.12 | 2.68±1.11 | 103.94 | 6.04±1.59 | 233.75 | 7.46±1.82 | 288.87 |
| Others | R15 | 4.151 | 0.09±0.14 | 3.94 | 0.12±0.13 | 5.11 | 0.22±0.19 | 9.05 | 1.38±0.62 | 57.49 | 6.23±1.62 | 258.61 | 6.45±1.63 | 267.66 |
| Others | R16 | 4.672 | 0.17±0.10 | 8.15 | 1.23±0.91 | 57.66 | 1.41±0.92 | 65.82 | 2.97±1.17 | 138.76 | 6.23±1.62 | 291.07 | 7.64±1.86 | 356.88 |
| Others | R17 | 2.016 | 0.17±0.10 | 3.52 | 1.25±0.89 | 25.18 | 1.42±0.89 | 28.70 | 2.68±1.11 | 54.11 | 6.56±2.03 | 132.25 | 7.98±2.22 | 160.95 |
| Others | R18 | 0.574 | 0.17±0.10 | 1.00 | 1.25±0.89 | 7.17 | 1.42±0.89 | 8.17 | 2.68±1.11 | 15.40 | 6.56±2.03 | 37.64 | 7.98±2.22 | 45.81 |

^†^ R1, Cold humid regions; R2, Temperate humid regions; R3, Temperate semi-humid regions; R4, Temperate semi-arid regions; R5, Temperate arid regions; R6, Warm temperate arid regions; R7, Qinghai-Tibet plateau frigid arid regions; R8, Warm temperate semi-humid regions; R9, Warm temperate humid regions; R10, Qinghai-Tibet plateau temperate arid regions; R11, Qinghai-Tibet plateau temperate semi-arid regions; R12, Qinghai-Tibet plateau subfrigid semi-arid regions; R13, Qinghai-Tibet plateau subfrigid semi-humid regions; R14, North subtropical humid regions; R15, Qinghai-Tibet plateau temperate humid and semi-humid regions; R16, Mid-subtropical humid regions; R17, South subtropical humid regions; R18, Tropical humid regions;

^‡^ Vegetation carbon storage in cropland was not calculated in this study.

**Supplementary Appx.S1**

Appx.S1 Data sources for the published and unpublished data presented in this paper

**Part 1 Published paper**

1. Ai, Z. M., Y. M. Chen, and Y. Cao (2014), Storage and allocation of carbon and nitrogen in Robinia pseudoacacia plantation at different ages in the Loess hilly region, China, *Chinese Journal of Applied Ecology*, *25*(2), 333-341. (in Chinese)
2. Aiziguli, M., Y. A. Tong, X. L. Yang, and H. Y. Ma (2012), Effect of different fertilization on soil organic carbon and its fraction in farmland, *Chinese Journal of Soil Science*, *43*(6), 1461-1466. (in Chinese)
3. Aminem, E. L., S. L. Chang, Y. Zhang, T., Y. Qiu, and P. He (2014), Altitudinal distribution rule of Picea schrenkiana forest's soil organic carbon and its influencing factors, *Acta Ecologica Sinica*, *34*(7), 1626-1634. (in Chinese)
4. An, J. (2012), Tree species and management effect on soil carbon sequestration and fertilities of plantation forests [D], *Heilongjiang: Northeast Forestry University*. (in Chinese)
5. An, S. S., F. L. Zheng, F. Zhang, S. V. Pelt, U. Hamer, and F. Makeschin (2008), Soil quality degradation processes along a deforestation chronosequence in the Ziwuling Area, China, *Catena*, *75*(3), 248-256.
6. An, T. W. (2011), The effects of land use on physic-chemical property and enzymatic activities in the soil of Nandagang wetland [D], *Hebei: Hebei University*. (in Chinese)
7. Bai, C. H. (2008), Effects of land use pattern and grazing schedule on C and N reserves in 3 grassland communities in Inner Mongolian steppe [D], *Beijing: Institute of Botany, CAS*. (in Chinese)
8. Bai, F. (2007), Forest structure, *vasclar* plant species biodiversity and carbon storage change for 43 years on the north slope of Changbai mountains nature reserve, China [D], *Beijing: Institute of Botany, CAS*. (in Chinese)
9. Bai, J., J. Wang, D. Yan, H. Gao, R. Xiao, H. Shao, and Q. Ding (2012), Spatial and temporal distributions of soil organic carbon and total nitrogen in two marsh wetlands with different flooding frequencies of the Yellow River Delta, China, *CLEAN–Soil, Air, Water*, *40*(10), 1137-1144.
10. Bai, J., R. Xiao, K. Zhang, H. Gao, B. Cui, and X. Liu (2013), Soil organic carbon as affected by land use in young and old reclaimed regions of a coastal estuary wetland, China, *Soil Use and Management*, *29*(1), 57-64.
11. Bai, X. S., Y. L. Hu, D. H. Zeng, and Z. R. Jiang (2008), Effects of farmland afforestation on ecosystem carbon stock and its distribution pattern in semi-arid region of Northwest China, *Chinese Journal of Ecology*, *27*(10), 1647-1652. (in Chinese)
12. Bao, C. S. (2010), Studies on productivity and carbon flux of Larix gmelinii forest ecosystem [D], Inner: Inner Mongolia Agricultural University. (in Chinese)
13. Bao, L. D., W. F. Xu, Z. W. Wang, J. Wang, and G. D. Hang (2012), Carbon sequestration of Stipa brrviflora desert steppe under different grazing intensities, *Journal of Inner Mongolia Agricultural University*, *33*(3), 94-99. (in Chinese)
14. Bao, X. (2013), Effects of fire disturbance on carbon storage from boral Larix gmelinii-Carex schmidtii forested wetlands ecosystem in Daxing'an mountain of China [D], *Heilongjiang: Northeast Forestry University*. (in Chinese)
15. Bi, J., and C. Wang (2011), Carbon fixation ability of forest ecosystems in Mulanweichang and its characteristics, *Journal of Northwest Forestry University*, *39*(2), 45-46. (in Chinese)
16. Bu, W. S., R. G. Zang, and Y. Ding (2014), Field observed relationships between biodiversity and ecosystem functioning during secondary succession in a tropical lowland rainforest, *Acta Oecologica*, *55*(2), 1-7.
17. Bu, X. L., H. H. Ruan, L. M. Wang, W. B. Ma, J. M. Ding, and X. N. Yu (2012), Soil organic matter in density fractions as related to vegetation changes along an altitude gradient in the Wuyi Mountains, southeastern China, *Applied Soil Ecology*, *52*(1), 42-47.
18. Cai, Q. Q., Z. H. Guo, Q. P. Hu, and G. J. Wu (2013), Vertical distributin of soil organic carbon and carbon storage under different hydrologic conditions in Zoige Alpine Kobresia meadows wetland, *Scientia Silvae Sinicae*, *49*(3), 9-16. (in Chinese)
19. Cai, S. (2012), Study on biomass of three forest types in Jingouling Forest [D], *Beijing: Beijing Forestry University*. (in Chinese)
20. Cai, T. J., G. H. Xin, Y. W. Zhang, X. X. Dai, and B. Liu (2010), Characteristic of soil organic carbon of the *Sphagnum spp*. wetland in Xiaoxing'an mountains, *Science of Soil and Water Conservation*, *8*(5), 109-113. (in Chinese)
21. Cai, X. B., Y. L. Peng, S. Z. Wei, and B. Z. Yu (2014), Variation of organic carbon and humus carbon in alpine steppe soil and functions of microorganisms therein, *Acta Pedologica Sinica*, *51*(4), 834-844. (in Chinese)
22. Cai, X. B., B. Z. Yu, Y. L. Peng, and H. M. Liu (2013), The changes of soil organic carbon and carbon management index in alpine steppe, *Acta Ecologica Sinica*, *33*(24), 7748-7755. (in Chinese)
23. Cai, Z. K. (2010), Impacts of nitrogen deposition on soil carbon content and activity of soil cellulase enzymes in the Chinese fir plantation [D], *Fujian: Fujian Agriculture and Forestry University*. (in Chinese)
24. Cao, C., D. Jiang, X. Teng, Y. Jiang, W. Liang, and Z. Cui (2008), Soil chemical and microbiological properties along a chronosequence of Caragana microphylla Lam. plantations in the Horqin sandy land of Northeast China, *Applied Soil Ecology*, *40*(1), 78-85.
25. Cao, C., S. Jiang, Z. Ying, F. Zhang, and X. Han (2011), Spatial variability of soil nutrients and microbiological properties after the establishment of leguminous shrub Caragana microphylla Lam. plantation on sand dune in the Horqin Sandy Land of Northeast China, *Ecological Engineering*, *37*(10), 1467-1475.
26. Cao, G., Y. Tang, W. Mo, Y. Wang, Y. Li, and X. Zhao (2004), Grazing intensity alters soil respiration in an alpine meadow on the Tibetan plateau, *Soil Biology & Biochemistry*, *36*(2), 237-243.
27. Cao, G. M., R. J. Long, F. W. Zhang, Y. K. Li, L. Lin, X. W. Guo, D. R. Han, and J. Li (2010), A method to estimate carbon storage potential in alpine Kobresia meadows on the Qinghai-Tibetan Plateau, *Acta Ecologica Sinica*, *30*(23), 6591-6597. (in Chinese)
28. Cao, H. (2012), Initial study on the coupling correlations between soil organic carbon and inorganic carbon on the Loess Plateau [D], *Hubei: Huazhong Agricultural University*. (in Chinese)
29. Cao, J. (2012), The research about biomass and carbon storage of 4 kinds of urban forest in Guiyang in krast area [D], *Hunan: Central South University of Forestry & Technology*. (in Chinese)
30. Cao, J., E. T. Yeh, N. M. Holden, Y. Yang, and G. Du (2013), The effects of enclosures and land-use contracts on rangeland degradation on the Qinghai-Tibetan plateau, *Journal of Arid Environments*, *97*(12), 3-8.
31. Cao, J. X. (2011), Above-and belowground carbon pools in different ages of Chinese pine and oriental arborvitae plantation forests in northern mountain areas of Beijing [D], *Beijing: Beijing Forestry University*. (in Chinese)
32. Cao, J. X., X. P. Wang, Y. Tian, Z. Y. Wen, and T. S. Zha (2012), Pattern of carbon allocation across three different stages of stand development of a Chinese pine (*Pinus tabulaeformis*) forest, *Ecological Research*, *27*(5), 883-892.
33. Cao, L. F., Q. C. Zhong, Q. Liu, L. Wang, Y. Lu, and K. Y. Wang (2014), Effects of different plant arrangement modes on soil organic carbon storage and soil respiration, *Resources and Environment in the Yangtza Basin*, *22*(5), 668-675. (in Chinese)
34. Cao, S. K., G. C. Cao, K. L. Chen, J. F. Zhu, L. Chen, and B. L. Lu (2013), Characteristics of alpine wetland soil organic carbon variations around Qinghai lake, *Soils*, *45*(3), 392-398. (in Chinese)
35. Cao, S. K., K. L. Chen, G. C. Cao, J. F. Zhu, B. L. Lu, and J. M. Wang (2014), Influence of grassland degradation on the soil carbon density of the Kobresia pygmaea meadow in the Qinghai lake basin, *Research of Soil and Water Conservation*, *21*(1), 71-75. (in Chinese)
36. Cao, S. K., K. L. Chen, G. C. Cao, J. F. Zhu, B. L. Lu, T. Zhang, and J. M. Wang (2014), Characteristics of soil carbon density distribution of the Kobresia humilis meadow in the Qinghai Lake basin, *Acta Ecologica Sinica*, *34*(2), 482-490. (in Chinese)
37. Cao, X. Y. (2013), Research on carbon sequestration ability of main afforestration tree species in central and southern Shanxi, China [D], *Beijing: Beijing Forestry University*. (in Chinese)
38. Cao, X. Y., and J. P. Li (2014), Storage and distribution of soil organic carbon in different age-group Chinese fir plantations, *Journal of Central South University of Forestry & Technology*, *34*(7), 104-107. (in Chinese)
39. Cao, X. Y., B. T. Wang, L. Chi, W. J. Wang, and S. Nie (2013), The relationship between soil nutrient and carbon density of three forest stands in Lvliang, Shanxi, *Journal of Arid Land Resources and Environment*, *27*(1), 86-90. (in Chinese)
40. Cao, Y. S., F. S. Li, S. W. Nu, Z. Yang, Y. H. Wang, S. Lu, and Y. P. Gao (2012), Studies on biomass and productivity of main forest in the eastern Inner Mongolia mountains, *Journal of Inner Mongolia Agricultural University*, *33*(3), 52-57. (in Chinese)
41. Chang, R., B. Fu, G. Liu, S. Wang, and X. Yao (2012), The effects of afforestation on soil organic and inorganic carbon: A case study of the Loess Plateau of China, *Catena*, *95*(3), 145–152.
42. Chang, R. Y. (2012), Soil carbon sequestration at multi-scales for 'Grain for Green' project in Loess Plateau, China [D], *Beijing: Research Center for Eco-Environmental Sciences, CAS*. (in Chinese)
43. Chang, S., N. Liu, X. Wang, Y. Zhang, and Y. Xie (2012), Alfalfa carbon and nitrogen sequestration patterns and effects of temperature and precipitation in three agro-pastoral ecotones of northern China, *PloS one*, *7*(11), e50544.
44. Chen, C., K. L. Hu, L. E. Zhang, and L. G. Niu (2014), Estimation and spatial distribution of soil organic carbon density in alluvial plain area, *Transactions of the Chinese Society of Agricultural Engineering*, *30*(7), 64-71. (in Chinese)
45. Chen, D., Y. Zhang, Y. Lin, W. Zhu, and S. Fu (2010), Changes in belowground carbon in *Acacia crassicarpa* and *Eucalyptus urophylla* plantations after tree girdling, *Plant & Soil*, *326*(1), 123-135.
46. Chen, F. R. (2012), Research on the influences of disturbances types on vegetation and soil in the typical steppe of Loess region [D], *Shanxi: Institute of soil and water conservation, CAS and MWR*. (in Chinese)
47. Chen, F. R., J. M. Cheng, W. Liu, R. B. Zhu, X. M. Yang, X. Y. Zhao, and J. S. Su (2013), Effects of different disturbances on diversity and biomass of communities in the typical steppe of loess region, *Acta Ecologica Sinica*, *33*(9), 2856-2866. (in Chinese)
48. Chen, F. S., Y. M. Zhang, X. F. Hu, X. Feng, W. Ren, and Y. Q. Liu (2012), The pattern of ecosystem carbon stock in steep slope wild shrubs and neighboring forest plantations in hilly red soil area, *Journal of Soil and Water Conservation*, *26*(1), 151-155. (in Chinese)
49. Chen, G. J. (2013), Distribution and source analysis of carbon and nitrogen in the wetland soil of Poyang lake [D], *Shanghai: East China Institute of Technology*. (in Chinese)
50. Chen, G. S. (2009), Soil respiration and total belowground carbon allocation along a Chinese fir chronosequence [D], *Fujian: Fujian Normal University*. (in Chinese)
51. Chen, G. S., Y. S. Yang, J. S. Xie, L. I. Ling, and R. Gao (2004), Soil biological changes for a natural forest and two plantations in subtropical China, *Pedosphere*, *14*(3), 297-304.
52. Chen, G. S., Z. J. Yang, R. Gao, J. S. Xie, J. F. Guo, Z. Q. Huang, and Y. S. Yang (2013), Carbon storage in a chronosequence of Chinese fir plantations in southern China, *Forest Ecology & Management*, *300*(4), 68-76.
53. Chen, H., Y. Q. Li, S. W. Zheng, C. L. Mu, and J. Liu (2007), Research on the correlations of shrub biomass with slope-aspect and altitude in dry valley of the upper reach of the Minjiang river, *Journal of Chengdu University (Natural Science Edition)*, *26*(1), 14-18. (in Chinese)
54. Chen, H., N. Wu, S. Yao, Y. Gao, D. Zhu, Y. Wang, W. Xiong, and X. Yuan (2009), High methane emissions from a littoral zone on the Qinghai-Tibetan Plateau, *Atmospheric Environment*, *43*(32), 4995-5000.
55. Chen, H., W. Zhang, F. Gilliam, L. Liu, J. Huang, T. Zhang, W. Wang, and J. Mo (2013), Changes in soil carbon sequestration in *Pinus massoniana* forests along an urban-to-rural gradient of southern China, *Biogeosciences*, *10*(7), 6609-6616.
56. Chen, J., J. J. Cao, Y. L. Wei, B. C. Zhang, B. W. Zhu, and Z. T. Ma (2014), Primary study on the allocation pattern of grassland biomass under soil water gradient of Bird Island in Qinghai lake, *Agricultural Research in the Arid Areas*, *32*(3), 202-208. (in Chinese)
57. Chen, J., H. Z. Huang, J. Wang, Y. P. Xiang, and L. X. Shen (2013), Study on carbon storage of traditional arbor tea ecosystem of the Bulang nationality in Jingmai mountain, *Journal of West China Forestry Science*, *42*(1), 76-80. (in Chinese)
58. Chen, L. (2013), The effects of carbon stocks and soil carbon cycle in Ginkgo agroforestry systems [D], *Jiangsu: Nanjing Forestry University*. (in Chinese)
59. Chen, L., J. Gong, B. Fu, Z. Huang, Y. Huang, and L. Gui (2007), Effect of land use conversion on soil organic carbon sequestration in the loess hilly area, loess plateau of China, *Ecological Research*, *22*(4), 641-648.
60. Chen, L., X. Zeng, N. F. Y. Tam, W. Lu, Z. Luo, X. Du, and J. Wang (2012), Comparing carbon sequestration and stand structure of monoculture and mixed mangrove plantations of *Sonneratia caseolaris* and *S. apetala* in Southern China, *Forest Ecology & Management*, *284*, 222-229.
61. Chen, L. Z. (2007), Studies on soil organic carbon under main forest vegetation types in the three-gorges reservior area [D], *Beijing: Beijing Forestry University*. (in Chinese)
62. Chen, M. H., and Q. Q. Zhang (2011), Plant biomass dynamic of mix-sowed artificial grassland of Baerluke Mountains, *Grassland and Turf*, *31*(5), 37-41. (in Chinese)
63. Chen, Q., Z. Zheng, Z. L. Feng, Y. X. Ma, L. Q. Sha, H. W. Xu, P. Y. Nong, and Z. F. Li (2014), Biomass and carbon storage of *Pinus kesiya var. langbianensis* in Puer, Yunnan, *Journal of Yunnan University*, *36*(3), 439-445. (in Chinese)
64. Chen, X. (2007), Analyzing features of leaf area index, net primary productivity and tree-ring for typical forest communities in warm temperate zone [D], *Beijing: Institute of Botany, CAS*. (in Chinese)
65. Chen, X., H. Zheng, W. Zhang, X. He, L. Li, J. Wu, D. Huang, and Y. Su (2014), Effects of land cover on soil organic carbon stock in a karst landscape with discontinuous soil distribution, *Journal of Mountain Science*, *11*(3), 774-781.
66. Chen, Y., Y. Li, T. Awada, J. Han, and Y. Luo (2012), Carbon sequestration in the total and light fraction soil organic matter along a chronosequence in grazing exclosures in a semiarid degraded sandy site in China, *Journal of Arid Land*, *4*(4), 411-419.
67. Chen, Y., Y. Li, X. Zhao, T. Awada, S. Wen, and J. Han (2012), Effects of grazing exclusion on soil properties and on ecosystem carbon and nitrogen storage in a sandy rangeland of Inner Mongolia, Northern China, *Environmental Management*, *50*(4), 622-632.
68. Chen, Y. Y. (2006), Study on dynamic soil characteristics under artificially planted *Caragana-Pearshrub*, *Journal of Zhangzhou Teachers College (Natural Science)*, *3*, 83-88. (in Chinese)
69. Chen, Z. (2007), Effects of soil organic carbon on main physical properties in the Huanghai and Bohai region [D], *Liaoning: Dalian Jiaotong University*. (in Chinese)
70. Chen, Z. (2012), Effects of regional land use change on farmland quality and soil organic carbon- a case study of Yucheng, Shandong [D], *Beijing: Institute of Geographical Sciences and Natural Resources Research, CAS*. (in Chinese)
71. Cheng, J., G. Wu, L. Zhao, Y. Li, W. Li, and J. Cheng (2011), Cumulative effects of 20-year exclusion of livestock grazing on above- and belowground biomass of typical steppe communities in arid areas of the Loess Plateau, China, *Plant Soil & Environment*, *57*(1), 40-44.
72. Cheng, J. M., J. Cheng, and Y. Gao (2014), Structural characteristics of community biomass in Robinia pseudoacacia plantations under different site conditions at Webei loess region, northwestern China, *Journal of Beijing Forestry University*, *36*(2), 15-21. (in Chinese)
73. Cheng, J. M., J. Cheng, and X. M. Yang (2011), Grassland vegetation and soil carbon sequestration in the Loess plateau, *Journal of Natural Resources*, *26*(3), 401-411. (in Chinese)
74. Cheng, J. M., Z. B. Jing, J. W. Jin, and Y. Cao (2014), Restoration and utilization mechanism of degraded grassland in the semi-arid region of Loess plateau, *Scientia Sinica Vitae*, *44*(3), 267-279. (in Chinese)
75. Cheng, P. F., J. L. Wang, X. M. Wang, C. R. Yue, T. S. Xu, F. Cheng, X. Zhou, and X. H. Wang (2011), Carbon storage and density of four main trees in Shangri-la based on plot data, *Forest Inventory and Planning*, *36*(4), 12-15. (in Chinese)
76. Cheng, T. R. (2007), Research on the forest biomass and carbon storage in Xiaolong mountains, Gansu province [D], *Beijing: Beijing Forestry University*. (in Chinese)
77. Cheng, X., S. An, S. Liu, and G. Li (2004), Micro-scale spatial heterogeneity and the loss of carbon, nitrogen and phosphorus in degraded grassland in Ordos Plateau, northwestern China, *Plant & Soil*, *259*(1), 29-37.
78. Cheng, X., Y. Yang, M. Li, X. Dou, and Q. Zhang (2013), The impact of agricultural land use changes on soil organic carbon dynamics in the Danjiangkou Reservoir area of China, *Plant and soil*, *366*(1-2), 415-424.
79. Cheng, X., M. Yu, and T. Wu (2013), Effect of forest structural change on carbon storage in a coastal *Metasequoia glyptostroboides* stand, *The Scientific World Journal*, *2013*, e830509.
80. Cheng, X. Q., H. R. Han, and F. F. Kang (2012), Biomass, carbon accumulation and its partitioning of a Pinus tabulaeformis plantation ecosystem in Shanxi province, China, *Chinese Journal of Ecology*, *31*(10), 2455-2460. (in Chinese)
81. Cheng, X. R., M. K. Yu, T. G. Wu, C. X. Zhang, and G. Q. Zeng (2012), Effect of site condition on carbon storage of Quercus acutissima plantations, *Ecology and Environmental Sciences*, *21*(10), 1674-1677. (in Chinese)
82. Cheng, Y., L. Yang, Z. Cao, E. Ci, and S. Yin (2009), Chronosequential changes of selected pedogenic properties in paddy soils as compared with non-paddy soils, *Geoderma*, *151*(1), 31-41.
83. Chi, L., B. T. Wang, X. Y. Cao, N. Wang, and W. J. Wang (2013), Carbon storage and diversity of Pinus tabulaeformis and Quercus liaotungensis forests in Taiyue mountain of Shanxi province, China, *Journal of Northeast Forestry University*, *41*(8), 32-35. (in Chinese)
84. Chi, L., B. T. Wang, X. Y. Cao, N. Wang, W. J. Wang, R. J. Wang, and H. Yang (2014), Carbon storage of Chinese pine forest ecosystem in the central Shanxi province, *Journal of Arid Land Resources and Environment*, *28*(2), 81-85. (in Chinese)
85. Chi, X. W. (2013), Research of carbon storage function and nitrogen storage function of metasequoia glyptostroboides and Japanese cedar in Huaxi rainy area [D], *Sichuan: Sichuan Agricultural University*. (in Chinese)
86. Ci, E., and L. Yang (2013), Paddy soils continuously cultivated for hundreds to thousands of years still sequester carbon, *Acta Agriculturae Scandinavica, Section B-Soil & Plant Science*, *63*(8), 694-703.
87. Cui, H. X., W. F. Xiao, Z. L. Huang, L. X. Zeng, L. Pan, and H. D. Pang (2014), Soil organic carbon storage of three coniferous forests in Shennongjia nature reserve, *Journal of Northeast Forestry University*, *42*(3), 69-72. (in Chinese)
88. Cui, H. X., W. F. Xiao, L. Pan, Z. L. Huang, X. R. Wang, and H. D. Pang (2012), Characteristics of soil carbon storage of Abies fargesii forest in Shennongjia, *Scientia Silvae Sinicae*, *48*(11), 107-111. (in Chinese)
89. Cui, J. (2013), Soil carbon sequestration characteristics of oak forests and influencing factors in Shaanxi province [D], *Shanxi: Northwest Agriculture and Forestry University*. (in Chinese)
90. Cui, W., C. C. Mu, H. C. Lu, X. Bao, and B. Wang (2013), Effects of draining for forestation on carbon storage of wetland ecosystem in Daxing'an mountains of northeastern China, *Journal of Beijing Forestry University*, *35*(5), 28-36. (in Chinese)
91. Cui, X., Y. Wang, H. Niu, J. Wu, S. Wang, E. Schnug, J. Rogasik, J. Fleckenstein, and Y. Tang (2005), Effect of long-term grazing on soil organic carbon content in semiarid steppes in Inner Mongolia, *Ecological Research*, *20*(5), 519-527.
92. Cui, Y. (2004), Characteristic study of soil organic carbon in the process of sandy desertification [D], *Beijing: Chinese Agriculture University*. (in Chinese)
93. Dang, Y. A. (2008), Characteristics of the cool of soil organic carbon and soil organic nitrogen on the Loess Plateau from south to north [D], *Shanxi: Northwest Agriculture and Forestry University*. (in Chinese)
94. Dang, Y. A., S. Q. Li, G. D. Wang, and M. A. Shao (2007), Distribution characteristics of soil organic carbon and microbial biomass carbon on the Loess Plateau, *Journal of Natural Resources*, *22*(6), 936-945. (in Chinese)
95. de Blécourt, M., R. Brumme, J. Xu, M. D. Corre, and E. Veldkamp (2013), Soil carbon stocks decrease following conversion of secondary forests to rubber (*Hevea brasiliensis*) plantations, *PloS One*, *8*(7), e69357.
96. de Blécourt, M., V. M. Hänsel, R. Brumme, M. D. Corre, and E. Veldkamp (2014). Soil redistribution by terracing alleviates soil organic carbon losses caused by forest conversion to rubber plantation. *Forest Ecology and Management*, 313, 26-33.
97. Deng, J., and Z. P. Shangguan (2009), Nutrient and carbon pools in both natural and artificial Pinus tabulaeform is in Ziwuling region, *Acta Ecologica Sinica*, *29*(6), 3231-3240. (in Chinese)
98. Deng, L., Z. Shangguan, and S. Sweeney (2013), Changes in soil carbon and nitrogen following land abandonment of farmland on the Loess Plateau, China, *PloS one*, *8*(8), e71923.
99. Deng, L., S. Sweeney, and Z. P. Shangguan (2013), Grassland responses to grazing disturbance: plant diversity changes with grazing intensity in a desert steppe, *Grass & Forage Science*, *69*(69), 524–533.
100. Deng, L., K. B. Wang, M. L. Chen, Z. P. Shangguan, and S. Sweeney (2013), Soil organic carbon storage capacity positively related to forest succession on the Loess Plateau, China, *Catena*, *110*(11), 1-7.
101. Deng, L., K. B. Wang, J. P. Li, Z. P. Shangguan, and S. Sweeney (2014), Carbon storage dynamics in alfalfa (Medicago sativa) fields in the hilly-gully region of the Loess Plateau, China, *CLEAN–Soil, Air, Water*, *42*(9), 1253-1262.
102. Deng, L., Z. Zhang, and Z. Shangguan (2014), Long-term fencing effects on plant diversity and soil properties in China, *Soil and Tillage Research*, *137*, 7-15.
103. Di, Y. B., H. M. Wang, Z. Q. Ma, F. T. Yang, W. J. Zhang, X. L. Fu, Y. L. Liang, and H. Zhou (2012), Carbon storage and its allocation pattern of forest ecosystems with different restoration methods in subtropical China, *Chinese Science Bulletin*, *57*(17), 1553-1561. (in Chinese)
104. Ding, F. (2011), Effects of cultivation on soil organic carbon and total nitrogen in meadow grasslands of northeast China [D], *Liaoning: Institute of Applied Ecology, CAS*. (in Chinese)
105. Ding, F., Y. L. Hu, L. J. Li, A. Li, S. Shi, P. Y. Lian, and D. H. Zeng (2013), Changes in soil organic carbon and total nitrogen stocks after conversion of meadow to cropland in Northeast China, *Plant and soil*, *373*(1-2), 659-672.
106. Ding, F. J., Z. S. Pan, F. J. Zhou, and P. Wu (2012), Organic carbon contents and vertical distribution characteristics of the soil in three forest types of the karst regions in central Guizhou province, *Journal of Soil and Water Conservation*, *26*(1), 161-165. (in Chinese)
107. Ding, Q. Y., J. H. Bai, H. F. Gao, R. Xiao, and B. S. Cui (2009), Soil nutrient contents in yellow river delta wetlands with different plant communities, *Journal of Agro-Environment Science*, *28*(10), 2092-2097. (in Chinese)
108. Ding, X., Y. Yuan, Y. Liang, L. Li, and X. Han (2014), Impact of long-term application of manure, crop residue, and mineral fertilizer on organic carbon pools and crop yields in a Mollisol, *Journal of soils and sediments*, *14*(5), 854-859.
109. Ding, X. H., L. Gong, D. B. Wang, X. Wu, and G. H. Liu (2012), Grazing effects on eco-stoichiometry of plant and soil in Hulunbeir, Inner Mogolia, *Acta Ecologica Sinica*, *32*(15), 4722-4730. (in Chinese)
110. Ding, Y. K., J. Yang, B. Y. Song, G. Hu, and L. Zhang (2012), Effect of different vegetation types on soil organic carbon in Mu Us desert, *Acta Prataculturae Sinica*, *21*(2), 18-25. (in Chinese)
111. Ding, Y. R., S. Y. Ye, and S. Q. Zhao (2012), Nutrients and carbon sequestration in the newly created wetlands of Yellow river delta, *Geological Review*, *58*(1), 183-189. (in Chinese)
112. Dong, H. F., J. B. Yu, and B. Guan (2013), Distribution characteristics of soil organic carbon and its composition in Suaeda salsa wetland in the Yellow river delta, *Chinese Journal of Environmental Science*, *34*(1), 288-292. (in Chinese)
113. Dong, H. F., J. B. Yu, Z. G. Sun, X. J. Mu, X. B. Chen, P. L. Mao, C. F. Wu, and B. Guan (2010), Spatial distribution characteristics of organic carbon in the soil-plant systems in the Yellow river estuary tidal flat wetland, *Chinese Journal of Environmental Science*, *31*(6), 1594-1599. (in Chinese)
114. Dong, J. X. (2012), Study on the carbon storage of *Pinus taiwanensis* and the factors of influence of it in Daiyun mountain [D], *Fujian: Fujian Agriculture and Forestry University*. (in Chinese)
115. Dong, K. K., H. Wang, L. Y. Yang, B. S. Yang, and F. J. Xie (2011), Change characteristics of soil carbon and nitrogen contents in the Yellow River Delta soil after artificial restoration, *Acta Ecologica Sinica*, *31*(16), 4778-4782. (in Chinese)
116. Dong, L. L. (2012), Effect of sediment laden Yellow river irrigation on soil carbon sequestration in Ningxia, China [D], *Jiangsu: Nanjing Normal University*. (in Chinese)
117. Dong, Q. M., X. Q. Zhao, Y. S. Ma, J. J. Shi, Y. L. Wang, S. X. Li, S. H. Yang, L. Y. Wang, and L. Sheng (2012), Influence of grazing on biomass, growth ratio and compensatory effect of different plant groups in Kobresia parva meadow, *Acta Ecologica Sinica*, *32*(9), 2640-2650. (in Chinese)
118. Dong, S., L. Wen, Y. Li, X. Wang, L. Zhu, and X. Li (2012), Soil quality effects of grassland degradation and restoration on the Qinghai-Tibetan Plateau, *Soil Science Society of America Journal*, *76*(6), 2256-2264.
119. Dong, X. Y. (2009), The effect of grazing and fencing on ecological stoichiometry of carbon, nitrogen, phosporus and their storages in steppe grasslands of Loess Plateau [D], *Gansu: Lanzhou University*. (in Chinese)
120. Dong, X. Y., H. Fu, X. D. Li, D. C. Niu, D. Guo, and X. D. Li (2010), Effects on plant biomass and CNP contents of plants in grazed and fenced steppe grasslands of the Loess Plateau, *Acta Prataculturae Sinica*, *19*(2), 175-182. (in Chinese)
121. Dong, Y. X. (2011), Study on variation of carbon and nitrogen components of soil in Napahai wetland reserve [D], *Yunnan: Yunnan University*. (in Chinese)
122. Dong, Y. Z., Y. L. Wang, J. J. Zhang, Q. Zhang, and Z. P. Yang (2014), Soil carbon and nitrogen storage of different land use types in northwestern Shanxi Loess Plateau, *Chinese Journal of Applied Ecology*, *25*(4), 955-960. (in Chinese)
123. Dou, J., J. Liu, Y. Wang, and G. Zhao (2010), Experimental soil-warming effects on carbon processes of typical meadow Calamagrostis angustifolia wetland ecosystem in the Sanjiang Plain, northeast China, *Acta Agriculturae Scandinavica Section B-Soil and Plant Science*, *60*(4), 361-368.
124. Dou, X., Q. Deng, M. Li, W. Wang, Q. Zhang, and X. Cheng (2013), Reforestation of *Pinus massoniana* alters soil organic carbon and nitrogen dynamics in eroded soil in south China, *Ecological Engineering*, *52*(2), 154-160.
125. Du, F., Z. S. Liang, X. X. Xu, L. Shan, and X. C. Zhang (2007), The community biomass of abandoned farmland and its effects on soil nutrition in the loess hilly region of northern Shaanxi, China, *Acta Ecologica Sinica*, *27*(5), 1673-1683. (in Chinese)
126. Du, G. H. (2009), Study on soil physiochemical properties and assessment of soil environmental quality in wetlands of Dongting lake [D], *Beijing: Beijing Forestry University*. (in Chinese)
127. Du, G. H., S. Y. Li, J. M. Zheng, L. Y. Wang, and Y. K. Zheng (2009), Study on spatial distribution and correlation of soil organic matter in wetland of Dongting lake floodplain, *Modern Agricultural Sciences*, *16*(2), 21-23, 32. (in Chinese)
128. Du, H., T. Q. Song, F. P. Zeng, X. G. Wen, and W. X. Peng (2013), Biomass and its allocation in Pinus massoniana plantation at different stand ages in East Guangxi, *Acta Botanica Boreali-Occidentalia Sinica*, *33*(2), 394-400. (in Chinese)
129. Du, H. Y. (2012), Study on the soil physical and chemical properties in the process of oasification in Alar irrigation area [D], *Xinjiang: Xinjiang Institute of Ecology and Geography, CAS*. (in Chinese)
130. Du, M. Y. (2010), The carbon pool in different types of Phyllostachys edulis stands [D], *Beijing: Chinese Academy of Forestry*. (in Chinese)
131. Du, Y. X., Z. X. Song, C. L. He, S. X. Zhou, and Q. Huang (2013), Organic carbon content and influencing factors of different forest soil in Jiujiang, Jiangxi province, *Chinese Journal of Soil Science*, *44*(3), 575-579. (in Chinese)
132. Du, Y. X., C. J. Wu, S. X. Zhou, L. Huang, S. M. Han, X. F. Xu, and Y. Ding (2011), Forest soil organic carbon density and its distribution characteristics along an altitudinal gradient in Lushan mountains of China, *Chinese Journal of Applied Ecology*, *22*(7), 1675-1681. (in Chinese)
133. Dun, M. (2012), Study on distribution and influence factors of soil carbon and phssphorus in Yellow river estuary wetland [D], *Shandong: Ocean University of China*. (in Chinese)
134. Fan, K., Y. T. He, W. Sun, X. Z. Zhang, and L. Y. Yang (2013), Different management on soil microbial biomass carbon in the Tibetan plateau farmland, *Soil and Fertilizer Sciences in China*, *1*, 20-24. (in Chinese)
135. Fan, Q. F., D. R. Xiao, K. Tian, Q. Yao, S. B. An, and H. Z. Yu (2014), Effect of grazing on carbon and nitrogen reserve of typical plateau wetland in northwestern Yunnan, *Chinese Journal of Soil Science*, *45*(5), 1151-1156. (in Chinese)
136. Fan, Y. G. (2008), Study on organic carbon storage and the relation with environment factors in Bayanbulak [D], *Xinjiang: Xinjiang Institute of Ecology and Geography, CAS*. (in Chinese)
137. Fan, Y. G., Y. K. Hu, K. H. Li, J. M. Yu, and X. Wang (2008), Effects of different disturbances on the diversity and biomass of the Phytobiocoenoses in alpine steppes, *Arid Zone Research*, *25*(4), 531-536. (in Chinese)
138. Fan, Y. M., H. Q. Wu, G. L. Jin, W. Liu, and Adelieti (2012), Change of plant community character and soil nutrients under different utilization modes of spring-autumn steppe, *Xinjiang Agricultural Sciences*, *49*(8), 1503-1508. (in Chinese)
139. Fan, Y. X. (2011), The soil carbon pool and soil respiration during natural succession of mid-substropical evergreen broadleaved forest [D], *Fujian: Fujian Normal University*. (in Chinese)
140. Fang, H., S. Cheng, X. Zhang, A. Liang, X. Yang, and C. Drury (2006), Impact of soil redistribution in a sloping landscape on carbon sequestration in Northeast China, *Land Degradation & Development*, *17*(1), 89-96.
141. Fang, H. J., X. M. Yang, X. P. Zhang, and A. Z. Liang (2005), Distribution character of organic carbon in black soil of sloping field and calculation of loss amount of soil carbon, *China Environmental Science*, *25*, 81-84. (in Chinese)
142. Fang, H. Y. (2013), Impacts of nitrogen deposition on carbon pool and carbon sequestration benefits in the Chinese fir plantation [D], *Jiangxi: Jiangxi Agricultural University*. (in Chinese)
143. Fang, J. P. (2012), Study on biomass and productivity of Picea likiangensis var. linzhiensis forest in Nanyigou of Tibet, *Forest Research*, *25*(5), 582-589. (in Chinese)
144. Fang, J. P., and W. H. Xiang (2008), Biomass and its distribution of a primaeval Abies georgei var. smithii forest in Sejila mountain in Tibet Plateau, *Scientia Silvae Sinicae*, *44*(5), 17-23. (in Chinese)
145. Fang, K., N. P. Song, L. Wei, and H. An (2012), The effect of different grazing systems on aboveground biomass and interspecific relationships in desert steppe, *Acta Prataculturae Sinica*, *21*(5), 12-22. (in Chinese)
146. Fang, X., Z. Xue, B. Li, and S. An (2012), Soil organic carbon distribution in relation to land use and its storage in a small watershed of the Loess Plateau, China, *Catena*, *88*(1), 6-13.
147. Fang, Y. T., J. M. Mo, B. Sandra, G. Y. Zhou, Q. M. Zhang, and D. J. Li (2004), Storage and distribution of soil organic carbon in Dinghushan Biosphere Reserve, *Acta Ecologica Sinica*, *24*(1), 135-142. (in Chinese)
148. Feng, L., W. Hong, C. Z. Wu, and P. Song (2008), Organic carbon distribution in the soil-plant systems in Minjiang estuary wetland, *Journal of Fujian College of Forestry*, *28*(1), 9-13. (in Chinese)
149. Feng, X. H. (2014), Research on the distribution of organic carbon and nitrogen of soils in Xiaobohu wetlands of Qinghai lake [D], *Qinghai: Qinghai Normal University*. (in Chinese)
150. Feng, X. H., K. L. Chen, and Y. H. Mao (2014), Research on the distribution of organic carbon and nitrogen of different plant communities soils in Xiaobohu wetlands of Qinghai lake, *Journal of Anhui Agricultural Sciences*, *42*(15), 4736-4739. (in Chinese)
151. Fu, H., Y. M. Chen, Y. R. Wang, and C. G. Wan (2004), Organic carbon content in major grassland types in Alex, Inner Mongolia, *Acta Ecologica Sinica*, *24*(3), 469-476. (in Chinese)
152. Fu, H., S. F., Pei, Y. M., Chen, and C. G., Wan (2007), Influence of shrubs on soil chemical properties in Alxa desert steppe, China, *Department of Agriculture, Forest Service, Rocky Mountain Research Station*, *47*, 117-122.
153. Fu, W., P. Jiang, K. Zhao, G. Zhou, Y. Li, J. Wu, and H. Du (2014), The carbon storage in moso bamboo plantation and its spatial variation in Anji County of southeastern China, *Journal of Soils and Sediments*, *14*(2), 320-329.
154. Fu, X., M. Shao, X. Wei, and R. Horton (2010), Soil organic carbon and total nitrogen as affected by vegetation types in Northern Loess Plateau of China, *Geoderma*, *155*(1), 31-35.
155. Ganjurjav, Y. Q. Guo, Q. Z. Gao, M. J. Duan, Y. F. Wan, Y. E. Li, and L. B. Danjiu (2013), A study on optimal grazing rates in *Stipa purpurea* alpine grassland in Northern Tibet, *Acta Prataculturae Sinica*, *22*(1), 130-137. (in Chinese)
156. Gao, C. (2007), Study on the characteristics of soil organic matter of alpine meadow under different degradation degrees in eastern Qilian Mountains and its effect on productivity [D], *Gansu: Gansu Agricultural University*. (in Chinese)
157. Gao, H. L. (2013), Distribution of organic carbon and nitrogen in soil aggregates of woodlands in the west of the Loess Plateau [D], *Shanxi: Northwest Agriculture and Forestry University*. (in Chinese)
158. Gao, H. N., Y. Zhang, J. H. Qin, and Z. J. Wang (2014), Organic carbon distribution and enzyme activities of different degraded meadow soil in upstream of Heihe of Qilian Mountains, *Acta Agrestia Sinica*, *22*(2), 283-290. (in Chinese)
159. Gao, J. Q., G. C. Lei, L. Li, C. Lv, and M. Y. Bai (2010), The distribution characteristics of soil organic carbon in three kinds of wetland soils in Zoigê plateau, *Wetland Science*, *8*(4), 327-330. (in Chinese)
160. Gao, N. N., J. Chen, P. L. Zhang, S. J. Liu, Y. F. Xu, and T. M. Hu (2014), Effents of grazing intensity on the spatial distribution of aboveground biomass of alpine Kobresia meadow in Tibetan, *Acta Agrestia Sinica*, *22*(2), 255-260. (in Chinese)
161. Gao, Q., X. C. Yang, C. Y. Yin, and Q. Liu (2014), Estimation of biomass allocation and carbon density in alpine dwarf shrubs in Garze Zangzu autonomous prefecture of Sichuan province, China, *Chinese Journal of Plant Ecology*, *38*(4), 355-365. (in Chinese)
162. Gao, X., G. J. Ding, S. S. Zhai, M. F. Chen, and H. D. Du (2014), Spatial distribution of root biomass of Pinus massoniana plantations under different planting densities, *Journal of Central South University of Forestry & Technology*, *34*(6), 71-75. (in Chinese)
163. Gao, Y., J. Cheng, Z. Ma, Y. Zhao, and J. Su (2014), Carbon storage in biomass, litter, and soil of different plantations in a semiarid temperate region of northwest China, *Annals of Forest Science*, *71*(4), 427-435.
164. Gao, Y., P. Luo, N. Wu, H. Chen, and G. Wang (2007), Grazing intensity impacts on carbon sequestration in an alpine meadow on the Eastern Tibetan Plateau, *Research Journal of Agriculture & Biological Sciences*, *3*, 642-647.
165. Gao, Y., M. Schumann, X. Zeng, and H. Chen (2011), Changes of plant communities and soil properties due to degradation of alpine wetlands on the Qinghai-Tibetan plateau, *Journal of Environmental Protection & Ecology*, *12*(2), 788-798.
166. Gao, Y. P., F. J. Ding, M. L. Pan, F. J. Zhou, and P. Wu (2014), Carbon sequestration and distribution characteristics in natural secondary forests of Betula luminifera in west Guizhou, *Journal of Nanjing Forestry University (Natural Sciences Edition)*, *38*(4), 51-56. (in Chinese)
167. Ge, G., Y. H. Xu, L. Zhao, Z. Q. Wu, and L. Wu (2010), Spatial distribution characteristics of soil organic mattern and nitrogen in the Poyang lake wetland, *Resources and Environment in the Yangtza Basin*, *19*(6), 619-622. (in Chinese)
168. Ge, X. G., Z. L. Huang, R. M. Cheng, L. X. Zeng, W. F. Xiao, and B. W. Tan (2012), Effects of litterfall and root input on soil physical and chemical properties in Pinus massoniana plantations in three gorges reservoir area, China, *Chinese Journal of Applied Ecology*, *23*(12), 3301-3308. (in Chinese)
169. Ge, X. Z. (2011), Differences of soil organic carbon between orchard and farmland in hilly and gully region of southern Loess Plateau [D], *Shanxi: Northwest Agriculture and Forestry University*. (in Chinese)
170. Geng., L. J. (2010), Biological carbon storage of Larch forest in north region of Yanshan mountain [D], *Hebei: Agricultural University of Hebei*. (in Chinese)
171. Geng, Y., Y. Wang, K. Yang, S. Wang, H. Zeng, F. Baumann, P. Kuehn, T. Scholten, and J. He (2012), Soil respiration in Tibetan alpine grasslands: belowground biomass and soil moisture, but not soil temperature, best explain the large-scale patterns, *PloS one*, *7*(4), e34968.
172. Geng, Y. B., G. Q. Luo, G. F. Yuan, M. F. Li, W. Q. Meng, and Y. S. Dong (2008), Effects of cultivating and grazing on soil organic carbon and soil inorganic carbon in temperate semiarid grassland, *Journal of Agro-Environment Science*, *27*(6), 2518-2523. (in Chinese)
173. Geng, Y. Q., X. X. Yu, X. Y. Sun, J. Q. Chen, and Y. G. Yao (2007), Characteristics of soil fertility under pure Pinus tabulaeformis forests and shrubs in Badaling Mountain in area of Beijing, *Journal of Beijing Forestry University*, *29*(2), 50-54. (in Chinese)
174. Gong, C., S. L. Wang, Z. Q. Zeng, S. J. Deng, J. P. Chen, and K. S. Long (2011), Carbon storage and its distribution pattern of evergreen broad-leaved forest at different succession stages in mid-subtropical China, *Chinese Journal of Ecology*, *30*(9), 1935-1941. (in Chinese)
175. Gong, J., L. Chen, B. Fu, Y. Huang, Z. Huang, and H. Peng (2006), Effect of land use on soil nutrients in the loess hilly area of the Loess Plateau, China, *Land degradation & development*, *17*(5), 453-465.
176. Gong, W., T. X. Hu, J. Y. Wang, Y. B. Gong, and H. Ran (2008), Soil carbon pool and fertility under natural evergreen broad-leaved forest and its artificial regeneration forests in southern Sichuan province, *Acta Ecologica Sinica*, *28*(6), 2536-2545. (in Chinese)
177. Gong, Y., Y. Hu, F. Fang, Y. Liu, K. Li, and G. Zhang (2012), Carbon storage and vertical distribution in three shrubland communities in Gurbantünggüt Desert, Uygur Autonomous Region of Xinjiang, Northwest China, *Chinese geographical science*, *22*(5), 541-549.
178. Gu, L. P., Y. Q. Guo, S. Z. Ze, X. Yang, J. Li, W. Chen, and H. H. Ruan (2014), Characteristics of soil active organic carbon in Jatropha curcas plantations with different densities in dry-hot valley area of Yunnan province, *Journal of Northeast Forestry University*, *29*(2), 26-31. (in Chinese)
179. Gu, W. (2007), Carbon sequestration in soils of rehabilitated plantations on severely eroded lands in tropical China [D], *Guangdong: South China Botanical Garden, CAS*. (in Chinese)
180. Gu, W. R., X. H. Zhang, J. Z. Zhu, Z. J. Sun, X. Y. Mu, and X. J. Wang (2013), Impact of seasonal rest grazing on plant community quantity characteristics under differnent grazing intensities, *Xinjiang Agricultural Sciences*, *50*(6), 1145-1149. (in Chinese)
181. Guan, J. R., L. T. Yi, Y. F. Qian, D. T. He, S. Q. Yu, and Y. Shen (2012), The biomass and carbon storage in public welfare forest in Shengzhou city, *Hubei Agricultural Sciences*, *51*(20), 4556-4560. (in Chinese)
182. Guan, L., Z. Wu, C. Yang, and G. Xie (2012), Soil organic carbon pool and its influencing factors in rubber planted forest ecosystem at different ages in west Hainan province, *Agricultural Science & Technology*, *13*(10), 2163-2168.
183. Guo, B. H., S. H. Fan, M. Y. Du, G. L. Liu, and W. H. Su (2014), Effect of land use type on soil labile carbon pool and carbon management index, *Chinese Journal of Ecology*, *33*(3), 723-728. (in Chinese)
184. Guo, J. F., Y. S. Yang, G. S. Chen, J. S. Xie, and L. Peng (2006), Soil C and N pools in Chinese fir and evergreen broadleaf forests and their changes with slash burning in Mid-Subtropical China, *Pedosphere*, *16*(1), 56-63.
185. Guo, J. F., Y. S. Yang, and P. Lin (2006), Eco-hydrological function of forest floors in Schima superba and Cunninghamia lanceolata plantations, *Journal of Northeast Forestry University*, *34*(4), 49-51, 58. (in Chinese)
186. Guo, L. D., Y. Zhou, X. J. Zhong, Y. M. Li, X. H. Gan, K. H. Zhang, Z. Q. Li, Y. N. Liang, F. P. Xin, and W. Q. Zhang (2009), Evaluation of carbon sequestration function and its economic valued of the Eucalytus urophylla plantation in western pearl river basin, *Guangdong Forestry Science and Technology*, 8-13. (in Chinese)
187. Guo, Q., Z. M. Hu, X. R. Li, and S. G. Li (2013), Effects of precipitation timing on aboveground net primary productivity in Inner Mongolia temperate steppe, *Acta Ecologica Sinica*, *33*(15), 4808-4817. (in Chinese)
188. Guo, Q., X. J. Wang, and X. D. Yi (2014), Correlation of understory biomass and soil under Cunninghamia lanceolata pure forest of different age, *Journal of Northeast Forestry University*, *42*(3), 85-88, 98. (in Chinese)
189. Guo, X. H. (2013), Comparative studies on potential carbon sequestration of lakeshore plants in plateau wetland, northwestern Yunnan [D], *Yunnan: Southwest Forestry University*. (in Chinese)
190. Guo, X. L., K. Tian, X. X. Ge, and J. D. Lai (2012), Distribution of organic carbon density and carbon storage in plateau wetland soils in Napahai, *Journal of Soil and Water Conservation*, *26*(4), 159-162. (in Chinese)
191. Guo, X. Y., T. Cai, X. W. Duan, Y. J. Han, D. Huang, and L. J. Da (2013), Carbon storage and distribution pattern in main economic fruit forest ecosystems in Shanghai, east China, *Chinese Journal of Ecology*, *32*(11), 2881-2885. (in Chinese)
192. Guo, Y., L. Han, G. Li, J. Han, G. Wang, Z. Li, and B. Wilson (2012), The effects of defoliation on plant community, root biomass and nutrient allocation and soil chemical properties on semi-arid steppes in northern China, *Journal of Arid Environments*, *78*(3), 128-134.
193. Guo, Y. D., and J. P. Guo (2009), Research on community biomass and productivity of different forest ecosystems in Sandaochuan forest farm of Guandi mountain, *Journal of Shanxi Agricultural University: Natural Science Edition*, *29*(3), 233-237. (in Chinese)
194. Guo, Y. F., Y. F. Yao, F. C. Qing, and W. Qi (2013), Carbon stocks and carbon sequestration potentials in ecosystems of two afforestation species in typical watershed of Yanshan mountain, *Ecology and Environmental Sciences*, *22*(10), 1665-1670. (in Chinese)
195. Guo, Y. J., Y. Ni, and J. G. Han (2009), The influence of land use change on chemical and biological properties of steppe soils in northern China, *Arid Land Research and Management*, *23*(3), 197-212.
196. Guo, Y. Q. (2010), Soil organic carbon characteristics of Jatropha curcas plantation ecosystem in dry-hot valley region in Yunnan province, China [D], *Jiangsu: Nanjing Forestry University*. (in Chinese)
197. Guo, Z. H., L. Zhang, Y. R. Guo, W. Y. Wen, M. Cao, J. L. Guo, and Z. Y. Li (2014), Soil carbon sequestration and its relationship with soil pH in Qinglangang mangrove wetlands in Hainan island, *Scientia Silvae Sinicae*, *50*(10), 8-15. (in Chinese)
198. Ha, Q. (2012), The carbon storage of different grassland patterns in Saihanwula national nature reserve [D], *Inner: Inner Mongolia Agricultural University*. (in Chinese)
199. Hai, L. (2009), Studies on carbon sequestration of Larix gmelinii virgin forest and restoration forest after cutting [D], *Inner: Inner Mongolia Agricultural University*. (in Chinese)
200. Han, C. L. (2010), Temporal and spatial variation of soil nutrients of long-term monocultural cotton field and soil sustainable utilization in Xinjiang [D], *Xinjiang: Shihezi University*. (in Chinese)
201. Han, D. R. (2012), The carbon storage of alpine grassland in Qinghai-Tibetan plateau and its relationship to the climatic factors[D], *Qinghai: Northwest institute of plateau biology, CAS*. (in Chinese)
202. Han, F., W. Hu, J. Zheng, F. Du, and X. Zhang (2010), Estimating soil organic carbon storage and distribution in a catchment of Loess Plateau, China, *Geoderma*, *154*(3), 261-266.
203. Han, G., X. Hao, M. Zhao, M. Wang, B. H. Ellert, W. Willms, and M. Wang (2008), Effect of grazing intensity on carbon and nitrogen in soil and vegetation in a meadow steppe in Inner Mongolia, *Agriculture Ecosystems & Environment*, *125*(s 1–4), 21-32.
204. Han, J. J., J. M. Cheng, H. E. Wan, X. M. Yang, L. Meng, W. J. Fan, and X. J. Hu (2010), Carbon density of Quercus liaotungensis community in Ziwuling, *Journal of Northeast Forestry University*, *25*(5), 18-23. (in Chinese)
205. Han, J. J., Y. Q. Li, S. K. Wang, Y. Q. Luo, and J. Lian (2014), Characteristics of soil organic carbon and total nitrogen under different land use types in Naiman banner, *Journal of Arid Land Resources and Environment*, *28*(1), 37-42. (in Chinese)
206. Han, L., Y. J. Guo, J. G. Han, Y. J. Guo, and H. Tang (2010), A study on the diversity and aboveground biomass in a Leymus chinensis meadow steppe community under different cutting intensities, *Acta Prataculturae Sinica*, *19*(3), 70-75. (in Chinese)
207. Han, L., Z. Li, Y. Zeng, S. Q. An, and X. Leng (2013), Carbon, nitrogen, and phosphorous stoichiometry of herbaceous plant leaf and soil in riparian zone of Taihu lake basin, East China under effects of different land use types, *Chinese Journal of Ecology*, *32*(12), 3281-3288. (in Chinese)
208. Han, W. Y., J. M. Xu, K. Wei, R. Z. Shi, and L. F. Ma (2013), Soil carbon sequestration, plant nutrients and biological activities affected by organic farming system in tea (*Camellia sinensis* (L.) *O. Kuntze*) fields, *Soil science and plant nutrition*, *59*(5), 727-739.
209. Han, X., A. Tsunekawa, M. Tsubo, and S. Li (2010), Effects of land-cover type and topography on soil organic carbon storage on Northern Loess Plateau, China, *Acta Agriculturae Scandinavica Section B–Soil and Plant Science*, *60*(4), 326-334.
210. Han, X. H., X. G. Tong, G. H. Ynag, Y. L. Xue, and F. Z. Zhao (2012), Difference analysis of soil organic carbon pool in returning farmland to forest in Loess hilly area, *Transactions of the Chinese Society of Agricultural Engineering*, *28*(12), 223-229. (in Chinese)
211. Han, Y., L. Pei, and J. Du (2014), Remote sensing inversion of aboveground biomass over the Honghe wetland, *Remote Sensing Technology and Application*, *29*(2), 224-231. (in Chinese)
212. Hao, Y. J. (2013), Comparative study of soil quality under different vegetation conditions in the hilly and gully regions of the Loess Plateau [D], *Shanxi: Northwest Agriculture and Forestry University*. (in Chinese)
213. Hao, Y. P., T. T. Miao, and S. Q. Liu (2012), Research on the forest carbon storage in artificial poplar plantations in Yixiu district of Anhui province, *Journal of Sichuan Forestry Science and Technology*, *33*(2), 65-69. (in Chinese)
214. He, B., S. X. Huang, L. J. Zhao, Y. P. Chen, Y. Rong, and L. J. Luo (2009), Dynamic characteristics of carbon accumulation in Taiwania flousiana plantation ecosystem, *Scientia Silvae Sinicae*, *45*(9), 151-157. (in Chinese)
215. He, B., Y. H. Liu, H. G. Yu, W. M. Q, S. H. Wei, and S. W. Cai (2009), Carbon density and storage of Acacia mangium plantation ecosystem in Nanning, Guangxi, *Scientia Silvae Sinicae*, *45*(2), 6-11. (in Chinese)
216. He, H. Y., J. Q. Su, L. Huang, R. L. Jia, and X. R. Li (2011), Effects of fire on the structure of herbage synusia vegetation in desertified steppe, North China, *Acta Ecologica Sinica*, *31*(2), 364-370. (in Chinese)
217. He, N. (2012), The allocation of carbon stock and its formation mechanism of forest ecosystems in depressions between karst hills [D], *Hunan: Institute of Subtropical Agriculture, CAS*. (in Chinese)
218. He, N., Q. Chen, X. Han, G. Yu, and L. Li (2012), Warming and increased precipitation individually influence soil carbon sequestration of Inner Mongolian grasslands, China, *Agriculture Ecosystems & Environment*, *158*(1), 184–191.
219. He, N., Q. Yu, L. Wu, Y. Wang, and X. Han (2008), Carbon and nitrogen store and storage potential as affected by land-use in a Leymus chinensis grassland of northern China, *Soil Biology & Biochemistry*, *40*(12), 2952-2959.
220. He, N., Y. Zhang, J. Dai, X. Han, B. Taogetao, and G. Yu (2012), Land-use impact on soil carbon and nitrogen sequestration in typical steppe ecosystems,Inner Mongolia, *Journal of Geographical Sciences*, *22*(5), 859-873.
221. He, N., Y. Zhang, J. Dai, X. Han, and G. Yu (2012), Losses in carbon and nitrogen stocks in soil particle-size fractions along cultivation chronosequences in Inner Mongolian grasslands, *Journal of Environmental Quality*, *41*(5), 1507-1516.
222. He, N., Y. Zhang, Q. Yu, Q. Chen, Q. Pan, G. Zhang, and X. Han (2011), Grazing intensity impacts soil carbon and nitrogen storage of continental steppe, *Ecosphere*, *2*(1, article 8), 304-316.
223. He, S. F., D. M. Jiang, L. M. S. A, Z. M. Liu, and Y. M. Luo (2007), Sand-fixing effects of Caragana microphylla shrub in Keerqin sandy land, *Journal of Soil and Water Conservation*, *21*(1), 84-87. (in Chinese)
224. He, Y. H., X. P. Liu, and Z. K. Xie (2011), Effects of Reaumuria soongorica on its underlying soil properties and herb plant characteristics, *Chinese Journal of Ecology*, *30*(11), 2432-2436. (in Chinese)
225. He, Y. J., L. Qin, Z. Y. Li, M. X. Shao, X. Y. Liang, and L. Tan (2012), Carbon storage capacity of a Betula alnoides stand and a mixed *Betula alnoides* × *Castanopsis hystrix* stand in southern subtropical China: a comparison study, *Acta Ecologica Sinica*, *32*(23), 7586-7594. (in Chinese)
226. He, Y. L., G. Li, L. Y. Long, H. Li, C. M. Gong, Z. S. Liang, J. J. Hu, and Z. B. Luo (2011), Effects of different vegetation types on soil characteristics and plant biomass in the Loess Hilly region, *Journal of Northwest Forestry University*, *26*(6), 1-7. (in Chinese)
227. Hirota, M., K. Kawada, Q. Hu, T. Kato, Y. Tang, W. Mo, G. Cao, and S. Mariko (2007), Net primary productivity and spatial distribution of vegetation in an alpine wetland, Qinghai-Tibetan Plateau, *Limnology*, *8*(2), 161-170.
228. Hong, T. (2009), Study on carbon storage of Aleurites montana plantation ecosystem [D], *Fujian: Fujian Agriculture and Forestry University*. (in Chinese)
229. Hou, C. C., C. C. Song, Y. C. Li, and Y. D. Guo (2011), Seasonal dynamics of soil organic carbon and active organic carbon fractions in Calamagrostis angustifolia wetlands topsoil under different water conditions, *Chinese Journal of Environmental Science*, *32*(1), 290-297. (in Chinese)
230. Hou, R., Z. Ouyang, Y. Li, D. D. Tyler, F. Li, and G. V. Wilson (2012), Effects of tillage and residue management on soil organic carbon and total nitrogen in the North China Plain, *Soil Science Society of America Journal*, *76*(1), 230-240.
231. Hu, H. F. (2006), Dynamics of soil properties and organic carbon along the chronosequence of vegetation rehabilitation- a case study of pine plantation in the upper Minjiang river region, China [D], *Beijing: Research Center for Eco-Environmental Sciences, CAS*. (in Chinese)
232. Hu, H. F., and G. H. Liu (2013), Dynamics of soil physical-chemical properties and organic carbon content along a restoration chronosequence in Pinus tabulaeformis plantations, *Acta Ecologica Sinica*, *33*(4), 1212-1218. (in Chinese)
233. Hu, J. Y., Y. H. Xie, F. Li, and Z. Y. Hou (2014), Characteristics of Carex brevicuspis and its impact factors in Dingzidi, east Dongting lake, *Chinese Journal of Applied Ecology*, *25*(3), 745-751. (in Chinese)
234. Hu, M. J., F. F. Zou, and S. Q. Zou (2014), Ecological stoichiometry characteristics of the Cyperus malaccensis wetland soils in Min river estuary along with a salinity gradient, *Urban Environment & Urban Ecology*, *27*(4), 17-21. (in Chinese)
235. Hu, Q. W., H. Ouyang, and X. D. Liu (2006), Distribution characteristics of soil organic carbon and total nitrogen along the altitudinal belt in the northern slope of Qilian mountains, *Journal of Mountain Science*, *24*(6), 654-661. (in Chinese)
236. Hu, S. (2012), Research of microbial biomass and enzyme activities in soils under forest different succession stages [D], *Liaoning: Institute of Applied Ecology, CAS*. (in Chinese)
237. Hu, S. S. (2012), Monitoring research on the biomass of typical of main forest types in Xinjiang [D], *Xinjiang: Xinjiang Agricultral University*. (in Chinese)
238. Hu, W., G. Ge, Y. Xiong, and L. Wu (2012), Temporal and Spatial Patterns of Soil Nutrients in the Wetland of Nanjishan (Poyang lake), China, *Journal of Agro-Environment Science*, *31*(9), 1785-1790. (in Chinese)
239. Hu, Y., L. Wang, Y. Tang, Y. Li, J. Chen, X. Xi, Y. Zhang, X. Fu, J. Wu, and Y. Sun (2014), Variability in soil microbial community and activity between coastal and riparian wetlands in the Yangtze River estuary-Potential impacts on carbon sequestration, *Soil Biology and Biochemistry*, *70*, 221-228.
240. Hu, Y. L., D. H. Zeng, S. X. Chang, and R. Mao (2013), Dynamics of soil and root C stocks following afforestation of croplands with poplars in a semi-arid region in northeast China, *Plant & Soil*, *368*(1-2), 619-627.
241. Hu, Y. L., D. H. Zeng, Z. P. Fan, G. S. Chen, Q. Zhao, and D. Pepper (2008), Changes in ecosystem carbon stocks following grassland afforestation of semiarid sandy soil in the southeastern Keerqin Sandy Lands, China, *Journal of Arid Environments*, *72*(12), 2193-2200.
242. Hua, J. (2009), Characteristic of soil carbon pool and carbon sequestration mechanism during vegetation restoration in southern mountainous areas of Ningxia [D], *Shanxi: Institute of soil and water conservation, CAS and MWR*. (in Chinese)
243. Huang, B., W. Sun, Y. Zhao, J. Zhu, R. Yang, Z. Zou, F. Ding, and J. Su (2007), Temporal and spatial variability of soil organic matter and total nitrogen in an agricultural ecosystem as affected by farming practices, *Geoderma*, *139*(3), 336-345.
244. Huang, C. B., F. J. Zeng, J. Q. Lei, Z. Liu, and G. X. An (2011), Effect of cultivation on soil organic carbon and total nitrogen accumulation in Cele oasis croplands and their relation to crop yield, *Acta Ecologica Sinica*, *31*(18), 5113-5120. (in Chinese)
245. Huang, D. Q. (2011), Biomass and carbon storage estimation of Larix gmelinii plantation in different forest age [D], *Heilongjiang: Northeast Forestry University*. (in Chinese)
246. Huang, D. Q., L. Yu, Y. S. Zhang, and X. Q. Zhao (2011), Above-ground biomass and its relationship to soil moisture of natural grassland in the Northern slopes of the Qilian Mountains, *Acta Prataculturae Sinica*, *20*(3), 20-27. (in Chinese)
247. Huang, D. Q., L. Yu, Y. S. Zhang, and X. Q. Zhao (2011), Belowground biomass and its relationship to environmental factors of natural grassland on the Northern slopes of the Qilian Mountains, *Acta Prataculturae Sinica*, *20*(5), 1-10. (in Chinese)
248. Huang, G., X. Y., Zhao, Y. Q., Li, and J. Y., Cui (2012), Restoration of shrub communities elevates organic carbon in arid soils of northwestern China, *Soil Biology and Biochemistry*, *47*, 123-132.
249. Huang, L. (2007), Preliminary study on the carbon storage under the different kinds of plantations and the influence of environment monitoring of different vegetation in the Loess Plateau and Qinling area [D], *Shanxi: Northwest Agriculture and Forestry University*. (in Chinese)
250. Huang, L. B., J. H. Bai, H. F. Gao, X. Rong, P. P. Liu, and B. Chen (2007), Soil organic carbon content and storage of raised field wetlands in different functional zones of a typical shallow freshwater lake, China, *Soil Research, 50*(8): 664-671.
251. Huang, Q. F. (2011), Structure and biomass of dominant tree of natural secondary broad-leaved forest in the low mountain of southern Anhui [D], *Anhui: Anhui Agricultural University*. (in Chinese)
252. Huang, R., H. Wang, W. W. Ma, D. Y. Li, and X. W. Shi (2014), Soil physicochemical properties characteristics during degradation process of the flooding wetland in Gahai, *Journal of Soil and Water Conservation*, *28*(5), 221-227. (in Chinese)
253. Huang, R., H. Wang, H. Wang, and X. W. Shi (2014), Effects of enclosure year on soil physicochemical properties of sandy grassland, *Journal of Soil and Water Conservation*, *28*(1), 183-188. (in Chinese)
254. Huang, S. D., Q. B. Wu, K. B. Liao, D. X. Mo, and J. Qin (2011), Carbon storage and its allocation in an artificial Tsoongiodendron odorum ecosystem in southern subtropical region of China, *Chinese Journal of Ecology*, *30*(11), 2400-2404. (in Chinese)
255. Huang, W. J. (2008), Study on carbon storage in Minjiang river estuary [D], *Fujian: Fujian Agriculture and Forestry University*. (in Chinese)
256. Huang, X. H., D. L. Feng, H. X. Zhu, Y. H. Geng, and D. J. Chen (2014), Composition and content of soil organic carbon in Pinus massoniana pure forest in the Three Gorges Reservoir Area of central China, *Journal of Beijing Forestry University*, *36*(2), 38-43. (in Chinese)
257. Huang, X. L., Y. L. H., G. C. Lu, and L. Xue (2013), Dynamics of soil carbon density in three plantation types, *Hunan Forestry Science and Technology*, *40*(3), 18-22. (in Chinese)
258. Huang, X. X., M. Gao, W. C. F. Wei, X. D. T. Xie, and G. X. Pan (2006), Tillage effect on organic carbon in a purple paddy soil, *Pedosphere*, *16*(5), 660-667.
259. Huang, Y. (2009), Research on the effect of wetland degradation on the accumulation of organic carbon and total N in Napahai, *Journal of Anhui Agricultural Sciences*, *37*(13), 6095-6097. (in Chinese)
260. Huang, Y., S. L. Wang, Z. W. Feng, Z. Y. Ouyang, X. K. Wang, and Z. Z. Feng (2004), Changes in soil quality due to introduction of broad-leaf trees into clear-felled Chinese fir forest in the mid-subtropics of China, *Soil Use & Management*, *20*(4), 418-425.
261. Huang, Y. Z. (2009), Impacts of simlated nitrogen deposition on carbon pool and its chemical mechanism in the Chinese fir plantation [D], *Fujian: Fujian Agriculture and Forestry University*. (in Chinese)
262. Hugej, L. T. (2012), Characteristics and ecological function of vegetation of wetland in the Mu Us sandy land [D], *Inner: Inner Mongolia University*. (in Chinese)
263. Huo, C. F., W. Z. You, H. D. Zhang, T. W. Yan, W. J. Wei, G. Zhao, J. S. Guo, and Z. K. Xing (2011), Biomass and net primary productivity of Quecus mongolica plantation in Binglashan mountains in Liaoning province, *Journal of Liaoning Forestry Science & Technology*, *4*, 4-6, 11. (in Chinese)
264. Huo, L., Z. Chen, Y. Zou, X. Lu, J. Guo, and X. Tang (2013), Effect of Zoige alpine wetland degradation on the density and fractions of soil organic carbon, *Ecological engineering*, *51*, 287-295.
265. Huo, L. L. (2013), The vertical distribution and stability of SOC in marsh before and after reclaimation [D], *Jilin: Northeast Institute of Geography and Agroecology, CAS*. (in Chinese)
266. Huo, L. L., Y. C. Zou, J. W. Guo, and X. G. Lv (2013), Effect of reclamation on the vertical distribution of SOC and retention of DOC, *Chinese Journal of Environmental Science*, *34*(1), 283-287. (in Chinese)
267. Iost, S., D. Landgraf, and F. Makeschin (2007), Chemical soil properties of reclaimed marsh soil from Zhejiang Province PR China, *Geoderma*, *142*(3), 245-250.
268. Ji, H. B., S. Y. Zhuang, H. X. Zhang, B. Sun, and R. Y. Gui (2013), Zonality variation of carbon storage in Phyllostachy edulis plantation ecosystems in China, *Ecology and Environmental Sciences*, *22*(1), 1-5. (in Chinese)
269. Ji, Q., H. Y. Sun, A. Taraqqi, and X. D. Wang (2014), Impact of different tillage practices on soil organic carbon and water use efficiency under continuous wheat-maize binary cropping system, *Chinese Journal of Applied Ecology*, *25*(4), 1029-1035. (in Chinese)
270. Ji, R. R., Q. Zhang, Z. P. Yang, J. J. Zhang, and L. Wang (2007), Soil fertility characteristics induced by Caragana microphylla plantation at different growing stage on Loess plateau in north-west of Shanxi province, *Journal of Shanxi Agricultural Sciences*, *35*(3), 51-54. (in Chinese)
271. Ji, S., G. Yan, D. Li, and G. Wang (2009), Plant coverage is more important than species richness in enhancing aboveground biomass in a premature grassland, northern China, *Agriculture Ecosystems & Environment*, *129*(4), 491-496.
272. Ji, X. L., L. L. Liu, Q. Li, and Z. Q. Xu (2012), Studies on biomass of Spiraea pubescens shrub in the north region of Yanshan mountain, *Hebei Journal of Forestry and Orchard Research*, *27*(2), 206-209. (in Chinese)
273. Jia, G. M., B. R. Liu, G. Wang, and B. Zhang (2010), The microbial biomass and activity in soil with shrub (*Caragana korshinskii K.*) plantation in the semi-arid loess plateau in China, *European Journal of Soil Biology*, *46*(1), 6-10.
274. Jia, R. X. (2009), Carbon, nitrogen and phosphorous contents and storages in the marsh sediments of the Minjiang river estuary [D], *Fujian: Fujian Normal University*. (in Chinese)
275. Jiang, G. Z. (2012), Study on differences characteristic of soil organic carbon of different land-use type in dry valley of the upper reaches of Minjiang river [D], *Sichuan: Sichuan Agricultural University*. (in Chinese)
276. Jiang, G. Z., Y. B. Gong, L. W. Chen, Q. Zuo, J. X. Liu, Y. J. Yue, and X. H. Zhang (2011), Stability characteristics of soil organic carbon under brushwood vegetation in dry valley of the upper reaches of Minjiang river, *Journal of Soil and Water Conservation*, *25*(5), 209-214. (in Chinese)
277. Jiang, H. M. (2006), Dynamics of soil organic carbon, fertility and water in different land uses from the semi-arid Loess Plateau, China [D], *Gansu: Lanzhou Univeristy*. (in Chinese)
278. Jiang, H. M., J. P. Jiang, Y. Jia, F. M. Li, and J. Z. Xu (2006), Soil carbon pool and effects of soil fertility in seeded alfalfa fields on the semi-arid Loess Plateau in China, *Soil Biology and Biochemistry*, *38*(8), 2350-2358.
279. Jiang, J. P., Y. C. Xiong, Y. Jia, F. M. Li, J. Z. Xu, and H. M. Jiang (2007), Soil quality dynamics under successional alfalfa field in the semi-arid Loess Plateau of northwestern China, *Arid Land Research and Management*, *21*(4), 287-303.
280. Jiang, L., N. Lin, D. X. Mo, and Y. Zhuo (2012), Study on carbon storage and allocation of Cryptomeria fortunei plantation in the low mountain of southeast Guangxi, *Journal of Anhui Agricultural Sciences*, *40*(18), 9728-9730. (in Chinese)
281. Jiang, P., C. Meng, G. Zhou, and Q. Xu (2011), Comparative study of carbon storage in different forest stands in Subtropical China, *Botanical Review*, *77*(3), 242-251.
282. Jiang, Y., Y. G. Zhang, W. J. Liang, and D. Z. Wen (2005), Profile distribution and storage of soil organic carbon in an aquic brown soil as affected by land use, *Scientia Agricultura Sinica*, *38*(3), 544-550. (in Chinese)
283. Jiao, F., Z. M. Wen, and S. S. An (2011), Changes in soil properties across a chronosequence of vegetation restoration on the Loess Plateau of China, *Catena*, *86*(2), 110–116.
284. Jin, A. L. (2012), Study on the biomass of the main trees at Bayingzhuang forest land in Hubei province [D], *Beijing: Beijing Forestry University*. (in Chinese)
285. Jin, L., C. Y. Lu, Y. Ye, and G. F. Ye (2013), Carbon storage and fixation by Kandelia candel Mangrove in Jiulongjiang Estuary, *Journal of Fujian Forestry Science and Technology*, *40*(4), 7-11. (in Chinese)
286. Jin, M., Y. Li, S. L. Wang, X. L. Zhang, and L. Lei (2012), Alpine shrubs biomass and its distribution characteristics in Qilian mountains, *Arid Land Geography*, *35*(6), 952-959. (in Chinese)
287. Jin, Y. X., B. Xu, X. C. Yang, J. Y. Li, H. L. Ma, T. Gao, and H. D. Yu (2013), Belowground biomass and features of environmental factors in the degree of grassland desertification, *Acta Prataculturae Sinica*, *22*(5), 44-51. (in Chinese)
288. Jin, Z., Y. Dong, Y. Qi, and Z. An (2010), Soil respiration and net primary productivity in perennial grass and desert shrub ecosystems at the Ordos Plateau of Inner Mongolia, China, *Journal of arid environments*, *74*(10), 1248-1256.
289. Jin, Z., Y. Dong, Y. Qi, W. Liu, and Z. An (2013), Characterizing variations in soil pariticle-size distribution along a grass-desert transition in the Ordos plateau of Inner Mongolia, China, *Land Degradation & Development*, *24*(2), 141-146.
290. Jing, Z., J. Cheng, J. Su, Y. Bai, and J. Jin (2014), Changes in plant community composition and soil properties under 3-decade grazing exclusion in semiarid grassland, *Ecological Engineering*, *64*(3), 171-178.
291. Ju, W. Z. (2010), Age effects on stand biomass and carbon storage of Larix olgensis plantation: a case study in Dongzhelenghe forestry station of Yichun city [D], *Beijing: Beijing Forestry University*. (in Chinese)
292. Kang, L. (2012), Research on Arborous layer aboveground biomass and gross productivity of the typical forest types on the southern slope of Qinling mountains [D], *Shanxi: Northwest Agriculture and Forestry University*. (in Chinese)
293. Kang, W. X., W. W. Wang, and J. N. He (2011), Impacts of soil carbon storage on different land use in wetland and grassland of Dongting lake, *Chinese Agricultural Science Bulletin*, *27*(2), 35-39. (in Chinese)
294. Ke, X. D., L. Zhang, and Z. Y. Su (2012), Variation of soil organic carbon content along altitudinal gradient in subtropical montane forest in north Guangdong, *Journal of Ecology and Rural Environment*, *28*(2), 151-156. (in Chinese)
295. Kong, D. S., and H. Zhang (2014), Evaluation on the value of carbon sequestration function in the Heihe wetland national nature reserve in Zhangye, *Wetland Science*, *12*(1), 29-34. (in Chinese)
296. Kou, T. J. (2011), Dynamic characteristics of soil organic carbon and nitrogen under different land-use patterns and their biological mechanism - case study in rainfed agrosystem in northeast China [D], *Beijing: Chinese Academy of Agricultural Sciences*. (in Chinese)
297. Lai, J. D., K. Tian, x. L. Guo, and Q. F. Fan (2014), Organic carbon and microbial biomass carbon in soil in Napahai wetlands, *Wetland Science*, *12*(1), 49-54. (in Chinese)
298. Lang, F., G. F. Ye, Y. X. Huang, W. Gao, J. Lin, S. D. Huang, and H. Jiang (2012), Carbon storage and fraction of natural Castanopsis eyrei forest in the Wuyi mountains, *Journal of Subtropical Resources and Environment*, *7*(4), 71-77. (in Chinese)
299. Lei, C. Y., and C. Y. Tian (2008), Contents of soil organic carbon in newly reclaimed field in arid desert zone, *Journal of Arid Land Resources and Environment*, *22*(6), 105-110. (in Chinese)
300. Lei, J., Z. Y. Lei, H. R. Lin, and R. H. Zhao (2014), Analysis on variation of soil organic carbon and total nitrogen content and carbon storage in the oasis cotton field of Manas river valley, *Agricultural Science & Technology*, *15*(3), 499-502. (in Chinese)
301. Lei, L., X. D. Liu, S. L. Wang, Y. Li, and X. L. Zhang (2011), Assignment rule of alpine shrubs biomass and its relationships to environmental factors in Qilian mountains, *Ecology and Environmental Sciences*, *20*(11), 1602-1607. (in Chinese)
302. Lei, M. (2012), Effects of long-term fertilization on organic carbon fractions and turnover dynamics in paddy soils [D], *Hunan: Institute of Subtropical Agriculture, CAS*. (in Chinese)
303. Li, C., Y. Li, and L. Tang (2010), Soil organic carbon stock and carbon efflux in deep soils of desert and oasis, *Environmental Earth Sciences*, *60*(3), 549-557.
304. Li, C., O. J. Sun, C. Xiao, and X. Han (2009), Differences in net primary productivity among contrasting habitats in Artemisia ordosica rangeland of northern China, *Rangeland ecology & management*, *62*(4), 345-350.
305. Li, C. H., Y. Li, L. S. Tang, and Y. Liu (2010), Layered characters of organic carbon storage and release in Salinized gray desert soil before and after reclamation, *Arid Zone Research*, *27*(3), 385-391. (in Chinese)
306. Li, C. P. (2005), Carbon dynamics of Artemisia ordosica Krasch. Shrublands under different habitat conditions in Mu Us sandland, Inner Mongolia [D], *Beijing: Institute of Botany, CAS*. (in Chinese)
307. Li, C. Y. (2013), Biomass partitioning of Lacebark pine and it's tree-ring growth in relation to climatic factors [D], *Beijing: Beijing Forestry University*. (in Chinese)
308. Li, C. Z. (2013), The research of effects of carbon source/sink based on rotation fields in Guanzhong irrigation region [D], *Shanxi: Northwest Agriculture and Forestry University*. (in Chinese)
309. Li, D. (2006), Study on carbon storage and allocation of the Monsoonal evergreen broad-leaved forests in Xishuangbanna [D], *Yunnan: Xishuangbanna Tropical Botanical Garden, CAS*. (in Chinese)
310. Li, D. F., and M. A. Shao (2014), Soil organic carbon and influencing factors in different landscapes in an arid region of northwestern China, *Catena*, *116*, 95-104.
311. Li, D. M., F. Jiao, B. Lei, and Z. Zhang (2014), Aboveground biomass production and soil moisture characteristics of different herb communities in the Loess Hilly-gully Region, *Science of Soil and Water Conservation*, *12*(1), 33-37. (in Chinese)
312. Li, F., P. J. Yu, X. J. Shen, Y. T. Song, Q. Li, H. Y. Zhang, and D. W. Zhou (2014), Community productivity and soil carbon sequestration after Melilotus of ficinalis and Medicago falcata reseeding on degraded grassland, *Pratacultural Science*, *31*(3), 361-366. (in Chinese)
313. Li, F., W. Zhao, J. Liu, and Z. Huang (2009), Degraded vegetation and wind erosion influence soil carbon, nitrogen and phosphorus accumulation in sandy grasslands, *Plant & Soil*, *317*(1-2), 79-92.
314. Li, G., G. Jiang, Y. Li, and M. Liu (2011), Biomass carbon storage and net primary production in different habitats of Hunshandake Sandland, China, *Acta Ecologica Sinica*, *31*(4), 217-224.
315. Li, G., G. Y. Zhou, Z. M. Wu, and R. Y. Liang (2012), Aboveground biomass of a naturaly-regenerated Schima superba community at Xiaokeng of the Nanling mountain, *Scientia Silvae Sinicae*, *48*(3), 143-147. (in Chinese)
316. Li, H., J. Li, Y. He, S. Li, Z. Liang, C. Peng, A. Polle, and Z. B. Luo (2013), Changes in carbon, nutrients and stoichiometric relations under different soil depths, plant tissues and ages in black locust plantations, *Acta Physiologiae Plantarum*, *35*(10), 2951-2964.
317. Li, H. B., and X. Z. Han (2014), Estimation of carbon balance under different land uses and long-term fertilizations in black soils of Northeast China, *Chinese Journal of Eco-Agriculture*, *22*(1), 16-21. (in Chinese)
318. Li, H. B., and K. Shi (2005), Study of organic carbon and water contents in soil profile of different plant processes, *Journal of Dalian Railway Institute*, *26*(1), 92-95. (in Chinese)
319. Li, H. C. (2012), Effect of long-term fertilization on soil properties and its productivity in rain-fed field in the Northern Ecotone [D], *Beijing: Chinese Academy of Agricultural Sciences*. (in Chinese)
320. Li, H. L. (2010), Carbon storage and carbon budget of Poplar-crop intercropping ecosystem in the northern Jiangsu plain agricultural areas [D], *Jiangsu: Nanjing Forestry University*. (in Chinese)
321. Li, H. L., L. B. Chen, S. Z. Fang, and Q. X. Sun (2009), Comparison of carbon storage and distribution in different polar-crop intercropping patterns, *Scientia Silvae Sinicae*, *45*(11), 9-14. (in Chinese)
322. Li, J. F., J. M. Cheng, W. Liu, and X. L. Gu (2010), Distribution of soil organic carbon and total nitrogen of grassland in Yunwu mountain of Loess plateau, *Acta Agrestia Sinica*, *18*(5), 661-668. (in Chinese)
323. Li, J. Q. (2010), Study on single tree biomass model of Betula Platyphylla and its carbon storage in Daqing mountain, Inner Mongolia [D], *Inner: Inner Mongolia Agricultural University*. (in Chinese)
324. Li, K. (2013), The distribution pattern of carbon and nitrogen in Eucalyptus plantations under 1, 3. 5 ages [D], *Guangxi: Guangxi University*. (in Chinese)
325. Li, K. H., W. L. Wang, Y. K. Hu, G. G. Gao, Y. M. Gong, and W. Yin (2008), Relationships between belowground biomass of alpine grassland and environmental factors along an altitude gradient, *Chinese Journal of Applied Ecology*, *19*(11), 2364-2368. (in Chinese)
326. Li, K. Z. (2012), Effects of different improvement treatments on carbon storage of Pinus massoniana low-benefit forest [D], *Sichuan: Sichuan Agricultural University*. (in Chinese)
327. Li, L., J. Q. Gao, G. C. Lei, C. Lv, and L. Suo (2011), Distribution patterns of soil organic carbon and total nitrogen in Zoige peat land with different ground water table, *Chinese Journal of Ecology*, *30*(11), 2449-2455. (in Chinese)
328. Li, L., W. F. Yao, F. C. Qin, and Y. F. Guo (2014), Spatial variations of organic carbon of Huanghuadianzi watershed in Chifeng, *Acta Scientiae Circumstantiae*, *34*(3), 742-748. (in Chinese)
329. Li, L. J. (2013), The study of soil carbon pool and its stability in Swan lake alpine wetland measures [D], *Xinjiang: Xinjiang Agricultral University*. (in Chinese)
330. Li, M., X. Zhang, G. Pang, and F. Han (2013), The estimation of soil organic carbon distribution and storage in a small catchment area of the Loess Plateau, *Catena*, *101*(2), 11-16.
331. Li, M. R., and G. J. Ding (2013), Study on carbon storage of main forest types in southeast part of Guizhou province, *Journal of Central South University of Forestry & Technology*, *33*(7), 119-124. (in Chinese)
332. Li, N., G. Wang, Y. Gao, and J. Wang (2011), Warming effects on plant growth, soil nutrients, microbial biomass and soil enzymes activities of two alpine meadows in Tibetan plateau, *Polish Journal of Ecology*, *59*(1), 25-35.
333. Li, N., G. Wang, Y. Yan, Y. Gao, and G. Liu (2011), Plant production, and carbon and nitrogen source pools, are strongly intensified by experimental warming in alpine ecosystems in the Qinghai-Tibet Plateau, *Soil Biology and Biochemistry*, *43*(5), 942-953.
334. Li, N., G. X. Wang, Y. Yang, Y. H. Gao, L. N. Liu, and G. S. Liu (2011), Short-term effects of temperature enhancement on community structure and biomass of alpine meadow in the Qinghai-Tibet Plateau, *Acta Ecologica Sinica*, *31*(4), 895-905. (in Chinese)
335. Li, P., Y. Xiao, Y. Yang, and C. S. Zhang (2014), Ecosystem carbon storage in poplar plantations of different stand ages in Tianjin plain, *Chinese Journal of Ecology*, *33*(3), 567-574. (in Chinese)
336. Li, Q., M. D. Ma, Y. J. Liu, C. Liu, H. X. Ding, M. C. Chen, and Y. Chen (2007), Study on soil carbon and nutrients pools of several evergreen broad-leaved forest types in northwest Sichuan, *Journal of Soil and Water Conservation*, *21*(6), 114-117. (in Chinese)
337. Li, Q., J. Yang, B. Y. Song, W. H. Ma, L. Q. Zhao, L. X. Zhang, and H. Hou (2014), The impacts of different enclosure durations on degraded Stipa grandis grassland productivity and soil carbon and nitrogen storage, *Chinese Journal of Ecology*, *33*(4), 896-901. (in Chinese)
338. Li, Q., D. W. Zhou, Y. H. Jin, M. L. Wang, Y. T. Song, and G. D. Li (2014), Effects of fencing on vegetation and soil restoration in a degraded alkaline grassland in northeast China, *Journal of Arid Land*, *6*(4), 478-487.
339. Li, Q. Y. (2008), The research on carbon storage of populus-crop intercropping system in the Huanghuaihai plain [D], *Henan: Henan Agricultural University*. (in Chinese)
340. Li, R. L., M. W. Chai, G. Y. Qiu, F. C. Shi, and K. C. Sasa (2014), Profile nutrient distribution and sedimentary characteristic in typical marshes of Sanjiang plain, *Environmental Science*, *35*(8), 2928-2936. (in Chinese)
341. Li, R. X., J. P. Hao, J. G. Min, X. L. Chen, W. Lu, and Q. W. Guan (2012), Carbon storage changes and its mechanism of different densities in Platycladus orientalis plantation, *Ecology and Environmental Sciences*, *21*(8), 1392-1397. (in Chinese)
342. Li, R. X., N. Ling, J. P. Hao, J. G. Min, X. L. Chen, and Q. W. Guan (2013), Effects of stand ages on carbon storage, fine root morphology and biomass in Platycladus orientalis plantation, *Journal of Nanjing Forestry University (Natural Sciences Edition)*, *37*(2), 21-27. (in Chinese)
343. Li, T. (2010), Response mechanism of desert plant allometric exponents and spatial patterns to a precipitation gradient [D], *Gansu: Lanzhou Univeristy*. (in Chinese)
344. Li, T. (2012), Studies on the changes of functional groups of soil organic carbon during the process of different vegetation restoration on the Hilly Region Loess Plateau [D], *Shanxi: Institute of soil and water conservation, CAS and MWR*. (in Chinese)
345. Li, T. (2013), Research on soil and vegetation carbon storage of grassland under different land use and degree in Aba Pastoral Areas [D], *Sichuan: Sichuan Agricultural University*. (in Chinese)
346. Li, T., and G. Liu (2014), Age-related changes of carbon accumulation and allocation in plants and soil of black locust forest on Loess Plateau in Ansai County, Shaanxi Province of China, *Chinese Geographical Science*, *24*(4), 414-422.
347. Li, W., H. Huang, Z. Zhang, and G. Wu (2011), Effects of grazing on the soil properties and C and N storage in relation to biomass allocation in an alpine meadow, *Journal of Soil Science & Plant Nutrition*, *11*(4), 27-39.
348. Li, W., Z. C. Zheng, T. X. Li, and Y. D. Wang (2014), Effects of returning farmland to tea on soil organic carbon pool of hilly region in the western Sichuan, *Scientia Agricultura Sinica*, *47*(8), 1642-1651. (in Chinese)
349. Li, W. J., J. H. Li, J. M. Knops, G. Wang, J. J. Jia, and Y. Y. Qin (2009), Plant communities, soil carbon, and soil nitrogen properties in a successional gradient of sub-alpine meadows on the eastern Tibetan Plateau of China, *Environmental management*, *44*(4), 755-765.
350. Li, W. J., Z. Wang, Q. F. Han, C. H. Ren, M. K. Yan, P. Zhang, Z. K. jia, and B. P. Yang (2013), Evaluation on carbon sequestration effects of artificial alfalfa pastures in the Loess Plateau area, *Acta Ecologica Sinica*, *33*(23), 7467-7477.
351. Li, W. S. (2010), Studies on structure and productivity of natural swamp forests community in Xiaoxing' an mountains, Heilongjiang, China [D], *Heilongjiang: Northeast Forestry University*. (in Chinese)
352. Li, X., Y. Wang, L. Liu, G. Luo, Y. Li, and X. Chen (2013), Effect of land use history and pattern on soil carbon storage in arid region of Central Asia, *PloS one*, *8*(7), e68372.
353. Li, X. D. (2009), Effects of land use regimes on soil carbon in the Loess Plateau, Gansu, China [D], *Gansu: Lanzhou Univeristy*. (in Chinese)
354. Li, X. D., L. Wei, Y. C. Zhang, D. Guo, X. D. Li, and H. Fu (2009), Effects of land use regimes on soil physical and chemical properties in the Longzhong part of Loess plateau, *Acta Prataculturae Sinica*, *18*(4), 103-110. (in Chinese)
355. Li, X. F. (2006), Litter, fine root, and carbon cycling in Korean pine mixed broadleaf forest [D], *Liaoning: Institute of Applied Ecology, CAS*. (in Chinese)
356. Li, X. G., F. M. Li, R. Zed, and Z. Y. Zhan (2007), Soil physical properties and their relations to organic carbon pools as affected by land use in an alpine pastureland, *Geoderma*, *139*(1), 98-105.
357. Li, X. H., D. M. Jiang, and Y. M. Luo (2010), Soil fertile islands of shrub canopy and impacts on vegetation in chronosequence of Caragana microphylla, *Journal of Liaoning Technical University (Natural Science)*, *29*(2), 336-339. (in Chinese)
358. Li, X. H., F. C. Li, J. S. Liu, M. D. Hao, and Z. H. Wang (2014), Changes of different carbon fractions caused by long-term N fertilization in dryland soil of the Loess Plateau, *Scientia Agricultura Sinica*, *47*(14), 2795-2803. (in Chinese)
359. Li, X. Y., S. Y. Zhang, H. Y. Peng, X. Hu, and Y. J. Ma (2013), Soil water and temperature dynamics in shrub-encroached grasslands and climatic implications: results from Inner Mongolia steppe ecosystem of north China, *Agricultural and forest meteorology*, *171*, 20-30.
360. Li, Y. (2010), Carbon storage of Cunninghamia lanceolata mature plantation in Shaowu, Fujian province [D], *Beijing: Chinese Academy of Forestry*. (in Chinese)
361. Li, Y., J. Brandle, T. Awada, Y. Chen, J. Han, F. Zhang, and Y. Luo (2013), Accumulation of carbon and nitrogen in the plant–soil system after afforestation of active sand dunes in China's Horqin Sandy Land, *Agriculture Ecosystems & Environment*, *177*, 75-84.
362. Li, Y., X. Deng, C. Min, Y. Lei, and Y. Xia (2013), Soil restoration potential with corridor replanting engineering in the monoculture rubber plantations of Southwest China, *Ecological Engineering*, *51*(1), 169-177.
363. Li, Y., S. Dong, L. Wen, X. Wang, and Y. Wu (2013), The effects of fencing on carbon stocks in the degraded alpine grasslands of the Qinghai-Tibetan Plateau, *Journal of Environmental Management*, *128*(20), 393-399.
364. Li, Y., S. Dong, L. Wen, X. Wang, and Y. Wu (2014), Soil carbon and nitrogen pools and their relationship to plant and soil dynamics of degraded and artificially restored grasslands of the Qinghai-Tibetan Plateau, *Geoderma*, *213*, 178-184.
365. Li, Y., L. Hou, J. J. Chen, J. Bai, and S. X. Zhang (2014), Spatial distribution of carbon density in Pine-Oak mixed forest of Qinling mountain, *Journal of Northeast Forestry University*, *42*(1), 47-50. (in Chinese)
366. Li, Y., C. Jing, W. Mao, D. Cui, X. Wang, and X. Zhao (2014), N and P resorption in a pioneer shrub (*Artemisia halodendron*) inhabiting severely desertified lands of Northern China, *Journal of Arid Land*, *6*(2), 174-185.
367. Li, Y., T. Luo, and Q. Lu (2008), Plant height as a simple predictor of the root to shoot ratio: Evidence from alpine grasslands on the Tibetan Plateau, *Journal of Vegetation Science*, *19*(2), 245-252.
368. Li, Y., M. Shao, J. Zheng, and X. Zhang (2005), Spatial-temporal changes of soil organic carbon during vegetation recovery at Ziwuling, China, *Pedosphere*, *15*(5), 601-610.
369. Li, Y., L. Wang, W. Zhang, H. Wang, X. Fu, and Y. Le (2011), The variability of soil microbial community composition of different types of tidal wetland in Chongming Dongtan and its effect on soil microbial respiration, *Ecological Engineering*, *37*(9), 1276-1282.
370. Li, Y., J. Zhang, S. X. Chang, P. Jiang, G. Zhou, S. Fu, E. Yan, J. Wu, and L. Lin (2013), Long-term intensive management effects on soil organic carbon pools and chemical composition in Moso bamboo (*Phyllostachys pubescens*) forests in subtropical China, *Forest Ecology & Management*, *303*(5), 121-130.
371. Li, Y., J. Zhang, S. X. Chang, P. Jiang, G. Zhou, Z. Shen, J. Wu, L. Lin, Z. Wang, and M. Shen (2014), Converting native shrub forests to Chinese chestnut plantations and subsequent intensive management affected soil C and N pools, *Forest Ecology & Management*, *312*(1), 161-169.
372. Li, Y., H. Zhao, X. Zhao, and T. H. Zhang (2006), Biomass energy, carbon and nitrogen stores in different habitats along a desertification gradient in the semiarid Horqin sandy land, *Arid Land Research & Management*, *20*(1), 43-60.
373. Li, Y., G. Zhou, P. Jiang, J. Wu, and L. Lin (2011), Carbon Accumulation and Carbon Forms in Tissues During the Growth of Young Bamboo (*Phyllostachy pubescens*), *Botanical Review*, *77*(3), 278-286.
374. Li, Y., X. Zhou, J. R. Brandle, T. Zhang, Y. Chen, and J. Han (2012), Temporal progress in improving carbon and nitrogen storage by grazing exclosure practice in a degraded land area of China's Horqin Sandy Grassland, *Agriculture Ecosystems & Environment*, *159*, 55-61.
375. Li, Y., Y. Zhu, J. Zhao, G. Li, H. Wang, X. Lai, and D. Yang (2014), Effects of rest grazing on organic carbon storage in stipa grandis steppe in Inner Mongolia, China, *Journal of Integrative Agriculture*, *13*(3), 624-634.
376. Li, Y. C. (2012), Effect of nitrogen availability on carbon and nitrogen dynamics in wetland ecosystems of northeast China [D], *Jilin: Northeast Institute of Geography and Agroecology Ecology, CAS*. (in Chinese)
377. Li, Y. J. (2013), Composition and distribution characteristics of soil carbon of farmland in the Loess Plateau [D], *Shanxi: Northwest Agriculture and Forestry University*. (in Chinese)
378. Li, Y. J. (2013), Effects of rest grazing on plant diversity and organic carbon storage on Stipa Baicalensis Steppe in Inner Mongolia [D], *Liaoning: Shenyang Agricultural University*. (in Chinese)
379. Li, Y. J. (2013), Response of alpine grassland to land use pattern changes on Qinghai-Tibet Plateau [D], *Gansu: Gansu Agricultural University*. (in Chinese)
380. Li, Y. J., G. M. Cao, and R. J. Long (2012), Effects of grassland using models on soil carbon, nitrogen and phosphorus content, *Grassland and Turf*, *32*(5), 26-29. (in Chinese)
381. Li, Y. L., and Z. R. Nan (2008), Effects of cultivation on nutrient contents and main properties in desert soil of northwest arid region-a case study in Linze county of Gansu province, *Journal of Arid Land Resources and Environment*, *22*(10), 147-151. (in Chinese)
382. Li, Y. L., C. L. Xiao, L. Wang, W. Q. Zhang, S. P. Zhang, H. L. Wang, X. H. Fu, and Y. Q. Le (2009), Difference and its formation cause in soil organic carbon accumulation capability of two typical tidal wetlands at Dongtan of Chongming island in Shanghai, *Chinese Journal of Applied Ecology*, *20*(6), 1310-1316. (in Chinese)
383. Li, Y. M., G. M. Cao, and R. H. Xu (2008), Effects of different land use modes on organic carbon and its component in alpine meadow soil, *Journal of Anhui Agricultural Sciences*, *36*(14), 5951-5953. (in Chinese)
384. Li, Y. N., X. F. Yu, Z. Q. Xu, L. L. Liu, W. X. Yao, and L. Wang (2014), Carbon density characteristics of two typical shrub communities in the northern mountain region of Hebei, *Scientia Silvae Sinicae*, *50*(6), 28-33. (in Chinese)
385. Li, Y. N., L. Zhao, S. X. Xu, G. R. Yu, M. Y. Du, Q. X. Wang, X. M. Sun, Y. H. Tang, X. Q. Zhao, and G. Song (2006), Plant community structure and ecological characteristics of the alpine wetland in Haibei area of Qilian Mountains, *Journal of Glaciology and Geocryology*, *28*(1), 76-84. (in Chinese)
386. Li, Y. Y., S. K. Dong, L. Zhu, L. Wen, X. Y. Li, and X. X. Wang (2013), Adaptation strategies of reproduction of plant community in response to grassland degradation and artificial restoration, *Acta Ecologica Sinica*, *33*(16), 1-9. (in Chinese)
387. Li, Y. Y., M. A. Shao, J. Y. Zheng, and Q. F. Li (2007), Impact of grassland recovery and reconstruction on soil organic carbon in the northern Loess plateau, *Acta Ecologica Sinica*, *27*(6), 2279-2287. (in Chinese)
388. Li, Y. Z., J. W. Fan, L. X. Zhang, J. Zhai, G. F. Liu, and J. Li (2013), The impact of different land use and management on community composition, species diversity and productivity in a typical temperate grassland, *Acta Prataculturae Sinica*, *22*(2), 1-9. (in Chinese)
389. Li, Z. (2013), Distribution characteristics of soil organic carbon in Mangrove wetlands in Hainan island [D], *Hainan: Hainan Normal University*. (in Chinese)
390. Li, Z. C. (2006), The effects of land use change on the soil organic carbon [D], *Beijing: Chinese Academy of Forestry*. (in Chinese)
391. Lian, P. Y., D. H. Zeng, J. Y. Liu, F. Ding, and Z. W. Wu (2012), Impact of land-use change on carbon stocks in meadow steppe of northeast China, *First International Conference on Cellular Molecular Biology, Biophysics and Bioengineering, 108*, 262-268.
392. Liang, A. Z. (2008), The potential and mechanism to restore soil organic carbon with black soil in Northeast China [D], *Jilin: Northeast Institute of Geography and Agroecology, CAS*. (in Chinese)
393. Liang, A. Z., X. P. Zhang, H. J. Fang, X. M. Yang, and C. F. Drury (2007), Short-term effects of tillage practices on organic carbon in clay loam soil of northeast China, *Pedosphere*, *17*(5), 619-623.
394. Liang, B., L. Di, C. Y. Zhao, S. Z. Peng, H. H. Peng, C. Wang, Y. Wang, and Y. Y. Liu (2013), Altitude distribution of aboveground biomass of typical shrubs in the Tianlaochi watershed of Qilian mountains, *Acta Agrestia Sinica*, *21*(4), 664-669. (in Chinese)
395. Liang, F. (2013), The effects of thinning treatment on carbon dynamic of Chinese arborvitae plantation in mountain area of Beijing [D], *Beijing: Beijing Forestry University*. (in Chinese)
396. Liang, Q., H. Chen, Y. Gong, M. Fan, H. Yang, R. Lal, and Y. Kuzyakov (2012), Effects of 15 years of manure and inorganic fertilizers on soil organic carbon fractions in a wheat-maize system in the North China Plain, *Nutrient Cycling in Agroecosystems*, *92*(1), 21-33.
397. Liang, X. F. (2008), The effects of vegetation restoration on soil physical properties in Ziwuling secondary forest region [D], *Shanxi: Institute of soil and water conservation, CAS and MWR*. (in Chinese)
398. Liao, G. X. (2013), Study on the litter decomposition and the factors in peatland [D], *Jilin: Northeast Normal University*. (in Chinese)
399. Liao, X. J., D. J. He, R. Wang, J. B. Cai, S. C. Su, Z. R. Zhang, S. H. Xiao, Z. W. Cheng, and P. Huang (2013), Distribution pattern of soil organic carbon contents in the coastal wetlands in eastern Fujian, *Wetland Science*, *11*(2), 192-197. (in Chinese)
400. Lin, B. Z. (2013), Carbon stock and SOC stability in soils of typical plantations in north China [D], *Liaoning: Liaoning University*. (in Chinese)
401. Lin, D., J. Lai, H. C. Mullerlandau, X. Mi, and K. Ma (2012), Topographic variation in aboveground biomass in a subtropical evergreen broad-leaved forest in China, *PloS One*, *7*(10), e48244.
402. Lin, F. (2008), Soil carbon content and density in soil profiles of natural wetlands and their consequent to cultivated cropland [D], *Jiangsu: Nanjing Agricultural University*. (in Chinese)
403. Lin, F., D. Y. Li, G. X. Pan, X. W. Xu, X. H. Zhang, C. D. Chi, and Z. P. Li (2008), Carbon density of soil of wetland and its change after cultivation along the Yangtze river in Anhui province, China, *Wetland Science*, *6*(2), 192-197. (in Chinese)
404. Lin, W. (2010), Study on organic carbon density of forest ecosystem in Jinggangshan [D], *jiangxi: Nanchang University*. (in Chinese)
405. Lin, W., J. Y. Li, P. Zhou, and L. Wei (2014), Spatial distribution of carbon storage of three forests ecosystem in Guangzhou, China, *Guangdong Forestry Science and Technology*, *30*(2), 1-7. (in Chinese)
406. Lin, X. F. (2014), Research of vertical distribution characteristics of Castanopsis fissa plantations' biomass, *Jiangxi Forestry Science and Technology*, *42*(1), 10-12. (in Chinese)
407. Lin, Y., M. Hong, G. Han, M. Zhao, Y. Bai, and S. X. Chang (2010), Grazing intensity affected spatial patterns of vegetation and soil fertility in a desert steppe, *Agriculture Ecosystems & Environment*, *138*(3-4), 282-292.
408. Liu, B. (2011), Wetlands soil humus compositions and carbon density distribution characteristics in Heilongjiang province [D], *Heilongjiang: Northeast Forestry University*. (in Chinese)
409. Liu, B., X. L. Man, and Y. Wang (2011), Spatial distribution characteristics of soil organic carbon and nitrogen in main wetlands in Daxing'anling, *Journal of Northeast Forestry University*, *39*(3), 89-91. (in Chinese)
410. Liu, B. R. (2010), Changes in soil microbial biomass carbon and nitrogen under typical plant communies along an altitudinal gradient in east side of Helan mountain, *Ecology and Environmental Sciences*, *19*(4), 883-888. (in Chinese)
411. Liu, C. (2012), Study on the dynamic changes of carbon nitrogen and phosphorus in the reed wetland with duck manure application and the calculation of reserves [D], *Hebei: Agricultural University of Hebei*. (in Chinese)
412. Liu, C., W. Xiang, P. Lei, X. Deng, D. Tian, X. Fang, and C. Peng (2014), Standing fine root mass and production in four Chinese subtropical forests along a succession and species diversity gradient, *Plant & Soil*, *376*(1-2), 445-459.
413. Liu, C. J., J. H. Li, X. R. Lian, N. Cao, Y. Xiao, and C. P. Zhou (2014), The carbon storage of forests and its spatial distribution pattern in Yaoxiang forest fram, *Journal of Shandong Agricultural University (Natural Science Edition)*, *45*(2), 181-186. (in Chinese)
414. Liu, C. Q., A. Li, B. Li, J. X. Wang, Y. J. Zhang, and S. Liu (2012), Dynamics of biomass, nitrogen and phosphorus storage of *Phragmites australis* in Baiyangdian Lake, *Acta Scientiae Circumstantiae*, *32*(6), 1503-1511. (in Chinese)
415. Liu, E., C. Yan, X. Mei, Y. Zhang, and T. Fan (2013), Long-term effect of manure and fertilizer on soil organic carbon pools in dryland farming in northwest China, *PloS one*, *8*(2), e56536.
416. Liu, F., K. Yang, K. Li, M. Peng, and H. F. Nie (2011), Profile distribution of organic carbon of four typical soils in China, *Earth Science Frontiers*, *18*(6), 20-26. (in Chinese)
417. Liu, F., G. L. Zhang, Y. J. Sun, Y. G. Zhao, and D. C. Li (2013), Mapping the three-dimensional distribution of soil organic matter across a subtropical hilly landscape, *Soil Science Society of America Journal*, *77*(4), 1241-1253.
418. Liu, G. (2011), The research on the landscape successions and the carbon storage of Honghu wetland in China [D], *Hunan: Central South University of Forestry & Technology*. (in Chinese)
419. Liu, G., and L. Chen (2013), Study on carbon storage in Honghu lake wetland, *Journal of Central South University of Forestry & Technology*, *33*(8), 103-107. (in Chinese)
420. Liu, G., S. Y. Shen, W. D. Yan, D. L. Tian, Q. Wu, and X. C. Liang (2011), Characteristics of organic carbon and nutrient content in five soil types in Honghu wetland ecosystems, *Acta Ecologica Sinica*, *31*(24), 7625-7631. (in Chinese)
421. Liu, G., J. Y. Zhu, Y. C. Ye, S. S. Liu, and Z. Y. Su (2010), Organic carbon storage and its distribution in forest litters among forest communities in Dongguan,south China, *Journal of Mountain Research*, *28*(1), 69-75. (in Chinese)
422. Liu, G. H. (2012), The study of the carbon gain of Eucalyptus Dunnii plantation and the relationship between vegetation and carbon gain [D], *Fujian: Fujian Agriculture and Forestry University*. (in Chinese)
423. Liu, H., J. Wang, J. Lv, and K. Wang (2010), Response of grasslands conversion to croplands on soil organic carbon in Bashang area of Northern China, *African Journal of Biotechnology*, *9*(12), 1783-1788.
424. Liu, H. Y. (2011), Dynamics change and quantification of sequestration potential for soil organic carbon in croplands in Liaoning province [D], *Liaoning: Shenyang Agricultural University*. (in Chinese)
425. Liu, J. (2011), Research on biological productivity and characteristics of soil nutrients of main tree species for shelterbelt in Haitan island [D], *Fujian: Fujian Normal University*. (in Chinese)
426. Liu, J. D. (2010), Study of rangeland degeneration base on climate herbage yield model and remote sensing herbage yield model to take Evenk Autonomous banner Inner Mongolia as example [D], *Inner: Inner Mongolia Agricultural University*. (in Chinese)
427. Liu, J. L. (2013), Study on impact of mowing and grazing on vegetation and soil of typical steppe in Xilingol, Inner Mongolia [D], *Beijing: Chinese Academy of Agricultural Sciences*. (in Chinese)
428. Liu, J. N. (2008), The forest carbon storage temporal and spatial analysis of main forest types in Huanglong mountain based on GIS [D], *Shanxi: Northwest Agriculture and Forestry University*. (in Chinese)
429. Liu, J. X., G. Y. Zhou, D. Q. Zhang, Z. H. Xu, H. L. Duan, Q. Deng, and L. Zhao (2010), Carbon dynamics in subtropical forest soil: effects of atmospheric carbon dioxide enrichment and nitrogen addition, *Journal of Soils & Sediments*, *10*(4), 730-738.
430. Liu, L., H. Y. Wang, X. J. Yang, X. Li, and L. N. Ren (2013), Soil organic carbon and nutrients in natural Larix olgensis at different stand densities, *Journal of Northeast Forestry University*, *41*(2), 51-55. (in Chinese)
431. Liu, L. L. (2006), A study on plant diversity and biomass of managed meadows in the Tibetan region, NW Yunan, China [D], *Yunnan: Xishuangbanna Tropical Botanical Garden, CAS*. (in Chinese)
432. Liu, M., B. Wang, C. P. Osborne, and G. Jiang (2013), Chicken farming in grassland increases environmental sustainability and economic efficiency, *Plos One*, *8*(1), e53977.
433. Liu, M. Y., Q. R. Chang, and X. Y. Yang (2010), Soil carbon fractions under different land use types in the tablelands of the Loess plateau, *Plant Nutrition and Fertilizer Science*, *16*(6), 1418-1425. (in Chinese)
434. Liu, N., K. L. Wang, Y. H. Xie, G. Yang, and Y. F. Duan (2011), Characteristics of the soil environment of Dongting Lake wetlands and its response to the converting farmland to lake project, *Acta Ecologica Sinica*, *31*(13), 3758-3766. (in Chinese)
435. Liu, Q. (2013), Effects of selection cutting on carbon density and net primary productivity of the mixed broadleaved-Korean pine forest [D], *Heilongjiang: Northeast Forestry University*. (in Chinese)
436. Liu, Q., H. Yin, J. Chen, C. Zhao, X. Cheng, Y. Wei, and B. Lin (2011), Belowground responses of Picea asperata seedlings to warming and nitrogen fertilization in the eastern Tibetan Plateau, *Ecological Research*, *26*(3), 637-648.
437. Liu, S. S., X. H. Zhang, Y. B. Gong, Y. Li, Y. Wang, Y. J. Yin, J. S. Ma, and T. Guo (2014), Effects of grazing disturbance on soil active organic carbon in mountain forest-arid valley ecotone in the upper reaches of Minjiang river, *Chinese Journal of Applied Ecology*, *25*(2), 359-366. (in Chinese)
438. Liu, T. (2013), Research on ecosystem carbon denstiy and its distribution characteristics under different grain for green models in hilly area of south Mingxia [D], *Shanxi: Northwest Agriculture and Forestry University*. (in Chinese)
439. Liu, T., X. H. Dang, G. B. Liu, B. J. Liu, and C. K. Shao (2013), Comparison of carbon densities of three shrub plantation ecosystems in Hilly Loess plateau, *Journal of Northwest A & F University*, *41*(9), 68-72. (in Chinese)
440. Liu, T. T. (2009), Calculationg biomass and carbon storage of poplar plantation based on tree structure [D], *Beijing: Beijing Forestry University*. (in Chinese)
441. Liu, T. Y. (2012), Carbon storage in Manchurian ash and Dahurian larch plantations with different stand structures [D], *Heilongjiang: Northeast Forestry University*. (in Chinese)
442. Liu, W. M. (2011), The research of soil organic carbon storage in natural grassland ecosystem in the Loess Plateau [D], *Shanxi: Institute of soil and water conservation, CAS and MWR*. (in Chinese)
443. Liu, W., S. Chen, Q. Zhao, Z. Sun, J. Ren, and D. Qin (2014), Variation and control of soil organic carbon and other nutrients in permafrost regions on central Qinghai-Tibetan Plateau, *Environmental Research Letters*, *9*(11), 114013.
444. Liu, W. F., J. P. Wu, H. B. Fan, Y. Y. Li, Y. H. Yuan, Y. C. Liao, R. Z. Huang, L. Hu, H. Y. Fang, and H. B. Guo (2013), Carbon pools in an age sequence of *Eucalyptus* plantation forest, *Ecology and Environmental Sciences*, *22*(1), 12-17. (in Chinese)
445. Liu, W. J., Y. J. Wang, H. Cui, J. Peng, H. J. Si, and P. J. Zhang (2014), Changes of distribution of organic carbon in soil profiles of wetlands recovered from farmlands to lakes in Caizi Lake, *Journal of Chizhou Teachers College*, *28*(3), 10-13. (in Chinese)
446. Liu, W. L., W. X. Xie, Q. S. Zhao, K. J. Zhu, and R. R. Yu (2014), Spatial distribution and ecological stoichiometry characteristics of carbon, nitrogen and phosphorus in soil in Phragmites australis tidal flat of Jiaozhou Bay, *Wetland Science*, *12*(3), 362-368. (in Chinese)
447. Liu, X. (2012), Effects of cutting disturbances on soil carbon and nitrogen content from Larix forested wetlands in Xiaoxing' an mountain [D], *Heilongjiang: Northeast Forestry University*. (in Chinese)
448. Liu, X., C. Y. Li, K. L. Jia, S. Zhang, and X. H. Shi (2013), Spatial distribution and storage characteristic of organic carbon in sediments of lake wetland in northern arid areas: a case study of Wuliangsuhai lake, *Ecology and Environmnet*, *22*(2), 319-324. (in Chinese)
449. Liu, X., W. Zhang, J. Cao, H. Shen, X. Zeng, Z. Yu, and X. Zhao (2013), Carbon storages in plantation ecosystems in sand source areas of north Beijing, China, *PloS One*, *8*(12), e82208.
450. Liu, X., W. Zhang, Z. Liu, F. Qu, and X. Tang (2011), Changes in species diversity and above-ground biomass of shrubland over long-term natural restoration process in the Taihang Mountain in North China, *Plant Soil Environ*, *57*, 505-512.
451. Liu, X. A., P. H. Peng, L. Wang, and Z. L. Yang (2013), Research on forest ecosystem servives value of different forest stands in Fenglin nature reserve *Forestry Science and Technology*, *38*(1), 31-34, 39. (in Chinese)
452. Liu, X. H. (2013), C, N, P stoichiometry of plants and soil in the wetland of yellow river delta [D], *Shandong: Shandong Agricultural University*. (in Chinese)
453. Liu, X. L., X. D. Hao, D. S. Yang, S. R. Liu, Y. M. Su, X. H. Cai, F. He, and Q. Y. Ma (2006), Aboveground biomass and its models of *Quercus aquifolioides* thicket community in Balangshan mountain in Wolong Natural Reserve, *Chinese Journal of Ecology*, *25*(5), 487-491. (in Chinese)
454. Liu, Y., X. Z. Li, Z. Z. Yan, X. Z. Chen, Y. L. He, W. Y. Guo, and P. Y. Sun (2013), Biomass and carbon storage of Phragmites australis and Spartina alterniflora in Jiuduan Shoal Wetland of Yangtze Estuary, East China, *Chinese Journal of Applied Ecology*, *24*(8), 2129-2134. (in Chinese)
455. Liu, Y., C. Liu, S. Wang, K. Guo, J. Yang, X. Zhang, and G. Li (2013), Organic carbon storage in four ecosystem types in the karst region of southwestern China, *PloS One*, *8*(2), 3692-3699.
456. Liu, Y., Y. S. Ma, S. X. Li, W. Zheng, and S. H. Yang (2014), Species diversity and biomass characteristics of different grain-for-green grasslands in the northern region of Qinghai lake, *Acta Agriculturae Boreali-occidentalis Sinica*, *23*(1), 48-52. (in Chinese)
457. Liu, Y., Y. Q. Su, L. L. Zhang, J. Wang, and X. M. Xu (2013), Study on dynamic change of organic carbon in young Robinia pseudoacacia plantation in Loess Plateau, *Journal of Nanjing Forestry University (Natural Sciences Edition)*, *37*(2), 28-32. (in Chinese)
458. Liu, Y. C. (2010), Research on the changes of aboveground biomass and NPP in recovery process for main forest types in subalpine region of western Sichuan [D], *Beijing: Chinese Academy of Forestry*. (in Chinese)
459. Liu, Y. C., Q. F. Wang, G. R. Yu, X. J. Zhu, X. Y. Zhan, Q. Guo, H. Yang, S. G. Li, and Z. M. Hu (2011), Ecosystems carbon storage and carbon sequestration potential of two main tree species for the Grain for Green Project on China's hilly Loess Plateau, *Acta Ecologica Sinica*, *31*(15), 4277-4286. (in Chinese)
460. Liu, Y. H. (2011), The characteristics of growth and carbon sequestration and water consumption in the small watershed of Xiangshuihe, Liupan montains [D], *Beijing: Chinese Academy of Forestry*. (in Chinese)
461. Liu, Y. H., X. L. Li, C. H. Li, H. Q. Sun, G. X. Lu, and G. X. Pan (2009), Vegetation decline and reduction of soil organic carbon stock in high-altitude meadow grasslands in the source area of three major rivers of China, *Journal of Agro-Environment Science*, *28*(12), 2559-2567. (in Chinese)
462. Liu, Y. H., Y. H. Wang, P. T. Yu, W. Xiong, J. Hao, X. B. Zhang, and L. H. Xu (2012), Soil organic carbon contents of Larix *principis-rupprechtii* plantations in the southern part of Liupan mountains, *Scientia Silvae Sinicae*, *48*(12), 1-9. (in Chinese)
463. Lou, X. D., S. Q. Zhai, B. Kang, and L. L. Hu (2014), Seasonal dynamic characteristics of dissolved organic carbon in Zoige peatland and its impact factors, *Research of Environmental Sciences*, *27*(2), 157-163. (in Chinese)
464. Lü, X., J. Yin, M. R. Jepsen, and J. Tang (2010), Ecosystem carbon storage and partitioning in a tropical seasonal forest in Southwestern China, *Forest Ecology & Management*, *260*(10), 1798-1803.
465. Lu, G. C., Y. Xue, L. Xue, and Y. Shao, R. (2014), Distribution characteristics of soil organic carbon in cutover land of a Cunninghamia lanceolata stand, *Journal of Anhui Agricultural University*, *41*(1), 126-129. (in Chinese)
466. Lu, H. C. (2013), Effects of harvesting on carbon storage of boreal Larix gmelinii- Carex schmidtii forested wetlands in Daxing'anling, northeast China [D], *Heilongjiang: Northeast Forestry University*. (in Chinese)
467. Lu, J., Z. Dong, W. Li, and G. Hu (2014), The effect of desertification on carbon and nitrogen status in the northeastern margin of the Qinghai-Tibetan Plateau, *Environmental Earth Sciences*, *71*(2), 807-815.
468. Lu, W. Z., G. X. Lin, C. M. Wang, W. Q. Wang, S. C. Yang, and G. H. Lin (2014), Comparative studies on carbon storage and litterfall dynamics between secondary and primary mangrove communities in Zhanjiang,Guangdong Provinces,China, *Marine Environmental Science*, *33*(6), 913-919. (in Chinese)
469. Lu, W. Z., S. C. Yang, L. Z. Chen, W. Q. Wang, X. N. Du, C. M. Wang, M. Yan, G. X. Lin, and G. H. Lin (2014), Changes in carbon pool and stand structure of a native subtropical mangrove forest after inter-planting with exotic species Sonneratia apetala, *PloS One*, *9*(3), e91238.
470. Lu, X., Y. Yan, J. Fan, Y. Cao, and X. Wang (2011), Dynamics of above- and below-ground biomass and C, N, P accumulation in the alpine steppe of Northern Tibet, *Journal of Mountain Science*, *8*(6), 838-844.
471. Lu, Y., and H. Xu (2014), Distribution characteristic of soil organic carbon fraction in different types of wetland in Hongze Lake of China, *The Scientific World Journal*, *2014*, e487961.
472. Luan, J., C. Xiang, S. Liu, Z. Luo, Y. Gong, and X. Zhu (2010), Assessments of the impacts of Chinese fir plantation and natural regenerated forest on soil organic matter quality at Longmen mountain, Sichuan, China, *Geoderma*, *156*(3–4), 228-236.
473. Lunstrum, A., and L. Chen (2014), Soil carbon stocks and accumulation in young mangrove forests, *Soil Biology & Biochemistry*, *75*(75), 223-232.
474. Luo, H. L., H. P. Wang, and H. Chen (2010), Change of farmland soil organic carbon density in hilly area of central Sichuan basin in the last 25 years- a case study of Yanting county, Sichuan province, *Journal of Mountain Science*, *28*(2), 212-217. (in Chinese)
475. Luo, K., R. G. Hu, W. J. Zhang, B. K. Zhou, M. G. Xu, J. Y. Zhang, and P. P. Xia (2013), Response of black soil organic carbon, nitrogen and its availability to long-term fertilization, *Environmental Science*, *34*(2), 676-684. (in Chinese)
476. Luo, T., S. Brown, Y. Pan, P. Shi, H. Ouyang, Z. Yu, and H. Zhu (2005), Root biomass along subtropical to alpine gradients: Global implication from Tibetan transect studies, *Forest Ecology & Management*, *206*(1-3), 349-363.
477. Luo, X. X., S. S. Zhang, and M. Dun (2010), Spatial distribution and seasonal dynamics characteristics of Carbon,Nitrogen and Phosphorus in the Liaohe estuary wetlands, *Periodical of Ocean University of China*, *40*(12), 97-104. (in Chinese)
478. Lv, H. B. (2013), The effect of restored Caragana korshinskii shrubwood on soil physicochemical properties in Loess area, *Ecology and Environmental Sciences*, *22*(1), 47-49. (in Chinese)
479. Lv, M. Z., L. X. Sheng, and L. Zhang (2013), A review on carbon fluxes for typical wetlands in different climates of China, *Wetland Science*, *11*(1), 114-120. (in Chinese)
480. Lv, M. Z., Z. J. Zhou, L. X. Sheng, and L. Zhang (2013), Differences in the biomass of ecological system of wetlands in the eastern margin of the Qinghai-Tibet Plateau, *Journal of Sichuan Forestry Science and Technology*, *34*(6), 22-26. (in Chinese)
481. Lv, X. T. (2006), A study on carbon storage of tropical seasonal rain forest in Xishuangbanna, Yunan [D], *Yunnan: Xishuangbanna Tropical Botanical Garden, CAS*. (in Chinese)
482. Lv, X. T., J. W. Tang, Y. C. He, W. G. Duan, J. P. Song, H. L. Xu, and S. Z. Zhu (2007), Biomass and its allocation in tropical seasonal rain forest in Xishuangbanna, southwest China, *Journal of Plant Ecology*, *31*(1), 11-22. (in Chinese)
483. Ma, H. B., Q. J. Shen, Y. Z. Xie, and Y. Shen (2013), Effects of the enclosing on the underground carbon storage of typical steppe in Ningxia, *Journal of Agricultural Sciences*, *34*(3), 1-4. (in Chinese)
484. Ma, H. L. (2013), Carbon density and its distribution characteristics of the main vegetation ecosystem in the northern of Shaanxi [D], *Shanxi: Northwest Agriculture and Forestry University*. (in Chinese)
485. Ma, H. P., Q. Q. Guo, H. M. Liu, and D. F. Qian (2013), Soil organic carbon pool at the western side of the sygera mountains ,southeast Tibet, China, *Acta Ecologica Sinica*, *33*(10), 3122-3128. (in Chinese)
486. Ma, J., J. Wang, H. Yao, Z. Feng, and X. Li (2013), Community characteristics and functional group diversity’s spatial variability of grassland wetland in China’s Hui River national nature reserve, *DISASTER ADVANCES*, *6*, 94-104.
487. Ma, J. M., S. C. Liang, Y. M. Liang, and F. J. Pan (2009), Aboveground biomass and its allocation of main shrub types in Karst hills of Guilin, China, *Journal of Guangxi Normal University: Natural Science Edition*, *27*(4), 95-98. (in Chinese)
488. Ma, K. (2011), Influence of poplar plantation on soil organic carbon in arid area [D], *Xinjiang: Xinjiang University*. (in Chinese)
489. Ma, L. (2012), Xiaoxing'anling birch natural forest ecosystem carbon storage research [D], *Heilongjiang: Northeast Forestry University*. (in Chinese)
490. Ma, L., L. Z. Yang, L. Z. Xia, M. X. Shen, S. X. Yin, and Y. D. Li (2011), Long-term effects of inorganic and organic amendments on organic carbon in a paddy soil of the Taihu Lake Region, China, *Pedosphere*, *21*(2), 186-196.
491. Ma, M. (2009), Researches on carbon distribution and carbon pool of main natural forest at Huoditang forestry region in the Qinliang mountains [D], *Shanxi: Northwest Agriculture and Forestry University*. (in Chinese)
492. Ma, T., G. L. Wu, Y. L. He, S. J. Wen, J. L. He, J. X. Liu, and G. Z. Du (2007), The effect of simulated mowing of the fertilizing level on community production and compensatory responses on the Qinghai-Tibetan, *Acta Ecologica Sinica*, *27*(6), 2288-2293. (in Chinese)
493. Ma, W. (2011), The analysis of organic carbon storage and influencing factors in different forest type in Liaoheyuan [D], *Hebei: Agricultural University of Hebei*. (in Chinese)
494. Ma, W. (2012), Measurement and estimation of ecosystem carbon density for Larix olgensis plantation based on FIM and FFE-FVS [D], *Beijing: Beijing Forestry University*. (in Chinese)
495. Ma, W., Y. J. Sun, X. Y. Guo, W. Z. Ju, and J. S. Mu (2010), Carbon storage of Larix olgensis plantation at different stand ages, *Acta Ecologica Sinica*, *30*(17), 4659-4667. (in Chinese)
496. Ma, W. W., H. Wang, R. Huang, J. Z. Li, and D. Y. Li (2014), Distribution of soil organic carbon storage and carbon density in Gahai wetland ecosystem, *Chinese Journal of Applied Ecology*, *25*(3), 738-744. (in Chinese)
497. Ma, W. W., H. Wang, Y. S. Wang, H. Wang, and H. R. Zhao (2012), Soil properties of meadow wetlands for different altitudes in Gahai of Gannan, *Acta Agrestia Sinica*, *20*(6), 1044-1050. (in Chinese)
498. Ma, Y. H. (2008), Effects of vegetation types on soil organic carbon in Loess hilly region [D], *Shanxi: Institute of soil and water conservation, CAS and MWR*. (in Chinese)
499. Ma, Z., H. Hartmann, H. Wang, Q. Li, Y. Wang, and S. Li (2014), Carbon dynamics and stability between native Masson pine and exotic slash pine plantations in subtropical China, *European Journal of Forest Research*, *133*(2), 307-321.
500. Ma, Z. Q., Q. J. Liu, W. J. Xu, X. R. Li, and Y. C. Liu (2007), Carbon storage of artificial forest in Qianyanzhou, Jiangxi province, *Scientia Silvae Sinicae*, *43*(11), 1-7. (in Chinese)
501. Ma, Z. R. (2013), Carbon storage and carbon sequestration rate of the main vegetation types in Liupan mountain [D], *Shanxi: Institute of soil and water conservation, CAS and MWR*. (in Chinese)
502. Man, X. L., B. Liu, and Y. Li (2010), Distribution characteristics of organic carbon, nitrogen and phosphorus in the soils of herbaceous peat swamps in the Xiaoxing'an mountains, *Journal of Beijing Forestry University*, *32*(6), 48-53. (in Chinese)
503. Mao, R., X. H. Zhang, and H. N. Meng (2014), Effect of *Suaeda salsa* on soil aggregate-associated organic carbon and nitrogen in tidal salt marshes in the Liaohe Delta, China, *Wetlands*, *34*(1), 189-195.
504. Mao, Z. G., G. X. Wang, J. E. Liu, and L. J. Ren (2009), Influence of salt marsh vegetation on spatial distribution of soil carbon and nitrogen in Yancheng coastal wetland, *Chinese Journal of Applied Ecology*, *20*(2), 293-297. (in Chinese)
505. Meng, L. (2010), Carbon storage and density of artificial Pinus tabulaeformis forest in Ziwuling area [D], *Shanxi: Northwest Agriculture and Forestry University*. (in Chinese)
506. Meng, Y. (2012), Reserves estimation and spatial distribution of soil organic carbon in small watershed scale [D], *Hubei: Huazhong Agricultural University*. (in Chinese)
507. Meng, Y. Y., Y. Bao, Y. Guo, X. Y. Wang, D. P. Yu, L. Zhou, and L. M. Dai (2014), Soil carbon and nitrogen content in windthrow area on Changbai mountain after 26 years' natural revovery, *Chinese Journal of Ecology*, *33*(7), 1757-1761. (in Chinese)
508. Mi, J., J. Li, D. Chen, Y. Xie, and Y. Bai (2014), Predominant control of moisture on soil organic carbon mineralization across a broad range of arid and semiarid ecosystems on the Mongolia plateau, *Landscape Ecology, 30*(9), 1683-1699.
509. Miao, J., C. Y. Zhou, S. J. Li, and J. H. Yan (2014), Accumulation of soil organic carbon and total nitrogen in *Pinus yunnanensis* forests at different age stages, *Chinese Journal of Ecology*, *25*(3), 625-631. (in Chinese)
510. Miao, X. L. (2013), Study on the potential for carbon sequestration of artificial PR107 Rubber forest ecosystem in rubber tree planting area of Qiongzhong of Hainan [D], *Hainan: Hainan University*. (in Chinese)
511. Ming, A. G., H. Y. Jia, Y. Tao, L. L. H., D. X. Cai, and Z. M. Shi (2012), Characteristics of carbon accumulation and allocation pattern in *Mytilaria laosensis* plantation, *Chinese Journal of Ecology*, *31*(11), 2730-2735. (in Chinese)
512. Ming, A. G., H. Y. Jia, Z. W. Tian, Y. Tao, L. H. Lu, D. X. Cai, Z. M. Shi, and W. X. Wang (2014), Characteristics of carbon storage and its allocation in *Erythrophleum fordii* plantations with different ages, *Chinese Journal of Applied Ecology*, *25*(4), 940-946. (in Chinese)
513. Miyasaka, T., T. Okuro, H. Zhao, X. Zhao, X. Zuo, and K. Takeuchi (2011), Impacts of the local land-use system in a semi-arid region of northeastern China on soil properties, crop growth, and weed communities, *Journal of Arid Environments*, *75*(11), 1155-1163.
514. Mo, D. X., K. B. Liao, Q. B. Wu, and J. Qin (2011), The carbon storage amount and spatial distribution characteristics of Paramichelia bailonii plantations, *Journal of Anhui Agricultural Sciences*, *39*(23), 14072-14075. (in Chinese)
515. Mo, D. X., Q. B. Wu, N. Lin, and Y. Zhuo (2012), Carbon and nitrogen storage and their allocation pattern in Cryptomeria fortunei plantations in southeastern Guangxi of south China, *Chinese Journal of Ecology*, *31*(4), 794-799. (in Chinese)
516. Mo, J. F., K. Tian, M. Lu, F. L. Chang, and N. Y. Li (2004), Study on the spatial variability of soil organic matter in Napahai degraded wetland, *Journal of Southwest Forestry College*, *24*(3), 25-28. (in Chinese)
517. Mu, C., H. Lu, B. Wang, X. Bao, and W. Cui (2013), Short-term effects of harvesting on carbon storage of boreal *Larix gmelinii-Carex schmidtii* forested wetlands in Daxing’anling, northeast China, *Forest Ecology and Management*, *293*, 140-148.
518. Mu, C. C., X. Bao, H. C. Lu, B. Wang, and W. Cui (2013), Short-Term Effects of Fire Disturbance on Carbon Storage of *Larix gmelinii-Carex schmidtii* Forested Wetlands Ecosystem in Daxing' an mountain, *Scientia Silvae Sinicae*, *49*(2), 8-14. (in Chinese)
519. Mu, C. C., B. Wang, H. C. Lu, X. Bao, and W. Cui (2013), Carbon storage of natural wetland ecosystem in Daxing' anling of China, *Acta Ecologica Sinica*, *33*(16), 4956-4965. (in Chinese)
520. Nan, X. X., D. H. You, X. H. Tian, J. Li, S. J. Wang, J. Cui, and T. Liu (2011), Effect of returning of cropland straw to field on soil organic carbon and grain yield in Guanzhong plain, *Acta Agriculturae Boreali-Sinica*, *26*(5), 222-229. (in Chinese)
521. Nan, Y. F., S. L. Guo, N. N. Li, Y. J. Zhang, A. X. Chen, L. Y. Rong, and J. Liu (2013), Soil organic carbon in croplands across different terrains in the Qinghai-Tibet Plateau, *Journal of Plant Nutrition and Fertilizer*, *19*(4), 946-954. (in Chinese)
522. Nian, Y. P., Z. M. Wang, T. Y. Xia, and Y. F. Nian (2014), Distribution and change pattern of soil organic carbon in vegetable field in Dianchi lake basin, *Southwest China Journal of Agricultural Sciences*, *27*(2), 724-728. (in Chinese)
523. Nie, Q., and G. T. Ren (2010), Research on forest biomass in Mudanjiang, *Journal of Heilongjiang Vocational Institute of Ecological Engineering*, *23*(2), 23-24. (in Chinese)
524. Ning, C. (2013), The research on carbon stock and nutrient of shrub forests in Karst regions [D], *Hunan: Central South University of Forestry & Technology*. (in Chinese)
525. Ning, F. (2009), Study on influence of disturbance manners to typical steppe vegetation and soil [D], *Beijing: Chinese Academy of Agricultural Sciences*. (in Chinese)
526. Niu, C. H. (2012), Study on biomass carbon storage in the mixed and pure stands of Pinus massoniana and Castanopsis hystrix [D], *Guangxi: Guangxi University*. (in Chinese)
527. Niu, D., S. J. Hall, H. Fu, J. Kang, Y. Qin, and J. J. Elser (2011), Grazing exclusion alters ecosystem carbon pools in Alxa desert steppe, *New Zealand Journal of Agricultural Research*, *54*(3), 127-142.
528. Niu, D., S. Wang, Z. Ouyang (2009), Comparisons of carbon storages in *Cunninghamia lanceolata* and *Michelia macclurei* plantations during a 22-year period in southern China, *Journal of Environmental Sciences*, *21*(6), 801-805.
529. Niu, R. X., X. Y. Zhao, J. L. Liu, and Y. Qin (2013), Effects of land use/cover change in the desert oasis system on topsoil carbon and nitrogen (middle of Heihe River basin, China), *Polish Journal of Ecology*, *61*(1), 45-54.
530. Niu, Y., X. D. Liu, W. J. Zhao, and X. L. Zhang (2014), Characteristics and interrelation of shallow soil organic and total nitrogen of Picea crassifolia forest in the Qilian mountain, Gansu, China, *Journal of Desert Research*, *34*(2), 371-377. (in Chinese)
531. Ouyang, L. M., D. P. Zeng, Q. W. Min, W. Q. Wang, and C. Tong (2014), Ecological stoichiometry characteristics of soil carbon, nitrogen and phosphorus in the tea garden of Drum Mountain, *Journal of Soil and Water Conservation*, *28*(2), 297-301. (in Chinese)
532. Pan, B. B. (2013), Research on carbon storage of major aquatic plant communities [D], *Jiangsu: Nanjing Forestry University*. (in Chinese)
533. Pan, B. B., J. C. Zhang, K. Y. Feng, J. D. Chen, and X. P. Guo (2014), Carbon storage of typical aquatic plant communities in Hungtse lake, *Wetland Science*, *12*(4), 471-476. (in Chinese)
534. Pan, P., D. Lv, X. Z. Ouyang, and X. T. Wang (2014), A study on biomass and carbon storage of natural Pinus massoniana forest at different stand growing stages in central Jiangxi province, *Acta Agriculturae Universitatis Jiangxiensis*, *36*(1), 131-136. (in Chinese)
535. Pan, Y. J., B. Wang, B. F. Chen, and Q. Z. Peng (2013), Study on carbon sink of Chinese fir plantation ecosystem in Dagangshan mountain, Jiangxi province, *Journal of Central South Forestry University*, *33*(10), 120-125. (in Chinese)
536. Pang, J. P. (2009), Carbon storage and its allocation of rubber plantation in Xishuangbanna, southwest China. [D], *Yunnan: Xishuangbanna Tropical Botanical Garden, CAS*. (in Chinese)
537. Pang, S. L., Z. Y. Ou, H. N. Mo, Y. R. Hou, and G. D. Lu (2014), Biomass and productivity of 3 typical shrub communities in karst areas of Western Guangxi, *Journal of Central South University of Forestry & Technology*, *34*(9), 86-90. (in Chinese)
538. Pei, S., H. Fu, C. Wan, Y. Chen, and R. E. Sosebee (2006), Observations on changes in soil properties in grazed and nongrazed areas of *Alxa* desert steppe, Inner Mongolia, *Arid Land Research and Management*, *20*(2), 161-175.
539. Pei, Z. Q., C. W. Xiao, D. Dong, and S. R. Zhang (2012), Comparison of the fine root dynamics of Populus euphratica forests in different habitats in the lower reaches of the Tarim River in Xinjiang, China, during the growing season, *Journal of Forest Research*, *17*(4), 343-351.
540. Peng, H. Y., S. Y. Li, and S. Y. Tong (2013), Effects of shrub encroachment on biomass and biodiversity in the typical steppe of Inner Mongolia, *Acta Ecologica Sinica*, *33*(22), 7221-7229. (in Chinese)
541. Peng, P. Q., W. J. Zhang, C. L. Tong, S. J. Qiu, and W. C. Zhang (2005), Soil C, N and P contents and their relationships with soil physical properties in wetlands of Dongting Lake floodplain, *Chinese Journal of Applied Ecology, 16*(10), 1872-1878. (in Chinese)
542. Peng, S., A. Chen, H. Fang, J. Wu, and G. Liu (2013), Effects of vegetation restoration types on soil quality in Yuanmou dry-hot valley, China, *Soil Science & Plant Nutrition*, *59*(3), 347-360.
543. Peng, X. W. (2012), Study on carbon storage of immature grass-larch forest in Daxing'anling district, *Forestry Science and Technology Information*(4), 22-23. (in Chinese)
544. Peng, X. X. (2011), Differences in physical and chemical properties and organic carbon stability of black soil between cropping systems in northeast China [D], *Jiangsu: Nanjing Agricultural University*. (in Chinese)
545. Qi, B. (2005), Study on the soil carbon storage of alpine grassland under different degrees of degradation in Qinghai lake region [D], *Gansu: Gansu Agricultural University*. (in Chinese)
546. Qi, G. (2011), Carbon pool and sequestration potential of larch plantations in Northeast China [D], *Liaoning: Institute of Applied Ecology, CAS*. (in Chinese)
547. Qi, G., Q. L. Wang, X. C. Wang, D. P. Yu, L. Zhou, W. M. ZHou, S. L. Peng, and L. M. Dai (2013), Soil organic carbon storage in different aged Larix gmelinii plantations in Great Xing'an mountains of northeast China, *Chinese Journal of Applied Ecology*, *24*(1), 10-16. (in Chinese)
548. Qi, J. W., and J. W. Tang (2008), Biomass and its allocation pattern of monsoon rain forest over limestone in Xishuangbanna of Southwest China, *Chinese Journal of Ecology*, *27*(2), 167-177. (in Chinese)
549. Qi, L., D. P. Yu, W. M. Zhou, L. Zhou, F. A. Zhao, C. H. Wang, and L. M. Dai (2013), Impact of logging on carbon density of broadleaved-Korean pine mixed forests on Changbai mountains, *Acta Ecologica Sinica*, *33*(10), 3065-3073. (in Chinese)
550. Qi, L. H., M. Y. Du, S. H. Fan, X. H. Yue, W. S. Ai, Y. Meng, and M. Yang (2012), Dynamics of soil organic carbon pool in *Phyllostachy edulis* forest and P. edulis-Cunning-hamia lanceolata mixed forest in hilly regions of central Hunan, Southern China, *Chinese Journal of Ecology*, *31*(12), 3038-3043. (in Chinese)
551. Qi, Y., Y. Dong, J. Zhao, Q. Peng, S. Xiao, and Y. He (2010), Spatial heterogeneity of soil nutrients and respiration in the desertified grasslands of Inner Mongolia, China, *Pedosphere*, *20*(5), 655-665.
552. Qi, Y., Y. M. Huang, Y. Wang, J. Zhao, and J. H. Zhang (2011), Biomass and its allocation of four grassland species under different nitrogen levels, *Acta Ecologica Sinica*, *31*(18), 5121-5129. (in Chinese)
553. Qi, Y. C., Q. Peng, Y. S. Dong, S. S. Xiao, L. J. Sun, X. C. Liu, Y. T. He, J. Q. Jia, and C. C. Cao (2014), Responses of soil total organic carbon and dissolved organic carbon to simulated nitrogen deposition in temperate typical steppe in Inner Mongolia, China, *Environmental Science*, *35*(8), 3073-3082. (in Chinese)
554. Qin, J. H., Q. Wang, and H. Sun (2013), Changes of organic carbon and its labile fractions in topsoil with altitude in subalpine -alpine area of southwestern China, *Acta Ecologica Sinica*, *33*(18), 5858-5864. (in Chinese)
555. Qin, J. H., Y. Zhang, Y. C. Zhao, Z. J. Wang, C. X. Li, and H. N. Gao (2014), Soil physicochemical properties and variations of nutrients and enzyme activity in the degrading grasslands in the upper reaches of the Heihe river, Qilian Mountains, *Journal of Glaciology and Geocryology*, *36*(2), 335-346. (in Chinese)
556. Qin, Y. (2010), Effect of different land utilization on soil nutrients and grassland vegetation in alpine meadow [D], *Gansu: Lanzhou Univeristy*. (in Chinese)
557. Qin, Y., S. Yi, S. Ren, N. Li, and J. Chen (2014), Responses of typical grasslands in a semi-arid basin on the Qinghai-Tibetan Plateau to climate change and disturbances, *Environmental Earth Sciences*, *71*(3), 1421-1431.
558. Qiu, L., X. Wei, X. Zhang, and J. Cheng (2013), Ecosystem carbon and nitrogen accumulation after grazing exclusion in semiarid grassland, *PloS one*, *8*(1), 268-277.
559. Qiu, S., X. Ju, J. Ingwersen, Z. Qin, L. Li, T. Streck, P. Christie, and F. Zhang (2010), Changes in soil carbon and nitrogen pools after shifting from conventional cereal to greenhouse vegetable production, *Soil and Tillage Research*, *107*(2), 80-87.
560. Qiu, Y. J., M. X. Xu, C. D. Shi, Z. X. Zhang, and S. Zhang (2014), Dynamic accumulation of soil organic carbon of terrace changed from slope cropland in the hilly Loess Plateau of eastern Gansu province, *Journal of Plant Nutrition and Fertilizer*, *20*(1), 87-98. (in Chinese)
561. Qu, F., J. Yu, S. Du, Y. Li, X. Lv, K. Ning, H. Wu, and L. Meng (2014), Influences of anthropogenic cultivation on C, N and P stoichiometry of reed-dominated coastal wetlands in the Yellow River Delta, *Geoderma*, *235*, 227-232.
562. Qu, H., X. Y. Zhao, S. K. Wang, W. D. Huang, and W. Mao (2014), Effects of different vegetation communities on soil carbon and nitrogen contents in Urad desert steppe, *Pratacultural Science*, *31*(3), 355-360. (in Chinese)
563. Ren, A. C. (2008), Grassland biomass on north-western plateau of Sichuan and vegetation indexes relation using landsat TM image [D], *Sichuan: Sichuan Agricultural University*. (in Chinese)
564. Ren, H., C. Z. Zhao, F. Y. Gao, F. X. Shi, and Q. Zhang (2012), Spatial pattern of sexual plants and vegetative plants of *Stipa krylovii* population in alpine degraded grassland, *Acta Ecologica Sinica*, *32*(21), 6909-6916. (in Chinese)
565. Ren, J. H. (2011), The study on carbon storage and carbon density of natural Pinus Tabuliformis forest of mountain Helan in Ningxia province [D], *Shanxi: Northwest Agriculture and Forestry University*. (in Chinese)
566. Ren, J. J., J. Li, X. C. Wang, and X. Y. Fang (2011), Soil water and nutrient characteristics of alfalfa grasslands at semi-arid and semi-arid prone to drought areas in southern Ningxia, *Acta Ecologica Sinica*, *31*(13), 3638-3649. (in Chinese)
567. Ren, Y. H., J. Cai, J. Yuan, and S. X. Zhang (2012), Carbon storage and density forest at Huoditang forest of tree layer of three types of region in the Qinling Mountains, *Journal of Henan Agricultural Sciences*, *41*(9), 73-77. (in Chinese)
568. Rong, J. R. (2012), Effect of long-term fertilization on characteristics of soil carbon cools change and aggregates distribution, oasis farmland [D], *Jiangsu: Nanjing Agricultural University*. (in Chinese)
569. Rong, J. R., C. H. Li, Y. G. Wang, L. S. Tang, and X. M. Chen (2012), Effect of long-term fertilization on soil organic carbon and soil inorganic carbon in oasis cropland, *Arid Zone Research*, *29*(4), 592-597. (in Chinese)
570. Rong, Y., F. Yuan, and L. Ma (2014), Effectiveness of exclosures for restoring soils and vegetation degraded by overgrazing in the Junggar Basin, China, *Grassland Science*, *60*(2), 118–124.
571. Sa, W., L. An, and S. Wei (2012), Changes in plant community diversity and aboveground biomass along with altitude within an alpine meadow on the Three-River source region, *Chinese Science Bulletin*, *57*(27), 3573-3577.
572. Sa, R. L. (2013), Effects of different utilization and grazing intensity on carbon storage of vegetation-soil system in typical steppe [D], *Beijing: Chinese Academy of Agricultural Sciences*. (in Chinese)
573. Sa, R. L., X. Y. Hou, J. X. Li, Y. Ding, X. H. Wu, and X. J. Yun (2013), Organic carbon storage in vegetation-soil systems of typical grazing degraded steppes, *Acta Prataculturae Sinica*, *22*(5), 18-26. (in Chinese)
574. Sha, L. Q. (2008), Carbon storage and soil CO_2_ efflux of tropical seasonal rain forest, rubber tree plantation and paddy soil in Xishuangbanna. southwest China [D], *Yunnan: Xishuangbanna Tropical Botanical Garden, CAS*. (in Chinese)
575. Shang, L. N. (2004), Fire effects on properties of wetland soil in Sanjiang Plain [D], *Jilin: Northeast Normal University*. (in Chinese)
576. Shang, S. Y., P. K. Jiang, Z. L. Song, Y. F. Li, and L. Lin (2013), Composition and stability of organic carbon in the top soil under different forest types in subtropical China, *Acta Ecologica Sinica*, *33*(2), 416-424. (in Chinese)
577. Shang, S. Y., Y. F. Li, P. K. Jiang, G. M. Zhou, J. D. Liu, J. S. Wu, and L. Lin (2012), Effects of the conversion from native shrub forest to Chinese chestnut plantation on soil carbon and nitrogen pools, *Chinese Journal of Applied Ecology*, *23*(3), 659-665. (in Chinese)
578. Shang, Z., J. Cao, R. Guo, and R. Long (2012), Effects of cultivation and abandonment on soil carbon content of subalpine meadows, northwest China, *Journal of Soils and Sediments*, *12*(6), 826-834.
579. Shang, Z., J. Cao, R. Guo, R. Long, and B. Deng (2014), The response of soil organic carbon and nitrogen 10years after returning cultivated alpine steppe to grassland by abandonment or reseeding, *Catena*, *119*(1), 28-35.
580. Shang, Z., Q. Feng, G. Wu, G. Ren, and R. Long (2013), Grasslandification has significant impacts on soil carbon, nitrogen and phosphorus of alpine wetlands on the Tibetan Plateau, *Ecological engineering*, *58*, 170-179.
581. Shao, M. X., S. Z. Wen, G. X. He, X. Z. zhao, and Q. Ouyang (2014), The biomass structure characteristics of P. bournei (Hemsl.) Yang plantation in different ages, *Journal of Central South Forestry University*, *34*(6), 44-48. (in Chinese)
582. Shao, X., M. Wu, B. Gu, Y. Chen, and X. Liang (2013), Nutrient retention in plant biomass and sediments from the salt marsh in Hangzhou Bay estuary, China, *Environmental Science and Pollution Research*, *20*(9), 6382-6391.
583. Shao, X. X., W. Y. Yang, M. Wu, and K. Y. Jiang (2011), Soil organic carbon content and its distribution pattern in Hangzhou Bay coastal wetlands, *Chinese Journal of Applied Ecology*, *22*(3), 658-664. (in Chinese)
584. Shao, Y. H. (2005), Soil organic carbon dynamics and validation of InTEC model in China's subtropical and temperate zones [D], *Jiangsu: Nanjing Agricultural University*. (in Chinese)
585. Shao, Y. H., J. J. Pan, X. W. Xu, and L. X. Yang (2006), Determination of forest soil organic carbon pool sizes and turnover rates in Changbaishan, *Journal of Soil and Water Conservation*, *20*(6), 99-102. (in Chinese)
586. She, D., and M. Shao (2009), Spatial variability of soil organic C and total N in a small catchment of the Loess Plateau, China, *Acta Agriculturae Scandinavica Section B-Soil and Plant Science*, *59*(6), 514-524.
587. Shen, B. (2013), Carbon density of Pinus tabulaeformis and Quercus aliena var. acuteserrata forest ecosystems on the southern slope of the middle Qinling mountains [D], *Shanxi: Northwest Agriculture and Forestry University*. (in Chinese)
588. Shen, C. (2012), The research on carbon balance of farmland shelterbelts under different afforestation way in the Henna eastern plain [D], *Henan: Henan Agricultural University*. (in Chinese)
589. Shen, H., W. Zhang, Y. Xue, X. Liu, J. Cao, X. Zeng, Z. Xin, X. Chen, and W. Zhang (2014), Carbon storage capacity of different plantation types under sandstorm source control program in Hebei Province, China, *Chinese Geographical Science*, *24*(4), 454-460.
590. Shen, J. P., and W. H. Zhang (2014), Characteristics of carbon storage and sequestration of Robinia pseudoacacia forest land converted by farmland in the Hilly Loess Plateau region, *Acta Ecologica Sinica*, *34*(10), 2746-2754. (in Chinese)
591. Shen, J. P., W. H. Zhang, Y. H. Li, J. J. You, B. Y. Yu, X. Z. Yang, and J. F. He (2013), Characteristics of carbon storage and sequestration of Pinus tabulaeformis forest land converted by farmland in Loess hilly area, *Acta Botanica Boreali-Occidentalia Sinica*, *33*(11), 2309-2316. (in Chinese)
592. Shen, M., Y. Tang, J. Klein, P. Zhang, S. Gu, A. Shimono, and J. Chen (2008), Estimation of aboveground biomass using in situ hyperspectral measurements in five major grassland ecosystems on the Tibetan Plateau, *Journal of Plant Ecology*, *1*(4), 247-257.
593. Sheng, H. (2007), Carbon pools and belowground carbon balance of mid-subtropical evergreen broad-leaved forest [D], *Fujian: Fujian Normal University*. (in Chinese)
594. Shi, C. (2012), The factor on carbon stocks of Platyclatdus orientalis plantation in Xuzhou [D], *Jiangsu: Nanjing Forestry University*. (in Chinese)
595. Shi, C. D., M. X. Xu, Y. J. Qiu, Z. X. Zhang, and X. W. Zhang (2014), Changes and influencing factors of the soil organic carbon in farmland in the last 30 years on hilly Loess Plateau: a case study in Zhuanglang county, Gansu province, *Environmental Science*, *35*(3), 1098-1104. (in Chinese)
596. Shi, F., H. Chen, Y. Wu, and N. Wu (2010), Effects of livestock exclusion on vegetation and soil properties under two topographic habitats in an alpine meadow on the eastern Qinghai-Tibetan Plateau, *Polish Journal of Ecology*, *58*(1), 125-133.
597. Shi, F. C., J. J. Li, and S. Q. Wang (2008), Soil organic carbon, nitrogen and microbial properties in contrasting forest ecosystems of north-east China under different regeneration scenarios, *Acta Agriculturae Scandinavica, Section B - Soil & Plant Science*, *58*(1), 1-10.
598. Shi, F. C., R. L. Li, S. Q. Wang, and K. Sasa (2007), Profile distribution and accumulation characteristics of organic carbon and total nitrogen in typical marshes in Sanjiang Plain, *Chinese Journal of Applied Ecology*, *18*(7), 1425-1431. (in Chinese)
599. Shi, F. S., N. Wu, and P. Luo (2008), Effect of temperature enhancement on community structure and biomass of subalpinemeadow in Northwestern Sichuan, *Acta Ecologica Sinica*, *28*(11), 5286-5293. (in Chinese)
600. Shi, H., Z. Wen, and D. Paull (2013), Estimation of carbon carrying capacity in the Yanhe River catchment of China's Loess Plateau, *Acta Agriculturae Scandinavica, Section B-Soil & Plant Science*, *63*(6), 543-553.
601. Shi, P. J. (2013), Characteristics of carbon and nitrogen of the topsoil under different vegetations in Guansi river basin [D], *Sichuan: Sichuan Agricultural University*. (in Chinese)
602. Shi, S. D. (2012), Biomass and productivity of typical tree species at different age groups in eastern Inner Mongolia [D], *Inner: Inner Mongolia Agricultural University*. (in Chinese)
603. Shi, X. H. (2012), CO2 emissions and soil organic carbon contents of cropland soil under different tillage practices- case studies on a black soil in northeast China and a Argiaquoll in Ontario, Canada [D], *Jilin: Northeast Institute of Geography and Agroecology, CAS*. (in Chinese)
604. Shi, Z. J., D. P. Xu, J. X. Gao, A. Y. Song, C. T. Yu, N. N. Zhang, and Z. S. Hu (2011), Carbon storage and its distribution of *Eucalyptus urophylla* × *E.tereticornis* plantations in Hainan island, southern China, *Scientia Silvae Sinicae*, *47*(10), 21-28. (in Chinese)
605. Shi, Z. L., Y. Q. Wang, Y. L. Ran, L. Zhang, J. B. Yu, and C. Q. Jiao (2013), Variation of soil organic carbon pool in apple orchards in Weibei, *Acta Pedologica Sinica*, *50*(1), 203-207. (in Chinese)
606. Si, J. (2012), Carbon sequestration and sink enhancement technologyes of several kinds of Poplar plantations in the north of China [D], *Beijing: Beijing Forestry University*. (in Chinese)
607. Song, Q. H., and Y. P. Zhang (2010), Biomass, carbon sequestration and its potential of rubber plantations in Xishuangbanna, southwest China, *Chinese Journal of Ecology*, *29*(10), 1887-1891. (in Chinese)
608. Song, Y., C. Song, Y. Li, C. Hou, G. Yang, and X. Zhu (2013), Short-term effects of nitrogen addition and vegetation removal on soil chemical and biological properties in a freshwater marsh in Sanjiang Plain, Northeast China, *Catena*, *104*, 265-271.
609. Song, Y., C. Song, B. Tao, J. Wang, X. Zhu, and X. Wang (2014), Short-term responses of soil enzyme activities and carbon mineralization to added nitrogen and litter in a freshwater marsh of Northeast China, *European Journal of Soil Biology*, *61*, 72-79.
610. Song, Y., C. Song, G. Yang, Y. Miao, J. Wang, and Y. Guo (2012), Changes in labile organic carbon fractions and soil enzyme activities after marshland reclamation and restoration in the Sanjiang Plain in Northeast China, *Environmental management*, *50*(3), 418-426.
611. Su, A. L. (2011), Analysis on the spatial and temporal variation characteristics of carbon sequestration in Phyllostachys edulis plantation ecosystem of Fujian [D], *Fujian: Fujian Agriculture and Forestry University*. (in Chinese)
612. Su, D. X. (2012), Divergent changes of soil organic and inorganic carbon and their influences on soil physicochemical properties [D], *Heilongjiang: Northeast Forestry University*. (in Chinese)
613. Su, S. C., D. J. He, Y. L. Xie, S. Z. Li, Y. P. Dong, J. Yang, Q. B. Wang, Z. R. Zhang, and X. J. Liao (2012), Comparative study on the carbon storage of different forest management modes in northern Fujian, *Chinese Agricultural Science Bulletin*, *28*(22), 45-52. (in Chinese)
614. Su, Y., Y. Li, J. Cui, and W. Zhao (2005), Influences of continuous grazing and livestock exclusion on soil properties in a degraded sandy grassland, Inner Mongolia, northern China, *Catena*, *59*(3), 267-278.
615. Su, Y., H. Zhao, T. Zhang, and X. Zhao (2004), Soil properties following cultivation and non-grazing of a semi-arid sandy grassland in northern China, *Soil & Tillage Research*, *75*(1), 27-36.
616. Su, Z. Y., Y. M. Xiong, J. Y. Zhu, Y. C. Ye, M. Ye (2006), Soil organic carbon content and distribution in a small landscape of Dongguan, South China, *Pedosphere*, *16*(1), 10-17.
617. Sun, D., K. Wesche, D. Chen, S. Zhang, G. Wu, G. Du, and N. Comerford (2011), Grazing depresses soil carbon storage through changing plant biomass and composition in a Tibetan alpine meadow, *Plant Soil & Environment*, *57*(6), 271-278.
618. Sun, J. C. (2011), Study on biomass and carbon stock in Pinus tabulaeformis plantation of Taiyue mountain [D], *Beijing: Beijing Forestry University*. (in Chinese)
619. Sun, T. (2011), Soil carbon flux and storage were measured across a chronosequence of secondary forests dominated by Betula platyphylla in Xiaoxing'an mountain, China [D], *Heilongjiang: Northeast Forestry University*. (in Chinese)
620. Sun, W. Y. (2010), The spatial distribution of soil organic carbon and it's influencing factors in small watershed of hilly region of Loess Plateau [D], *Shanxi: Institute of soil and water conservation, CAS and MWR*. (in Chinese)
621. Tan, G. X., Y. Q. Liu, L. L. Li, W. Liu, Y. T. Zan, B. N. Huo, and M. J. He (2014), Effects of stand structure regulation on soil labile organic carbon in Pinus elliottii plantation, *Chinese Journal of Applied Ecology*, *25*(5), 1307-1312. (in Chinese)
622. Tan, Q. J., T. Q. Song, W. X. Peng, F. P. Zeng, H. Du, G. R. Yang, and F. J. Fan (2014), Stability and organic carbon characteristics of soil aggregates under different ecosystems in karst canyon region, *Chinese Journal of Applied Ecology*, *25*(3), 671-678. (in Chinese)
623. Tan, W. N. (2009), Carbon sequestration in soils of several plantations in south China [D], *Guangdong: South China Botanical Garden, CAS*. (in Chinese)
624. Tang, F. K., J. X. Zhou, M. Cui, Y. G. Liu, and R. G. Lei (2014), Effects of different returning farmland to forestlands on accumulation of soil organic carbon and nitrogen in typical karst area of southwestern China, *Journal of Beijing Forestry University*, *36*(2), 44-50. (in Chinese)
625. Tang, G. Y. (2008), Spatial patterns and driving factors of soil organic carbon at typical landscapes in subtropical region in China [D], *Hunan: Institute of Subtropical Agriculture, CAS*. (in Chinese)
626. Tang, J., N. Zhang, Z. Y. Li, Z. L. Mao, N. Li, X. M. Xu, and W. Z. Han (2011), Vertical distribution of soil organic carbon and carbon density under different land use types in western Jilin province, *Journal of Jilin University (Earth Science Edition)*, *41*(4), 1151-1156. (in Chinese)
627. Tang, J. W., J. X. Yin, J. F. Qi, M. R. Jepsen, and X. T. Lü (2012), Ecosystem carbon storage of tropical forests over limestone in Xishuangbanna, SW China, *Journal of Tropical Forest Science*, *24*(24), 399-407.
628. Tang, L., X. Dang, G. Liu, C. Shao, and S. Xue (2014), Response of artificial grassland carbon stock to management in mountain region of Southern Ningxia, China, *Chinese Geographical Science*, *24*(24), 436-443.
629. Tang, X., S. Liu, and G. Zhou (2010), Erosion and vegetation restoration impacts on ecosystem carbon dynamics in South China, *Soil Science Society of America Journal*, *74*(1), 272-281.
630. Tang, X. L. (2006), Carbon stocks and allocation patterns in successional subtropical forests in southern China using experimental and modeling approaches [D], *Guangdong: South China Botanical Garden, CAS*. (in Chinese)
631. Tang, X. L., S. H. Fan, L. H. Qi, G. L. Liu, F. Y. Guan, M. Y. Du, and C. X. Shen (2012), Effect of different managements on carbon storage and carbon allocation in Moso bamboo forest (*Phyllostachys pubescen*), *Acta Agriculturae Universitis Jiangxiensis*, *34*(4), 736-742. (in Chinese)
632. Tao, B., and C. Song (2013), Temperature sensitivity of carbon dioxide production in aggregates and their responses to nitrogen addition in a freshwater marsh, Sanjiang Plain, *Soil science and plant nutrition*, *59*(6), 953-960.
633. Tao, Y., and Y. M. Zhang (2011), Seasonal changes in species composition, richness and the aboveground biomass of three community types Gurbantunggut desert, northwestern China, *Acta Prataculturae Sinica*, *20*(6), 1-11. (in Chinese)
634. Tao, Y. H. (2012), The changes of forest carbon storage under different land use in Luocheng, Guangxi province [D], *Beijing: Minzu University of China*. (in Chinese)
635. Tao, Y. H., J. C. Feng, S. G. Cao, Q. Guo, and D. Y. Xiang (2012), Study on carbon storage of Pinus massoniana,Cunninghamia lanceolata plantations at Shatang,Guangxi Province, *Journal of Northwest A & F University(Natural Science Edition)*, *40*(5), 38-44. (in Chinese)
636. Tao, Y. H., J. C. Feng, L. Y. Ma, W. G. Long, and S. G. Cao (2011), Carbon storage and distribution of massion pine, Chinese fir and eucalyptus plantations at Luocheng, Guangxi province, *Ecology and Environmnet*, *20*(11), 1608-1613. (in Chinese)
637. Tao, Y. H., W. G. Long, L. Y. Ma, S. G. Cao, D. Y. Xiang, and Q. Guo (2011), Carbon storage and distribution of massion pine, Chinese fir and eucalyptus plantations at Liuzhou,Guangxi province, *Guangdong Agricultural Sciences*, *22*, 42-45. (in Chinese)
638. Tao, Y. H., J. Z. Feng, L. Y. Ma, S. G. Cao, W. G. Long, and Q. Guo (2012), Study on carbon storage of eucalyptus plantations in short period in Liuzhou,Guangxi province, *Chinese Agricultural Science Bulletin*, *28*(7), 80-84. (in Chinese)
639. Tao, Z., C. D. Shen, Q. Z. Gao, Y. M. Sun, W. X. Yi, and Y. N. Li (2006), Soil organic carbon storage and vertical distribution of alpine meadow on the Tibetan plateau, *Acta Geographica Sinica*, *61*(7), 720-728. (in Chinese)
640. Tian, D. L., X. K. Wang, X. Fang, W. D. Yan, X. B. Ning, and G. J. Wang (2011), Carbon storage and spatial distribution in different vegetation restoration patterns in karsts area,Guizhou province, *Scientia Silvae Sinicae*, *47*(9), 7-14. (in Chinese)
641. Tian, J., D. P. Yu, L. Zhou, W. M. Zhou, J. Jia, J. Q. Liu, and L. M. Dai (2012), Carbon density of forest ecosystems in mountainous region of east Liaoning province, northeast China, *Chinese Journal of Ecology*, *31*(11), 2723-2729. (in Chinese)
642. Tian, K., F. L. Chang, M. Lu, J. F. Mo, and Y. X. Yang (2004), Impacts of human disturbances on organic carbon and nitrogen in Napahai wetlands ,northwest Yunnan, *Acta Pedologica Sinica*, *41*(5), 681-686. (in Chinese)
643. Tian, W. W., W. Wang, A. L. Chen, Y. Y. Li, Y. Y. Li, and X. L. Xie (2014), Early responses of vegetation and soil organic carbon to waterlogging and winter wildfire on abandoned red paddy soils, *Chinese Journal of Plant Ecology*, *38*(6), 626-634. (in Chinese)
644. Tian, X. (2011), Study on the carbon storage and NPP in plantations Phoebe bournei based on FORECAST model [D], *Jiangxi: Jiangxi Agricultural University*. (in Chinese)
645. Tian, Y. W., Z. L. Huang, and W. F. Xiao (2012), Effects of plant species and litter on soil organic carbon sequestration in converted croplands in a typical watershed in Three Gorges Reservoir area of China, *Chinese Journal of Ecology*, *31*(11), 2742-2747. (in Chinese)
646. Tong, C., L. Zhang, W. Wang, V. Gauci, R. Marrs, B. Liu, R. Jia, and C. Zeng (2011), Contrasting nutrient stocks and litter decomposition in stands of native and invasive species in a sub-tropical estuarine marsh, *Environmental research*, *111*(7), 909-916.
647. Tong, X. G., X. H. Han, F. Q. Wu, Y. Y. Zhang, X. L. Yu, and B. Jiang (2012), Variance analysis of soil carbon sequestration under three typical forest lands converted from farmland in a Loess Hilly Area, *Acta Ecologica Sinica*, *32*(20), 6396-6403. (in Chinese)
648. Wan, X. H., Z. Q. Huang, Z. M. He, Z. H. Hu, J. Y. Yang, Z. P. Yu, and M. H. Wang (2013), Effects of broadleaf plantation and Chinese fir (*Cunninghamia lanceolata*) plantation on soil carbon and nitrogen pools, *Chinese Journal of Applied Ecology*, *24*(2), 345-350. (in Chinese)
649. Wan, Z. M., and C. C. Song (2008), Soil enzyme activity and its relationship with the soil nutrient environment of different types wetland in Sanjiang Plain, *Journal of Soil and Water Conservation*, *22*(5), 158-161. (in Chinese)
650. Wang, B. (2013), Carbon storage of natural wetland ecosystem in southern Daxing' an mountains of China [D], *Heilongjiang: Northeast Forestry University*. (in Chinese)
651. Wang, B., Q. P. Yang, Q. R. Guo, G. D. Zhao, and K. Fang (2011), Carbon storage and allocation of Phyllostachys edulis forest and evergreen broad-leaved forest in Dagangshan mountain,Jiangxi, *Guihaia*, *31*(3), 342-348. (in Chinese)
652. Wang, B. X., C. S. Zeng, D. Chen, W. Q. Wang, and L. H. Zhang (2010), Effects of spartina alterniflora invasion on soil organic carbon in the Phragmites australis estuary wetlands of Minjiang River, *Science of Soil and Water Conservation*, *8*(5), 114-118. (in Chinese)
653. Wang, C. (2011), Study on carbon density of Populus tomentosa plantations at different ages on Loess Plateau [D], *Shanxi: Northwest Agriculture and Forestry University*. (in Chinese)
654. Wang, C., G. Cao, Q. Wang, Z. Jing, L. Ding, and R. Long (2008), Changes in plant biomass and species composition of alpine Kobresia meadows along altitudinal gradient on the Qinghai-Tibetan Plateau, *Science in China Series C: Life Sciences*, *51*(1), 86-94.
655. Wang, C., R. Long, Q. Wang, Z. Jing, and J. Shi (2009), Changes in plant diversity, biomass and soil C, in alpine meadows at different degradation stages in the headwater region of three rivers, China, *Land Degradation & Development*, *20*(2), 187-198.
656. Wang, C. M., H. Ouyang, B. Shao, Y. Q. Tian, J. G. Zhao, and H. Y. Xu (2006), Soil Carbon Changes Following Afforestation with Olga Bay Larch ( *Larix olgensis Henry*) in Northeastern China, *Journal of Integrative Plant Biology*, *48*(5), 503-512.
657. Wang, C. M., B. Shao, and R. N. Wang (2010), Carbon sequestration potential of ecosystem of two main tree species in northeast China, *Acta Ecologica Sinica*, *30*(7), 1764-1772. (in Chinese)
658. Wang, C. T. (2006), Relationship between productivity and species diversity of plant community in alpine meadow [D], *Qinghai: Northwest institute of plateau biology, CAS*. (in Chinese)
659. Wang, C. T., R. J. Long, G. X. Wang, W. Liu, Q. L. Wang, L. Zhang, and P. F. Wu (2010), Relationship between plant communities, characters, soil physical and chemical properties and soil microbiology in apline meadow, *Acta Prataculturae Sinica*, *19*(6), 25-34. (in Chinese)
660. Wang, C. Y. (2011), Soil carbon storage and soil respiration of rubber plantations at different ages [D], *Hainan: Hainan University*. (in Chinese)
661. Wang, D., Z. C. Geng, D. She, W. X. He, and L. Hou (2014), Vertical distribution of soil active carbon and soil organic carbon storage under different forest types in the Qinling mountains, *Chinese Journal of Applied Ecology*, *25*(6), 1569-1577. (in Chinese)
662. Wang, F. (2013), Research on the carbon density and carbon balance of Larix gmelinii forest [D], *Inner: Inner Mongolia Agricultural University*. (in Chinese)
663. Wang, F., X. Xu, B. Zou, Z. Guo, Z. Li, and W. Zhu (2013), Biomass accumulation and carbon sequestration in four different aged Casuarina equisetifolia coastal shelterbelt plantations in South China, *PloS One*, *8*(10), e77449.
664. Wang, G., D. Guan, M. R. Peart, Y. Chen, and Y. Peng (2014), Ecosystem carbon stocks of mangrove forest in Yingluo Bay, Guangdong Province of South China, *Forest Ecology & Management*, *310*(1), 539-546.
665. Wang, G., F. Ran, R. Chang, Y. Yang, J. Luo, and J. Fan (2014), Variations in the live biomass and carbon pools of Abies georgei along an elevation gradient on the Tibetan Plateau, China, *Forest Ecology & Management*, *329*, 255-263.
666. Wang, G. J., S. P. Wang, Y. B. Hao, and X. C. Cai (2005), Effect of grazing on the plant functional group diversity and community biomass and their relationship along a precipitation gradient in Inner Mongolia steppe, *Acta Ecologica Sinica*, *25*(7), 1649-1656. (in Chinese)
667. Wang, G. Z. (2013), Abovegroung biomass and carbon storage of *Populus* × *Xiaohei* of afforestation of different asexual reproduction [D], *Hebei: Agricultural University of Hebei*. (in Chinese)
668. Wang, H. (2010), Soil carbon sequestration and the related processes in four subtropical plantations in southern China [D], *Beijing: Chinese Academy of Forestry*. (in Chinese)
669. Wang, H., Y. Huang, S. L. Wang, and D. S. Zou (2010), Carbon and nitrogen storage under different forest ecosystems in mid-subtropical regions, *Chinese Journal of Eco-Agriculture*, *18*(3), 576-580. (in Chinese)
670. Wang, H., S. R. Liu, J. M. Mo, J. X. Wang, F. Makeschin, and M. Wolff (2010), Soil organic carbon stock and chemical composition in four plantations of indigenous tree species in subtropical China, *Ecological Research*, *25*(6), 1071-1079.
671. Wang, H., S. Liu, J. Wang, Z. Shi, L. Lu, J. Zeng, A. Ming, J. Tang, and H. Yu (2013), Effects of tree species mixture on soil organic carbon stocks and greenhouse gas fluxes in subtropical plantations in China, *Forest Ecology & Management*, *300*(4), 4-13.
672. Wang, H., R. Wang, Y. Yu, M. J. Mitchell, and L. Zhang (2011), Soil organic carbon of degraded wetlands treated with freshwater in the Yellow River Delta, China, *Journal of environmental management*, *92*(10), 2628-2633.
673. Wang, H. Q., S. Q. Wang, Y. Fang, and K. Li (2014), Estimation on carbon sequestration of aboveground part of Phragmites australis around Chongming island, *Wetland Science*, *12*(5), 539-543. (in Chinese)
674. Wang, J., Y. P. Chen, Y. Cao, J. Y. Zhou, and L. Hou (2012), Carbon concentration and carbon storage in different components of natural Quercuswutaishanica forest in Ziwuling of Loess Plateau, northwest China, *Chinese Journal of Ecology*, *31*(12), 3058-3063. (in Chinese)
675. Wang, J., C. Song, X. Wang, and Y. Song (2012), Changes in labile soil organic carbon fractions in wetland ecosystems along a latitudinal gradient in Northeast China, *Catena*, *96*, 83-89.
676. Wang, J. L. (2010), Preliminary reaserch of plant and soil carbon sequestration potential in alpine meadow of Qing Hai province [D], *Qinghai: Northwest institute of plateau biology, CAS*. (in Chinese)
677. Wang, L. (2009), Study of biomass and its models of main shrub community type in northwest Sichuan [D], *Sichuan: Sichuan Agricultural University*. (in Chinese)
678. Wang, L., J. Li, J. Li, and W. X. Bai (2014), Effects of tillage rotation and fertilization on soil aggregates and organic carbon content in corn field in Weibei Highland, *Chinese Journal of Applied Ecology*, *25*(3), 759-768. (in Chinese)
679. Wang, L., H. Liu, Y. Liu, J. Li, H. Shao, W. Wang, and C. Liang (2014), Soil characteristic comparison of fenced and grazed riparian floodplain wetlands in the typical steppe region of the Inner Mongolian plateau, China, *The Scientific World Journal*, *2014*(2),765907.
680. Wang, L., C. Yi, X. Xu, B. Schütt, K. Liu, and L. Zhou (2009), Soil properties in two soil profiles from terraces of the Nam Co Lake in Tibet, China, *Journal of Mountain Science*, *6*(4), 354-361.
681. Wang, L. L. (2011), Effects of land use changes on carbon releases and soil carbon storage in the Sanjiang Plain, Northeast China [D], *Jilin: Northeast Institute of Geography and Agroecology, CAS*. (in Chinese)
682. Wang, M., X. T. Liu, J. T. Zhang, X. J. Li, G. D. Wang, X. R. Lu, and X. Y. Li (2014), Spatio-temporal variations of soil respiration in five typical plant communities in the meadow steppe of the western Songnen plain, China, *Chinese Journal of Plant Ecology*, *38*(4), 396-404. (in Chinese)
683. Wang, M. H. (2012), Variations of soil organic carbon at four different land use types in a coastal areas in northern Jiangsu [D], *Jiangsu: Nanjing Forestry University*. (in Chinese)
684. Wang, M. X., Z. F. Zhu, F. Liu, and W. F. Tan (2012), Composition characteristics of soil organic carbon under land use change in Jianghan plain, Hubei province, *Research of Soil and Water Conservation*, *19*(6), 24-28. (in Chinese)
685. Wang, P. C., L. J. Xing, W. F. Xiao, Z. L. Huang, L. Pan, and L. X. Zeng (2009), Organic carbon density and storage of forest ecosystems in Three Gorges Reservoir area, *Acta Ecologica Sinica*, *29*(1), 97-107. (in Chinese)
686. Wang, Q., S. Wang, and X. Yu (2011), Decline of soil fertility during forest conversion of secondary forest to Chinese fir plantations in subtropical China, *Land Degradation & Development*, *22*(22), 444-452.
687. Wang, Q., S. Wang, and J. Zhang (2009), Assessing the effects of vegetation types on carbon storage fifteen years after reforestation on a Chinese fir site, *Forest Ecology & Management*, *258*(7), 1437-1441.
688. Wang, Q., L. Zhang, L. Li, Y. Bai, J. Cao, and X. Han (2010), Changes in carbon and nitrogen of Chernozem soil along a cultivation chronosequence in a semi-arid grassland, *European Journal of Soil Science*, *60.0*(6), 916-923.
689. Wang, Q. F., Y. M. Chen, Y. Cao, J. Cui, and T. Zhang (2014), Topsoil carbon sequestration characteristic and influencing factors for two grasslands in Loess hilly region, *Bulletin of Soil and Water Conservation*, *34*(1), 58-64. (in Chinese)
690. Wang, Q. J., S. X. Li, W. Y. Wang, and Z. C. Jing (2008), The despondences of carbon and nitrogen reserves in plants and soils to vegetations cover change on Kobresia pygmaea meadow of Yellow river and Yangtze river source region, *Acta Ecologica Sinica*, *28*(3), 885-894. (in Chinese)
691. Wang, Q. K. (2006), Fractions of active soil organic matter in Cunninghamia lanceolata plantations: characteristics, accumulation and mineralization [D], *Liaoning: Institute of Applied Ecology, CAS*. (in Chinese)
692. Wang, Q. K., and S. L. Wang (2007), Soil organic matter under different forest types in Southern China, *Geoderma*, *142*(3-4), 349-356.
693. Wang, Q. K., S. L. Wang, and Z. W. Feng (2006), Comparison of active soil organic carbon pool between Chinese fir plantations and evergreen broadleaved forests, *Journal of Beijing Forestry University*, *28*(5), 1-6. (in Chinese)
694. Wang, Q. Y., F. Li, D. Cui, L. G. Wang, and W. Y. Wang (2014), Carbon storage of four kinds of new varieties of Poplar plantations, *Protection Forest Science and Technology,* (3), 19-21. (in Chinese)
695. Wang, S. (2013), Carbon sink/source of different land use systems in Chongming agricultural park [D], *Jiangsu: Nanjing Forestry University*. (in Chinese)
696. Wang, S., J. Liu, G. Yu, Y. Pan, Q. Chen, K. Li, and J. Li (2004), Effects of land use change on the storage of soil organic carbon: a case study of the Qianyanzhou forest experimental station in China, *Climatic Change*, *67*(2-3), 247-255.
697. Wang, S., X. Liang, Q. Luo, F. Fan, Y. Chen, Z. Li, H. Sun, T. Dai, J. Wan, and X. Li (2012), Fertilization increases paddy soil organic carbon density, *Journal of Zhejiang University Science B*, *13*(4), 274-282.
698. Wang, S., Y. B. Wu, J. Chen, and X. Y. Ding (2014), Carbon balance of Brassica oleracea var. botrytis from Chongming ecological agriculture park in Shanghai, *Journal of Zhejiang Agricultral & Forestry University*, *31*(2), 190-195. (in Chinese)
699. Wang, S., X. Zhao, H. Qu, X. Zuo, J. Lian, X. Tang, and R. Powers (2011), Effects of shrub litter addition on dune soil microbial community in Horqin Sandy Land, Northern China, *Arid Land Research and Management*, *25*(3), 203-216.
700. Wang, S. F. (2011), Spatial variability and influencing factors of regional soil organic carbon and total nitrogen in the upstream watershed of Miyun Reservoir, North China [D], *Beijing: Research Center for Eco-Environmental Sciences, CAS*. (in Chinese)
701. Wang, S. F., X. K. Wang, and Z. Y. Ouyang (2011), Characteristics and influencing factors of soil organic carbon in upstream watershed of Miyun reservoir in north China, *Soils*, *43*(4), 515-524. (in Chinese)
702. Wang, S. J., Z. L. Cao, X. Y. Li, Z. Y. Liao, B. H. Hu, and J. Ni (2013), Spatio-temporal distributions of soil carbon and nitrogen under the four riparian zones in the Dianchi lake, *Journal of Nanjing Forestry University (Natural Sciences Edition)*, *37*(5), 55-59. (in Chinese)
703. Wang, S. X. (2011), Effects of fertilization on soil C, N & P pools and its involved enzyme activity in paddy wetland ecosystems [D], *Zhengjiang: Zhejiang University*. (in Chinese)
704. Wang, S. X., X. Q. Liang, Q. X. Luo, F. Fan, Y. X. Chen, Z. Z. Li, H. X. Sun, T. F. Dai, J. N. Wan, and X. J. Li (2012), Fertilization increases paddy soil organic carbon density, *Journal of Zhejiang University SCIENCE B, 13*(4), 274-282.
705. Wang, S. Z. (2013), Study on carbon storage dynamic characteristics of the second generation of Chinese fir plantation at different ages [D], *Hunan: Central South University of Forestry & Technology*. (in Chinese)
706. Wang, W., J. Sardans, C. Zeng, C. Zhong, Y. Li, and J. Peñuelas (2014), Responses of soil nutrient concentrations and stoichiometry to different human land uses in a subtropical tidal wetland, *Geoderma*, *232*, 459-470.
707. Wang, W., D. Su, L. Qiu, H. Wang, J. An, G. Zheng, and Y. Zu (2013), Concurrent changes in soil inorganic and organic carbon during the development of larch, Larix gmelinii, plantations and their effects on soil physicochemical properties, *Environmental Earth Sciences*, *69*(5), 1559-1570.
708. Wang, W., X. Xie, A. Chen, C. Yin, and W. Chen (2013), Effects of long-term fertilization on soil carbon, nitrogen, phosphorus and rice yield, *Journal of plant nutrition*, *36*(4), 551-561.
709. Wang, W., W. Zeng, W. Chen, H. Zeng, and J. Fang (2013), Soil respiration and organic carbon dynamics with grassland conversions to woodlands in temperate China, *PloS one*, *8*(8), 65-65.
710. Wang, W. J., B. T. Wang, Z. Lv, B. C. Ren, N. Wang, S. Nie, and Y. B. Cao (2013), Soil organic carbon reserve of different forests in Taiyue mountain, *Journal of Arid Land Resources and Environment*, *27*(1), 81-85. (in Chinese)
711. Wang, W. J., W. T. Zhang, J. An, H. M. Wang, and Y. G. Zu (2013), Variation of soil carbons and fertilities in Larch plantation land, clear-cut site and farmland in NE China, *Scientia Silvae Sinicae*, *49*(9), 79-88. (in Chinese)
712. Wang, W. Q., C. Wang, C. Tong, C. S. Zeng, R. X. Jia, and J. F. Huang (2012), Soil organic carbon along a salinity gradient in Phragmites australis marsh in the Minjiang River Estuary, *Wetland Science*, *10*(2), 164-169. (in Chinese)
713. Wang, W. X., Z. M. Shi, D. Luo, S. R. Liu, L. H. Luo, A. G. Ming, and H. L. Yu (2013), Carbon and nitrogen storage under different plantations in subtropical south China, *Acta Ecologica Sinica*, *33*(3), 925-933. (in Chinese)
714. Wang, X., S. Dong, B. Yang, Y. Li, and X. Su (2014), The effects of grassland degradation on plant diversity, primary productivity, and soil fertility in the alpine region of Asia's headwaters, *Environmental Monitoring & Assessment*, *186*(10), 6903-6917.
715. Wang, X., C. Song, X. Sun, J. Wang, X. Zhang, and R. Mao (2013), Soil carbon and nitrogen across wetland types in discontinuous permafrost zone of the Xiao Xing'an Mountains, northeastern China, *Catena*, *101*, 31-37.
716. Wang, X., M. Xu, J. Wang, W. Zhang, X. Yang, S. Huang, and H. Liu (2014), Fertilization enhancing carbon sequestration as carbonate in arid cropland: assessments of long-term experiments in northern China, *Plant and soil*, *380*(1-2), 89-100.
717. Wang, X., Y. F. Yao, F. C. Qing, G. Hao, and W. D. Chang (2014), Vertical distribution of soil organic carbon about plants in Aohan County, *Northern Horticulture*(4), 149-152. (in Chinese)
718. Wang, X. L., J. Y. Han, L. G. Xu, R. R. Wan, Y. W. Chen (2014), Soil characteristics in relation to vegetation communities in the wetlands of Poyang Lake, China, *Wetlands*, *34*(4), 829-839.
719. Wang, X. P., X. R. Li, H. L. Xiao, and Y. X. Pan (2006), Evolutionary characteristics of the artificially revegetated shrub ecosystem in the Tengger Desert, northern China, *Ecological Research*, *21*(3), 415-424.
720. Wang, X. F. (2010), Study on the carbon sinks under the different kinds of plantations in Loess Plateau [D], *Shanxi: Northwest Agriculture and Forestry University*. (in Chinese)
721. Wang, X. L., Y. Wang, H. H. Shi, S. T. Peng, L. P. Gong, and X. B. Qin (2013), Carbon storage of Pinus thunbergii and Robinia pseudoacacia plantations on Nanchangshan Island, Changdao County of Shandong Province, China, *Chinese Journal of Applied Ecology*, *24*(5), 1263-1268. (in Chinese)
722. Wang, X. L., L. G. Xu, X. Yao, L. Bai, and Q. Zhang (2010), Analysis on the soil microbial biomass in typical hygrophilous vegetation of Poyang Lake, *Acta Ecologica Sinica*, *30*(18), 5033-5042. (in Chinese)
723. Wang, X. S., C. D. Huang, and Y. J. Wang (2010), Soil organic carbon storage under different land use types at mountain forest-drought valley ecotone in the upper reaches of Minjiang river, *Research of Soil and Water Conservation*, *17*(4), 148-152. (in Chinese)
724. Wang, X. W., X. Z. Li, J. J. Lv, J. Sun, Z. M. Li, and Z. F. Wu (2010), Effects of temperature on the carbon mineralization of peat in the permafrost wetland in the Daxing'an mountains, *Quaternary Sciences*, *30*(3), 591-597. (in Chinese)
725. Wang, X. Y. (2011), Distribution of carbon storage for *Larix Olgensis* plantation of different stand ages [D], *Beijing: Beijing Forestry University*. (in Chinese)
726. Wang, X. Y., Y. J. Sun, and W. Ma (2011), Biomass and carbon storage distribution of different density in *Larix olgensis* plantation, *Journal of Fujian College of Forestry*, *31*(3), 221-226. (in Chinese)
727. Wang, Y. (2009), Research on the features of soil physical and chemical properties in Tuwei-river source area [D], *Shanxi: Northwest Agriculture and Forestry University*. (in Chinese)
728. Wang, Y. (2012), The study of characteristicis of soil organic carbon and aggregates in different degraded alpine meadow [D], *Jiangsu: Nanjing Agricultural University*. (in Chinese)
729. Wang, Y., and Y. T. G. T. Bao (2014), Species composition and the aboveground biomass of *Stipa glareosa* community in desert steppe, *Chinese Journal of Grassland*, *36*(3), 108-111. (in Chinese)
730. Wang, Y., J. S. Liu, G. P. Wang, and W. Su (2008), Effect of paddy field drainage on *Carex lasiocarpa* mire wetland, *Advances in Water Science*, *19*(2), 198-204. (in Chinese)
731. Wang, Y., B. Fu, Y. Lü, and L. Chen (2011), Effects of vegetation restoration on soil organic carbon sequestration at multiple scales in semi-arid Loess Plateau, China, *Catena*, *85*(1), 58-66.
732. Wang, Y., Y. Li, X. Ye, C. Yu, and X. Wang (2010), Profile storage of organic/inorganic carbon in soil: From forest to desert, *Science of the Total Environment*, *408*(8), 1925-1931.
733. Wang, Y., J. S. Liu, J. Wang, and C. Sun (2012), Effects of wetland reclamation on soil nutrient losses and reserves in Sanjiang Plain, Northeast China, *Journal of Integrative Agriculture*, *11*(3), 512-520.
734. Wang, Y. J., C. D. Huang, J. Zhang, W. Q. Yang, and X. S. Wang (2010), Species diversity, biomass and their relationship of shrubberies in an arid valley of the Minjiang river, *Arid Zone Research*, *27*(4), 567-572. (in Chinese)
735. Wang, Y. R. (2012), Evaluation on carbon sink of Cunninghamia lanceolata Vernicia montana mixed forest and its relationship with nutrient characteristics [D], *Fujian: Fujian Agriculture and Forestry University*. (in Chinese)
736. Wang, Z., B. M. Du, Y. J. Han, X. Cui, Q. Li, D. Huang, C. Y. Xue, and W. Sun (2014), Carbon storage of Ligustrum lucidum plantation in Shanghai out loop forest belt, *Chinese Journal of Ecology*, *33*(4), 910-914. (in Chinese)
737. Wang, Z., Y. J. Han, H. Z. Kang, D. Huang, C. Y. Xue, S. Yin, and C. J. Liu (2012), Carbon storage of main tree species plantations for water resources conservation in upper reaches of Huangpu River, Shanghai, *Chinese Journal of Ecology*, *31*(8), 1930-1935. (in Chinese)
738. Wang, Z., T. Luo, R. Li, Y. Tang, and M. Du (2013), Causes for the unimodal pattern of biomass and productivity in alpine grasslands along a large altitudinal gradient in semi-arid regions, *Journal of Vegetation Science*, *24*(1), 189-201.
739. Wang, Z., and Q. Wang (2013), Cultivating erect milkvetch (*Astragalus adsurgens Pall.*) (*Leguminosae*) improved soil properties in loess hilly and gullies in China, *Journal of Integrative Agriculture*, *12*(9), 1652-1658.
740. Wang, Z., X. Yun, Z. Wei, M. P. Schellenberg, Y. Wang, X. Yang, and X. Hou (2014), Responses of plant community and soil properties to inter-annual precipitation variability and grazing durations in a desert steppe in Inner Mongolia, *Journal of Integrative Agriculture*, *13*(6), 1171-1182.
741. Wang, Z. P., X. G. Han, S. X. Chang, B. Wang, Q. Yu, L. Y. Hou, and L. H. Li (2013), Soil organic and inorganic carbon contents under various land uses across a transect of continental steppes in Inner Mongolia, *Catena*, *109*, 110-117.
742. Wei, H. D., and B. Dong (2013), Spatio-temporal dynamics of soil microbial biomass carbon in Populus tremula plantations in the southeast of Shandong Province, *Ecology and Environmnet*, *22*(2), 233-238. (in Chinese)
743. Wei, J., J. Cheng, W. Li, and W. Liu (2012), Comparing the effect of naturally restored forest and grassland on carbon sequestration and its vertical distribution in the Chinese Loess Plateau, *PloS One*, *7*(7), e40123.
744. Wei, W. J. (2007), Forest carbon density and storage in Jiangxi and Dagangshan mountains [D], *Inner: Inner Mongolia Agricultural University*. (in Chinese)
745. Wei, W. J., W. Z. You, H. D. Zhang, G. Zhao, and L. L. Dong (2014), Soil organic carbon and its impact factors in original *Pinus kororaiensis* mixed forest in eastern Liaoning mountainous area, *Liaoning Forestry Science and Technology*(2), 4-8. (in Chinese)
746. Wei, X., M. Huang, M. Shao, L. Li, X. Zhang, and R. Horton (2013), Shrubs increase soil resources heterogeneity along semiarid grass slopes in the Loess Plateau, *Journal of arid environments*, *88*, 175-183.
747. Wei, X., Q. Li, Y. Liu, S. Liu, X. Guo, L. Zhang, D. Niu, and W. Zhang (2013), Restoring ecosystem carbon sequestration through afforestation: A sub-tropic restoration case study, *Forest Ecology & Management*, *300*, 60-67.
748. Wei, X., L. Qiu, M. Shao, X. Zhang, and W. J. Gale (2011), The accumulation of organic carbon in mineral soils by afforestation of abandoned farmland, *PloS One*, *7*(3), e32054.
749. Wei, X., M. Shao, X. Fu, and R. Horton (2010), Changes in soil organic carbon and total nitrogen after 28 years grassland afforestation: effects of tree species, slope position, and soil order, *Plant & Soil*, *331*(1-2), 165-179.
750. Wei, X., M. Shao, X. Fu, R. Horton, Y. Li, and X. Zhang (2009), Distribution of soil organic C, N and P in three adjacent land use patterns in the northern Loess Plateau, China, *Biogeochemistry*, *96*(1-3), 149-162.
751. Wei, X. R. (2007), Studies on the distribution and transport of micronutrients in soils of a watershed in the Loessial Gully region [D], *Shanxi: Institute of soil and water conservation, CAS and MWR*. (in Chinese)
752. Wei, Y. C., Z. Y. Ouyang, H. Miao, X. K. Wang, H. Zheng, and L. J. Jiang (2007), Spatial heterogeneity of soil properties in Jianfengling nature reserve, *Chinese Journal of Ecology*, *26*(2), 197-203. (in Chinese)
753. Wei, Y. H., X. Zhao, Y. L. Zhai, E. P. Zhang, B. Chen, and H. L. Zhang (2013), Effects of tillages on soil organic carbon sequestration in north China plain, *Transactions of the Chinese Society of Agricultural Engineering*, *29*(17), 87-95. (in Chinese)
754. Wei, Y. M. (2010), Research on biomass and carbon stock for large-scale implementation of Poplar plantation in desert environment [D], *Xinjiang: Xinjiang University*. (in Chinese)
755. Wei, Y. W., Y. R. Su, X. B. Chen, and X. Y. He (2010), Effects of human disturbance on profile distribution of soil organic C, total N, total P and microbial biomass in karst region of northwest Guangxi, *Journal of Soil and Water Conservation*, *24*(3), 164-169. (in Chinese)
756. Wen, H., D. Niu, F. Hua, and K. Jian (2013), Experimental investigation on soil carbon, nitrogen, and their components under grazing and livestock exclusion in steppe and desert steppe grasslands, Northwestern China, *Environmental Earth Sciences*, *70*(7), 3131-3141.
757. Wen, J., H. K. Zhou, B. Q. Yao, Y. K. Li, X. Q. Zhao, Z. Chen, L. Y. Lian, and K. X. Guo (2014), Characteristics of soil respiration in different degraded alpine grassland in the source region of Three-River, *Chinese Journal of Plant Ecology*, *38*(2), 209-218. (in Chinese)
758. Wen, L., S. Dong, Y. Li, X. Wang, X. Li, J. Shi, and Q. Dong (2013), The impact of land degradation on the C pools in alpine grasslands of the Qinghai-Tibet Plateau, *Plant & Soil*, *368*(1-2), 329-340.
759. Wen, L., P. F. Lei, and L. Dai (2014), Storages and distribution characteristics of soil organic carbon and nitrogen in pure Cinnamomum camphora forests at different stand ages, *Journal of Central South Forestry University*, *34*(6), 106-111. (in Chinese)
760. Wen, S. Z., D. L. Tian, L. L. Yang, and X. Fang (2010), Carbon density, carbon stock and carbon sequestration in Alnus cremastogyne plantation, *Scientia Silvae Sinicae*, *46*(6), 15-21. (in Chinese)
761. Wu, G. L., Z. H. Liu, L. Zhang, J. M. Chen, and T. M. Hu (2010), Long-term fencing improved soil properties and soil organic carbon storage in an alpine swamp meadow of western China, *Plant and Soil*, *332*(1-2), 331-337.
762. Wu, G. L., G. H. Ren, Q. M. Dong, J. J. Shi, and Y. L. Wang (2014), Above-and belowground response along degradation gradient in an alpine grassland of the Qinghai-Tibetan Plateau, *Clean-Soil, Air, Water*, *42*(3), 319-323.
763. Wu, H. F., and Z. J. Wang (2014), Analysis on spatial and temporal dynamic of biomass of Phragmitesaustralis in Yeya Lake wetland, *Journal of Capital Normal University: Natural Science Edition*(6), 51-55. (in Chinese)
764. Wu, J. (2011), Carbon accumulation in paddy ecosystems in subtropical China: evidence from landscape studies, *European Journal of Soil Science*, *62*(1), 29-34.
765. Wu, J. G., X. Q. Zhang, and D. Y. Xu (2004), Impact of land use change on soil carbon storage, *Chinese Journal of Applied Ecology*, *15*(4), 593-599. (in Chinese)
766. Wu, J. J., Z. J. Yang, F. J. Weng, X. F. Liu, C. Q. Chen, W. S. Lin, X. H. Wang, and T. Chen (2014), Comparison of soil respiration in natural Castanopsis carlesii forest and plantation forest, *Environmental Science*, *35*(6), 2426-2432. (in Chinese)
767. Wu, J. P., Z. F. Liu, Y. X. Sun, L. X. Zhou, Y. B. Lin, and S. L. Fu (2013), Introduced Eucalyptus *upophylla* plantations change the composition of the soil microbial community in subtropical China, *Land Degradation & Development*, *24*(4), 400-406.
768. Wu, L., N. He, Y. Wang, and X. Han (2008), Storage and dynamics of carbon and nitrogen in soil after grazing exclusion in *Leymus chinensis* grasslands of northern China, *Journal of Environmental Quality*, *37*(2), 663-668.
769. Wu, L., and X. S. Zhang (2006), Characters of forage resources and the development of pastoral industry in the farming-pastoral zone of the Songnen plain, *Acta Ecologica Sinica*, *26*(2), 601-609. (in Chinese)
770. Wu, M. (2009), Characteristics of carbon stocks and stability of soil organic carbon under three artificial forest types in mid-subtropical [D], *Jiangsu: Nanjing Agricultural University*. (in Chinese)
771. Wu, P., J. Chen, Y. C. Cui, F. J. Ding, and J. Zhu (2012), Study of soil organic carbon of major successional communities in Maolan nature reserve of karst, *Journal of Central South Forestry University*, *32*(12), 181-186. (in Chinese)
772. Wu, P. F., B. Zhu, S. R. Liu, and X. G. Wang (2008), Carbon storage and its allocation in mixed alder-cypress plantations at different age stages, *Chinese Journal of Applied Ecology*, *19*(7), 1419-1424. (in Chinese)
773. Wu, Q., B. Yao, R. X. Xing, L. L. Zhu, and Q. W. Hu (2012), Distribution pattern of soil organic carbon in Poyang Lake wetland and related affecting factors, *Chinese Journal of Ecology*, *31*(2), 313-318. (in Chinese)
774. Wu, T., C. H. Peng, D. L. Tian, and W. D. Yan (2012), Spatial distribution of carbon storage in a 13-year-old *Pinus massoniana* forest ecosystem in Changsha City, China, *Acta Ecologica Sinica*, *32*(13), 4034-4042. (in Chinese)
775. Wu, X., Z. Li, B. Fu, F. Lu, D. Wang, H. Liu, and G. Liu (2014), Effects of grazing exclusion on soil carbon and nitrogen storage in semi-arid grassland in Inner Mongolia, China, *Chinese Geographical Science*, *24*(4), 479-487.
776. Wu, X., Z. Yao, N. Brüggemann, Z. Y. Shen, B. Wolf, M. Dannenmann, X. Zheng, and K. Butterbach-Bahl (2010), Effects of soil moisture and temperature on CO_2_ and CH_4_ soil-atmosphere exchange of various land use/cover types in a semi-arid grassland in Inner Mongolia, China, *Soil Biology & Biochemistry*, *42*(5), 773-787.
777. Wu, X. C. (2009), Research on the productivity and carbon density of natural Poplar and willow forest in Ergis river, Xinjiang [D], *Inner: Inner Mongolia Agricultural University*. (in Chinese)
778. Wu, X. G., J. P. Guo, X. Y. Li, and X. P. Tian (2011), Soil organic carbon storage and profile inventory in the different vegetation types of Luya mountain, *Acta Ecologica Sinica*, *31*(11), 3009-3019. (in Chinese)
779. Wu, X. G., J. P. Guo, X. P. Tian, and X. Y. Yang (2014), Distribution characteristics of soil organic carbon and total nitrogen along elevation gradients in Luya mountain, *Ecology and Environmnet*, *23*(1), 50-57. (in Chinese)
780. Wu, Y. (2012), The effect of tillage systems on organic carbon fractions in a purple paddy soil [D], *Chongqin: Southwest University*. (in Chinese)
781. Wu, Y., J. Wu, Y. Deng, H. Tan, Y. Du, S. Gu, Y. Tang, and X. Cui (2011), Comprehensive assessments of root biomass and production in a Kobresia humilis meadow on the Qinghai-Tibetan Plateau, *Plant & Soil*, *338*(1), 497-510.
782. Wu, Y. C., Z. C. Li, C. F. Cheng, R. J. Liu, B. Wang, and L. T. Geri (2013), Effects of understory removal on forest carbon storage in Cinnamomum camphora plantation ecosystem, *Chinese Journal of Plant Ecology*, *37*(2), 142-149. (in Chinese)
783. Xian, Y., G. T. Meng, Y. N. Zhao, and M. Wang (2014), Vertical distribution patterns of soil organic matter and total nitrogen contents in different altitudes of eastern slope of Gaoligong mountain, *Acta Agriculturae Jiangxi*, *26*(5), 6-10. (in Chinese)
784. Xiang, Y. Z. (2012), Spatial and temporal pattern of biomass and carbon storage of *eucalypt* plantation ecosystems in Hainan [D], *Beijing: Chinese Academy of Forestry*. (in Chinese)
785. Xiao, C., I. A. Janssens, P. Liu, Z. Zhou, and O. J. Sun (2007), Irrigation and enhanced soil carbon input effects on below-ground carbon cycling in semiarid temperate grasslands, *New Phytologist*, *174*(4), 835-846.
786. Xiao, C. B., H. Wang, K. F. Fan, B. Xavier, Y. J. Han, H. Z. Kang, and C. J. Liu (2010), Carbon storage of Metasequoia glyptostroboides plantation ecosystems at different age stages in Chongming island, east China, *Journal of Shanghai Jiaotong University: Agricultural Science*, *28*(1), 30-34. (in Chinese)
787. Xiao, C. W., I. A. Janssens, W. G. Sang, R. Z. Wang, Z. Q. Xie, Z. Q. Pei, and Y. Yi (2010), Belowground carbon pools and dynamics in China’s warm temperate and sub-tropical deciduous forests, *Biogeosciences*, *7*(1), 275-287.
788. Xiao, D. R., K. Tian, and L. Q. Zhang (2008), Relationship between plant diversity and soil fertility in Napahai wetland of Northwestern Yunnan Plateau, *Acta Ecologica Sinica*, *28*(7), 3116-3124. (in Chinese)
789. Xiao, F., and W. Huang (2012), Soil carbon, nitrogen and microbial biomass dynamics of subalpine *Abies fabri* forest in Gongga Mountain, Southwest China, *African Journal of Microbiology Research*, *6*(32), 6091-6098.
790. Xiao, F. M. (2007), A study on the characteristics of carbon balance in Phyllostachys edulis plantation ecosystem [D], *Beijing: Chinese Academy of Forestry*. (in Chinese)
791. Xiao, S. S. (2007), Soil respiration and carbon balance study of Casuarina equisetifolia plantation ecosystem on coastal sand [D], *Fujian: Fujian Agriculture and Forestry University*. (in Chinese)
792. Xiao, X. P., N. P. Song, T. T. Xie, and K. Fang (2013), Formation mechanism and community characteristics of fenced grassland in desert steppe, *Acta Prataculturae Sinica*, *22*(6), 321-327. (in Chinese)
793. Xiao, Y., L. N. Shang, Z. G. Huang, W. G. Zhang, Z. S. Xue, Z. S. Zhang, and X. G. Lv (2014), Ecological stoichiometry characteristics of soil carbon, nitrogen and phosphorus in mountain swamps of eastern Jilin Province, *Scientia Geographica Sinica*, *34*(8), 994-1001. (in Chinese)
794. Xiao, Y. F. (2013), Soil organic carbon spatial distribution and the analysis of the influencing factors in Mangshsan [D], *Hunan: Central South University of Forestry & Technology*. (in Chinese)
795. Xiao, Z. W., L. J. Wang, J. M. Mao, X. Z. Zhu, X. L. Wang, L. Zheng, and J. W. Tang (2012), Carbon storage of different tree-tea agroforestry systems in Xishuangbanna,Yunnan province of southwest China, *Chinese Journal of Ecology*, *31*(7), 1617-1625. (in Chinese)
796. Xiao, Z. W., X. H. Wang, L. Zheng, X. L. Wang, L. H. Gao, and J. W. Tang (2014), Biomass and its allocation pattern of monoculture and mixed rubber-tree plantations in Xishuangbanna, *Journal of Central South Forestry University*, *34*(2), 108-116. (in Chinese)
797. Xie, C. Y., T. K. Xu, S. X. Chen, C. Y. Hu, and B. Chen (2011), Effect of phytocoenosis on soil carbon nitrogen and phosphorus distribution in Hangzhou Xixi wetland, *Journal of Hangzhou Normal University: natural science edition*, *10*(6), 501-505. (in Chinese)
798. Xie, J., J. Guo, Z. Yang, Z. Huang, G. Chen, and Y. Yang (2013), Rapid accumulation of carbon on severely eroded red soils through afforestation in subtropical China, *Forest Ecology & Management*, *300*, 53-59.
799. Xie, L. J. (2011), Distribution characteristics of soil organic carbon and total nitrogen under long-term fertilization in typical arable land soil of China [D], *Beijing: Chinese Academy of Agricultural Sciences*. (in Chinese)
800. Xie, W. X., K. J. Zhu, Y. Q. Cui, H. N. Du, and J. L. Chen (2014), Spatial distribution of soil carbon and nitrogen in Jiaozhou Bay estuarine wetlands, *Acta Prataculturae Sinica*, *23*(6), 54-60. (in Chinese)
801. Xie, X. M., and M. Shiyomi (2011), Aboveground plant mass and mass available to grazing goats in a mountainous shrubland in subtropical China, *Grassland Science*, *57*(3), 119-126.
802. Xin, K., K. Yan, Z. Li, J. L. Hu, and Y. H. Qiu (2014), Distribution of soil organic carbon in Mangrove wetlands of Hainan island and its influencing factors, *Acta Pedologica Sinica*, *51*(5), 1078-1086. (in Chinese)
803. Xin, W. J., Y. Q. Su, M. Q. Zhu, R. R. Hu, Y. Liu, and S. Huang (2014), Distribution characteristics of soil organic carbon of different forests in loess plateau of Qianyang county, *Journal of Central South Forestry University*, *34*(5), 66-69. (in Chinese)
804. Xiong, D., P. Shi, Y. Sun, J. Wu, and X. Zhang (2014), Effects of grazing exclusion on plant productivity and soil carbon, nitrogen storage in alpine meadows in Northern Tibet, China, *Chinese Geographical Science*, *24*(4), 488-498.
805. Xiong, X. G., and X. G. Han (2006), Dynamics of the small-scale heterogeneity of the soil carbon and nitrogen resources associated with Caragana microphylla in Inner Mongolia degraded steppe, *Acta Ecologica Sinica*, *26*(2), 483-488. (in Chinese)
806. Xu, G. L., H. J. Zhang, X. H. Lv, J. H. Cheng, S. C. Du, Q. K. Li, H. W. Wei, X. Zhang, and X. J. Tian (2014), The Soil organic carbon storage study under five plantations in the simian mountain of Chongqing, *Ecology and Environmnet*, *23*(2), 211-216. (in Chinese)
807. Xu, H. H. (2010), Study on vertical distribution of soil organic carbon and its fractions in Ebinur lake wetland [D], *Fujian: Fujian Normal University*. (in Chinese)
808. Xu, H. H., C. S. Zeng, W. Q. Wang, and J. H. Zhai (2010), Study on vertical distribution and the influencing factors of soil organic carbon in Ebinur lake wetland, *Journal of Fujian Normal University: natural science edition*, *26*(5), 86-91. (in Chinese)
809. Xu, H. T., L. Q. Wang, J. Shen, G. H. Ji, and F. B. Zhao (2011), Plant and soil organic carbon pools in a constructed wetland of Shatianhu River, *Chinese Journal of Ecology*, *30*(6), 1083-1090. (in Chinese)
810. Xu, J. L. (2009), Study on Larix Biomass model and biomass estimation in Dongzhelenghe [D], *Beijing: Beijing Forestry University*. (in Chinese)
811. Xu, M. H., and X. Xue (2013), A research on summer vegetation characteristics and short-time responses to experimental warming of alpine meadow in the Qinghai-Tibetan Plateau, *Acta Ecologica Sinica*, *33*(7), 2071-2083. (in Chinese)
812. Xu, M. Y., P. G. Li, F. Xie, D. Huang, F. Wang, Z. M. Yan, and K. Wang (2011), Response of soil organic carbon density to landuse types and management practices change in agro-pastoral zone, *Transactions of the Chinese Society of Agricultural Engineering*, *27*(7), 320-325. (in Chinese)
813. Xu, Q. F., P. K. Jiang, and Q. Shen (2005), Comparison of organic carbon pool of soil in bush and broad-leaved forests, *Journal of Beijing Forestry University*, *27*(2), 18-22. (in Chinese)
814. Xu, Q. X. (2013), Effect of thinning on soil physicochemical property and carbon storage of the natural Larix gmelinii forest in Great Xing'an mountains [D], *Heilongjiang: Northeast Forestry University*. (in Chinese)
815. Xu, S. Q., M. Y. Zhang, H. L. Zhang, F. Chen, G. L. Yang, and X. P. Xiao (2013), Soil organic carbon stocks as affected by tillage systems in a double-cropped rice field, *Pedosphere*, *23*(5), 696-704.
816. Xu, W. (2011), Research on carbon density and carbon storage of Pinus massoniana in Jinghuai hilly region in China [D], *Jiangsu: Nanjing Forestry University*. (in Chinese)
817. Xu, W. Q., G. P. Luo, and X. Chen (2006), Soil properties under shrubs in arid area of oasis-desert transition belt, *Chinese Journal of Applied Ecology*, *17*(4), 583-586. (in Chinese)
818. Xu, X. M. (2012), Study on carbon density of Populus tomentosa plantations at different ages on Loess Plateau [D], *Shanxi: Northwest Agriculture and Forestry University*. (in Chinese)
819. Xu, X. M., Y. Liu, L. L. Zhang, and Y. Q. Su (2012), Carbon density in forest ecosystem of old Populus tomentosa plantation in Loess Plateau, *Shaanxi Forest Science and Technology*(6), 1-5, 9. (in Chinese)
820. Xu, X. W., X. Q. He, P. Cui, and M. Mao (2014), Impacts of human disturbances on organic carbon density of soil in fresh water wetlands along Yangzi River in Anhui, *Journal of Chizhou Teachers College*, *28*(3), 1-5. (in Chinese)
821. Xue, L., Y. Xue, G. W. Lie, L. H. Ye, and X. L. Huang (2012), Soil organic carbon storage on different slope positions in Cunninghamia Lanceolata stands, *Bulletin of Soil and Water Conservation*, *32*(6), 43-46. (in Chinese)
822. Xue, X. J. (2009), Response of simulated climate change on the changes of soil and plant carbon and nitrogen with different altitude in alpine meadow [D], *Qinghai: Northwest institute of plateau biology, CAS*. (in Chinese)
823. Yan, D. F. (2012), Collaborative mechanisms of vegetation and soil succession under different vegetation restoration measures [D], *Henan: Henan Agricultural University*. (in Chinese)
824. Yan, D. R., C. G. Zhao, K. Qi, and J. Y. Guo (2007), Study on soil fertility of shrub-coppices of Caragana microphylla *Journal of Inner Mongolia Forestry Science & Technology*, *33*(3), 1-4. (in Chinese)
825. Yan, E. R., X. H. Wang, and X. Y. Chen (2007), Impacts of evergreen broad-leaved forest, degradation on soil nutrients and carbon pools in Tiantong, Zhejiang province, *Acta Ecologica Sinica*, *27*(4), 1646-1655. (in Chinese)
826. Yan, G. (2014), A study on saltmarsh vegetation biomass and carbon distribution in Chongming Dongtan wetlands [D], *Shanghai: East China Normal University*. (in Chinese)
827. Yan, G., Z. M. Ge, and L. Q. Zhang (2014), Distribution of soil carbon storage in different saltmarsh plant communities in Chongming Dongtan wetland, *Chinese Journal of Applied Ecology*, *25*(1), 85-91. (in Chinese)
828. Yan, P., and X. C. Feng (2006), Spatial distribution and carbon storage in primitive broadleaved korean Pine forests, *Journal of Northeast Forestry University*, *34*(5), 23-25. (in Chinese)
829. Yan, R. R., X. P. Xin, X. Wang, Y. C. Yan, Y. Deng, and G. X. Yang (2014), The change of soil carbon and nitrogen under different grazing gradients in Hulunber meadow steppe, *Acta Ecologica Sinica*, *34*(6), 1587-1595. (in Chinese)
830. Yan, Y., S. Liu, and W. Zhou (2006), Dynamic of grassland biomass in different degenerative stages, *Wuhan University Journal of Natural Sciences*, *11*(4), 958-962.
831. Yan, Y., J. G. Zhang, J. H. Zhang, J. R. Fan, and H. X. Li (2005), The belowground biomass in alpine grassland in Nakchu Prefecture of Tibet, *Acta Ecologica Sinica*, *25*(11), 2818-2823. (in Chinese)
832. Yang, C. D., R. J. Long, X. R. Chen, C. L. Xu, and J. M. Wang (2008), Characteristics of carbon, nitrogen and phosphorus density in top soil under different alpine grasslands on the Eastern Qilian Mountains, *Chinese Journal of Grassland*, *30*(1), 1-5. (in Chinese)
833. Yang, D., W. H. Xiang, X. Fang, G. W. Fan, Y. Q. Xu, L. Wen, and L. M. Zou (2014), Spatial heterogeneity of soil organic carbon and total nitrogen concentrations in a *Lithocarpus glaber-Cyclobalanopsis glauca* evergreen broadleaved forest, *Acta Ecologica Sinica*, *34*(12), 3452-3462. (in Chinese)
834. Yang, F. Y., X. J. Li, and X. T. Liu (2012), Use patterns of ecological agriculture and biological carbon sinking extension in marsh wetland, *Transactions of the Chinese Society of Agricultural Engineering*, *28*(19), 156-162. (in Chinese)
835. Yang, G. X. (2012), Carbon stock and its allocation study on mixed broadleaf-coniferforest in Baotianman nature reserve [D], *Henan: Henan Agricultural University*. (in Chinese)
836. Yang, H., X. Li, Z. Wang, R. Jia, L. Liu, Y. Chen, Y. Wei, Y. Gao, and L. Gang (2014), Carbon sequestration capacity of shifting sand dune after establishing new vegetation in the Tengger Desert, northern China, *Science of the Total Environment*, *478*(8), 1-11.
837. Yang, H. X., Y. J. He, Y. Zheng, W. Wang, P. Wang, and B. Yao (2013), Response of soil physicochemical properties to different management modes in natural secondary coniferous forests, *Journal of Northeast Forestry University*, *41*(3), 63-68. (in Chinese)
838. Yang, J. (2013), Effect of forest closed on biomass and carbon storage in several kinds of stands [D], *Hubei: Huazhong Agricultural University*. (in Chinese)
839. Yang, J., M. Rui, and J. Liu (2009), Variations in soil properties and their effect on subsurface biomass distribution in four alpine meadows of the hinterland of the Tibetan Plateau of China, *Environmental Geology*, *57*(8), 1881-1891.
840. Yang, J. J. (2012), Carbon storage of Pinus tabulaeformis plantation at different stand ages in Pingquan of Hebei province [D], *Beijing: Beijing Forestry University*. (in Chinese)
841. Yang, J. Y., and C. K. Wang (2005), Soil carbon storage and flux of temperate forest ecosystems in northeastern China, *Acta Ecologica Sinica*, *25*(11), 2875-2882. (in Chinese)
842. Yang, L., J. Pan, Y. Shao, J. M. Chen, W. M. Ju, X. Shi, and S. Yuan (2007), Soil organic carbon decomposition and carbon pools in temperate and sub-tropical forests in China, *Journal of Environmental Management*, *85*(3), 690-695.
843. Yang, L. L., F. S. Zhang, R. Z. Mao, X. T. Ju, X. B. Cai, and Y. H. Lu (2008), Conversion of natural ecosystems to cropland increases the soil net nitrogen mineralization and nitrification in Tibet, *Pedosphere*, *18*(6), 699-706.
844. Yang, S. J., T. Li, Y. M. Gan, Y. Wang, L. Ji, Z. Q. Song, and T. Liu (2014), Impact of different use patterns and degrees of grassland use on vegetation carbon storage in the Aba grassland pastoral area, *Acta Prataculturae Sinica*, *23*(3), 325-332. (in Chinese)
845. Yang, T. H. (2005), Study on aboveground biomass and distribution pattern of evergreen broad-leaved forest in Tiantong national forest park, Zhejiang province [D], *Shanghai: East China Normal University*. (in Chinese)
846. Yang, W. Y. (2011), Study on the soil respiration character and soil labile organic carbon of four kinds of wetland environment in Hangzhou Bay [D], *Chongqin: Southwest University*. (in Chinese)
847. Yang, X. F. (2011), Research on the carbon storage and the environment response of Poplar plantation in Xiping of Henan province [D], *Beijing: Beijing Forestry University*. (in Chinese)
848. Yang, X. J. (2012), Tibetan grasslands biomass and its environmental control factors [D], *Beijing: Institute of Geographical Sciences and Natural Resources Research, CAS*. (in Chinese)
849. Yang, X. J., S. Z. Tong, X. Y. Li, J. Y. Ynag, and T. Liu (2012), Response of growth feature of reed conununities in Zhalong wetland to the change of water depth gradient, *Journal of Northeast Forestry University*, *40*(12), 67-70. (in Chinese)
850. Yang, X. M. (2010), Carbon storage and density of natural Pinus shekannesis forest in Ziwuling area [D], *Shanxi: Institute of soil and water conservation, CAS and MWR*. (in Chinese)
851. Yang, X. M., J. M. Cheng, L. Meng, and J. J. Han (2010), Carbon storage and density of forests in Ziwuling area of Loess Plateau, *Journal of Soil and Water Conservation*, *24*(6), 123-126. (in Chinese)
852. Yang, X. X., F. Ren, H. K. Zhou, and J. S. He (2014), Responses of plant community biomass to nitrogen and phosphorus additions in an alpine meadow on the Qinghai-Xizang Plateau, *Chinese Journal of Plant Ecology*, *38*(2), 159-166. (in Chinese)
853. Yang, Y., B. R. Liu, D. P. Zhai, and X. G. Yang (2014), Effect of row distance of artificial Caragana korshinskii shrub on spatial distribution of soil organic carbon in desert steppe, *Journal of Soil and Water Conservation*, *28*(1), 141-146. (in Chinese)
854. Yang, Y., D. C. Niu, H. Y. Wen, B. L. Zhang, Q. Dong, J. L. Chen, and H. Fu (2012), Responses of soil particulate organic carbon and nitrogen along an altitudinal gradient on the Helan Mountain, Inner Mongolia, *Acta Prataculturae Sinica*, *21*(3), 54-60. (in Chinese)
855. Yang, Y., F. Ran, G. X. Wang, W. Z. Zhu, Y. Yang, and P. Zhou (2013), Biomass model and carbon storage of Pinus yunnanensis on Tibet Plateau of China, *Chinese Journal of Ecology*, *32*(7), 1674-1682. (in Chinese)
856. Yang, Y., G. Wang, H. Shen, Y. Yang, H. Cui, and Q. Liu (2014), Dynamics of carbon and nitrogen accumulation and C:N stoichiometry in a deciduous broadleaf forest of deglaciated terrain in the eastern Tibetan Plateau, *Forest Ecology & Management*, *312*(312), 10-18.
857. Yang, Y. H., J. Y. Fang, W. H. Ma, P. Smith, A. Mohammat, S. P. Wang, and W. Wang (2010), Soil carbon stock and its changes in northern China's grasslands from 1980s to 2000s. *Global Change Biology, 16*(), 3036-3047.
858. Yang, Y., J. Xie, H. Sheng, G. Chen, X. Li, and Z. Yang (2009), The impact of land use/cover change on storage and quality of soil organic carbon in midsubtropical mountainous area of southern China, *Journal of Geographical Sciences*, *19*(1), 49-57.
859. Yang, Y. J., Y. M. Chen, and Y. Cao (2014), Carbon density and distribution of Pinus tabulaeformis plantation ecosystem in Hilly Loess Plateau, *Acta Ecologica Sinica*, *34*(8), 2128-2136. (in Chinese)
860. Yang, Z. J. (2007), Carbon sequestration and balance in pure plantations of Cunninghamia lanceolata and Schima superba [D], *Fujian: Fujian Agriculture and Forestry University*. (in Chinese)
861. Yao, T., G. Wang, D. G. Zhang, and R. J. Long (2006), Temporal changes of grassland vegetation, soil and soil microbial population in the Tianzhu alpine region, *Acta Ecologica Sinica*, *26*(6), 1926-1932. (in Chinese)
862. Ye, C., X. Cheng, Y. Zhang, Z. Wang, and Q. Zhang (2012), Soil nitrogen dynamics following short-term revegetation in the water level fluctuation zone of the Three Gorges Reservoir, China, *Ecological Engineering*, *38*(1), 37-44.
863. Yi, M. B. (2008), Study on soil organic carbon storage under principal vegetations and relationship with environment factors in the Dagou Valley [D], *Chongqin: Southwest University*. (in Chinese)
864. Yin, S. B., Q. Yang, and X. G. Lv (2006), Distribution and accumulation of organic carbon in typical annular wetlands of the Sanjiang Plain, *Chinese Journal of Soil Science*, *37*(4), 659-661. (in Chinese)
865. You, M. Y., H. B. Li, and X. Z. Han (2010), Land use change and long term fertilization impact on SOC density in black soil of northeast China, *Journal of Soil and Water Conservation*, *24*(2), 155-159. (in Chinese)
866. You, W. Z., C. F. Huo, Z. K. Xing, G. Zhao, H. D. Zhang, W. J. Wei, and T. W. Yan (2011), Biomass and net primary productivity of Larix olgensis plantation in Bingla mountains,northeast China, *Journal of Shenyang Agricultural University*, *42*(5), 565-569. (in Chinese)
867. Yu, H. Q., G. M. He, F. Zhang, and G. P. Chen (2012), Research on the forest carbon storage in Fengjiayu township of Miyun county in Beijing, *Forest Resources Management*(1), 37-41, 47. (in Chinese)
868. Yu, J., H. Dong, Y. Li, H. Wu, B. Guan, Y. Gao, D. Zhou, and Y. Wang (2014), Spatiotemporal distribution characteristics of soil organic carbon in newborn coastal wetlands of the Yellow River Delta estuary, *CLEAN–Soil, Air, Water*, *42*(3), 311-318.
869. Yu, J., J. Liu, F. X. Meixner, J. Wang, Y. Gao, Y. Wang, X. Qi, and X. Chen (2010), Estimating net primary productivity and nutrient stock in plant in freshwater marsh, northeastern China, *CLEAN-Soil, Air, Water*, *38*(11), 1080-1086.
870. Yu, J., X. Chen, Z. Sun, W. Xie, P. Mao, C. Wu, H. Dong, X. Mu, Y. Li, B. Guan, K. Shan (2010), The spatial distribution characteristics of soil nutrients in new-born coastal wetland in the Yellow River delta, *Acta Scientiae Circumstantiae*, *30*(4), 855-861. (in Chinese)
871. Yu, P., Q. Li, H. Jia, W. Zheng, M. Wang, and D. Zhou (2013), Carbon stocks and storage potential as affected by vegetation in the Songnen grassland of northeast China, *Quaternary International*, *306*(450), 114-120.
872. Yu, S., D. Wang, W. Dai, and P. Li (2014), Soil carbon budget in different-aged Chinese fir plantations in south China, *Journal of Forestry Research*, *25*(3), 621-626.
873. Yu, S. Y., J. Guo, G. Chen, J. Xie, G. Ren, L. Zhen, and J. Zhao (2005), Carbon and nitrogen pools in Chinese fir and evergreen broadleaved forests and changes associated with felling and burning in mid-subtropical China, *Forest Ecology & Management*, *216*(1-3), 216-226.
874. Yu, X. L., and L. S. Xu (2012), Changes of soil organic carbon and complex iron in Momoge wetland, *Journal of Northeast Normal University: Natural Science Edition*, *44*(3), 118-123. (in Chinese)
875. Yu, Y. (2014), Estimation of soil density and storage in Honghe nature reserve, *Modern Economic Information*(6), 393-393. (in Chinese)
876. Yu, Y. X. (2010), Carbon density of forest vegetation and spatial distribution in south Lvliang mountain [D], *Shanxi: Shanxi University*. (in Chinese)
877. Yuan, H. W. (2007), Distribution characteristics and storage of soil organic carbon and nitrogen in typical profiles in red soil hilly and karst regions [D], *Hunan: Institute of Subtropical Agriculture, CAS*. (in Chinese)
878. Yuan, S. F., Y. N. Chen, W. H. Li, J. Z. Liu, L. H. Meng, and L. H. Zhang (2006), Analysis of the aboveground biomass and spatial distribution of shrubs in the lower reaches of Tarim river, Xinjiang, China, *Acta Ecologica Sinica*, *26*(6), 1818-1824. (in Chinese)
879. Zeng, C. S., C. Q. Zhong, C. Tong, and Z. Z. Liu (2008), Impacts of LUCC on soil organic carbon contents in wetland of Minjiang river, *Journal of Soil and Water Conservation*, *22*(5), 125-129. (in Chinese)
880. Zeng, D. H., Y. L. Hu, S. X. Chang, and Z. P. Fan (2008), Land cover change effects on soil chemical and biological properties after planting Mongolian pine (*Pinus sylvestris var. mongolica*) in sandy lands in Keerqin, northeastern China, *Plant & Soil*, *317*(1), 121-133.
881. Zeng, W. B., Q. Deng, Q. F. Zhang, and X. L. Cheng (2013), Changes in soil carbon and nitrogen concentration under land use change in Danjiangkou reservoir area, China, *Soils*, *45*(3), 385-391. (in Chinese)
882. Zeng, X., W. Zhang, J. Cao, X. Liu, H. Shen, and X. Zhao (2014), Changes in soil organic carbon, nitrogen, phosphorus, and bulk density after afforestation of the “Beijing-Tianjin Sandstorm Source Control” program in China, *Catena*, *118*, 186-194.
883. Zeng, X., W. Zhang, X. Liu, J. Cao, H. Shen, Z. Xin, N. Zhang, Y. Bai, and Y. Mei (2014), Change of soil organic carbon after cropland afforestation in ‘Beijing-Tianjin Sandstorm Source Control’ program area in China, *Chinese Geographical Science*, *24*(4), 461-470.
884. Zeng, X. P. (2007), Net primary productivity, structure and function of three plantation communities in Heshan hilly land [D], *Guangdong: South China Botanical Garden, CAS*. (in Chinese)
885. Zeng, Z., S. Wang, C. Zhang, C. Gong, and H. U. Qing (2013), Carbon storage in evergreen broad-leaf forests in mid-subtropical region of China at four succession stages, *Journal of Forestry Research*, *326*(4), 677-682.
886. Zeng, Z. Q. (2012), Carbon density and seqestration potential of evergreen broad-leaf forests in mid-subtropical region of China at four succession stages [D], *Liaoning: Institute of Applied Ecology, CAS*. (in Chinese)
887. Zeng, Z. X., X. L. Liu, Y. Jia, and F. M. Li (2008), The effect of conversion of cropland to forage legumes on soil quality in a semiarid agroecosystem, *Journal of Sustainable Agriculture*, *32*(2), 335-353.
888. Zha, T. G. (2007), Carbon balance of a poplar plantation ecosystem in Daxing, Beijing [D], *Beijing: Beijing Forestry University*. (in Chinese)
889. Zhai, M. Y., M. Zhou, P. W. Zhao, G. S. Fu, J. H. Liu, and J. W. Zhang (2014), Carbon content and carbon density of Populus plantation in Tongliao City, *Journal of Arid Land Resources and Environment*, *28*(6), 57-62. (in Chinese)
890. Zhan, C., J. Cao, Y. Han, S. Huang, X. Tu, P. Wang, and Z. An (2013), Spatial distributions and sequestrations of organic carbon and black carbon in soils from the Chinese loess plateau, *Science of the Total Environment*, *465*, 255-266.
891. Zhan, Z. Q. (2011), Research on biomass and carbon storage of Moso bamboo in Sheshan area, Shanghai [D], *Shanghai: Shanghai Jiao Tong University*. (in Chinese)
892. Zhang, B., S. Chen, X. He, W. Liu, Q. Zhao, L. Zhao, and C. Tian (2014), Responses of soil microbial communities to experimental warming in alpine grasslands on the Qinghai-Tibet Plateau, *PloS one*, *9*(8), e103859.
893. Zhang, C., G. Liu, S. Xue, and C. Sun (2013), Soil organic carbon and total nitrogen storage as affected by land use in a small watershed of the Loess Plateau, China, *European Journal of Soil Biology*, *54*(1), 16-24.
894. Zhang, D. G., Y. Liu, H., W. Z. Luo, and S. Z. Quan (2010), Nutrient characteristics in paddy soils of Hani terrace in Luchun County of Yunnan Province, *Bulletin of Soil and Water Conservation*, *30*(5), 91-95. (in Chinese)
895. Zhang, D. M. (2012), Study carbon budge of desert shrubs in Alashan desert region [D], *Inner: Inner Mongolia Agricultural University*. (in Chinese)
896. Zhang, F., B. Qi, F. Wen, D. G. Zhang, H. Wu, and L. Zhang (2011), Analysis of the change of carbon storage in alpine arid grassland, *Acta Prataculturae Sinica*, *20*(4), 11-18. (in Chinese)
897. Zhang, G. (2010), Changes of soil labile organic carbon in different land uses in Sanjiang Plain, Heilongjiang Province, *Chinese Geographical Science*, *20*(2), 139-143.
898. Zhang, G. B. (2008), Forest carbon storage dynamics at the upper stream of Minjing river [D], *Beijing: Chinese Academy of Forestry*. (in Chinese)
899. Zhang, G. B., X. Q. Li, Z. H. Xu, C. Q. Hu, S. N. Zhang, and G. H. Hu (2012), Analysis of the carbon stock structure in forest plantations with different regeneration methods, *Ecology and Environmental Sciences*, *21*(2), 206-212. (in Chinese)
900. Zhang, G. Q. (2008), The impact to carbon stocks of artificial Pine forest ecosystems with different management measures [D], *Sichuan: Sichuan Agricultural University*. (in Chinese)
901. Zhang, G. Q., C. D. Huang, H. Guo, B. Deng, and H. F. Yang (2007), Spatial distribution property of carbon stocks in artificial Pine ecosystems with different density, *Journal of Zhejiang Forestry Science and Technology*, *27*(6), 10-14. (in Chinese)
902. Zhang, H. (2010), Study on the dominant tree biomass and carbon storage in Daqing mountain [D], *Inner: Inner Mongolia Agricultural University*. (in Chinese)
903. Zhang, H., D. S. Guan, and M. W. Song (2012), Biomass and carbon storage of Eucalyptus and Acacia plantations in the Pearl River Delta, South China, *Forest Ecology & Management*, *277*(4), 90–97.
904. Zhang, H., T. Q. Song, K. L. Wang, H. G. Zhu, Y. G. Wen, W. X. Peng, H. Du, Q. J. Tan, B. S. Ouyang, and F. P. Zeng (2013), Oak biomass and its allocation at different stand ages in west of Guangxi, China, *Research of Agricultural Modernization*, *34*(6), 758-762. (in Chinese)
905. Zhang, H. Y. (2013), Yangtze estuary typical tidal wetlands - Chongxi wetland soil organic carbon distribution and research of ecological engineering influence [D], *Shanghai: East China Normal University*. (in Chinese)
906. Zhang, J. (2008), Study on community structure, biomass and carbon storage of *Larix gmelini* plantation [D], *Beijing: Beijing Forestry University*. (in Chinese)
907. Zhang, J., F. Li, Y. Wang, and D. Xiong (2013), Soil organic carbon stock and distribution in cultivated land converted to grassland in a subtropical region of China, *Environmental Management*, *53*(2), 274-283.
908. Zhang, J., Y. Li, S. X. Chang, P. Jiang, G. Zhou, J. Liu, J. Wu, and Z. Shen (2014), Understory vegetation management affected greenhouse gas emissions and labile organic carbon pools in an intensively managed Chinese chestnut plantation, *Plant & Soil*, *376*(1-2), 363-375.
909. Zhang, J., S. Wang, Z. Feng, and Q. Wang (2009), Stability of soil organic carbon changes in successive rotations of Chinese fir (*Cunninghamia lanceolata* (*Lamb.*) hook) plantations, *Journal of Environmental Sciences*, *21*(21), 352-359.
910. Zhang, J., X. Wang, and J. Wang (2014), Impact of land use change on profile distributions of soil organic carbon fractions in the Yanqi Basin, *Catena*, *115*, 79-84.
911. Zhang, J., Y. Wang, X. Zhao, G. Xie, and T. Zhang (2005), Grassland recovery by protection from grazing in a semi-arid sandy region of northern China, *New Zealand Journal of Agricultural Research*, *48*(2), 277-284.
912. Zhang, J. B., C. C. Song, and S. M., Wang (2007), Dynamics of soil organic carbon and its fractions after abandonment of cultivated wetlands in northeast China, *Soil and Tillage Research*, *96*(1), 350-360.
913. Zhang, J. K., Q. J. Hao, C. S. Jiang, and Y. Wu (2011), Effect of tillage systems on soil organic carbon and soil quality in a purple paddy soil, *Advanced Materials Research*, *183*, 1190-1194.
914. Zhang, J. Q., Y. Q. Su, Y. X. Kang, X. M. Xu, and Y. Qin (2009), Carbon sequestration of young Robinia pseudoacacia plantation in Loess Plateau, *Chinese Journal of Applied Ecology*, *20*(12), 2911-2916. (in Chinese)
915. Zhang, J. Q., X. M. Xu, X. F. Wang, L. Wang, T. T. Zhang, and X. C. Zhao (2011), Carbon sink in artificial forest ecosystem of Robina and Piuns in Loess Plateau, *Arid Land Geography*, *34*(2), 201-207. (in Chinese)
916. Zhang, K., X. Xu, Q. Wang, and B. Liu (2010), Biomass, and carbon and nitrogen pools in a subtropical evergreen broad-leaved forest in eastern China, *Journal of Forest Research*, *15*(4), 274-282.
917. Zhang, L. (2013), Soil organic carbon in Mangrove forests and its relationship with soil factor in Qinglangang, Hainan [D], *Henan: Henan University of Science and Technology*. (in Chinese)
918. Zhang, L., Z. Xie, R. Zhao, and Y. Wang (2012), The impact of land use change on soil organic carbon and labile organic carbon stocks in the Longzhong region of Loess Plateau, *Journal of Arid Land*, *4*(3), 241-250.
919. Zhang, L. L., Y. Q. Su, Y. Liu, D. F. He, H. X. Gao, J. Wang, and X. M. Xu (2013), Study on organic carbon of Platycladus orientalis plantation in Qianyang loess plateau, *Journal of Central South Forestry University*, *33*(2), 56-60. (in Chinese)
920. Zhang, L. M., T. B. He, M. G. Xu, Y. L. Lou, P. Zhang, and X. L. Wang (2013), Soil organic carbon and nitrogen stocks under conservation tillage in upland southern China, *Soil and Crop*, *2*(3), 112-116. (in Chinese)
921. Zhang, M. (2013), Effects of long term rotation and fertilization on the distribution of organic carbon and nitrogen in soil aggregates [D], *Shanxi: Northwest Agriculture and Forestry University*. (in Chinese)
922. Zhang, M., X. K. Zhang, W. J. Liang, Y. Jiang, G. H. Dai, X. G. Wang, and S. J. Han (2011), Distribution of soil organic carbon fractions along the altitudinal gradient in Changbai Mountain, China, *Pedosphere*, *21*(5), 615-620.
923. Zhang, N. Y., J. Q. Zhang, Y. X. Yang, L. Wang, X. F. Wang, and N. Q. Li (2009), Impact of different artificial ecological forests on soil nutrients in Loess Plateau, *Journal of Northeast Forestry University*, *37*(11), 74-76. (in Chinese)
924. Zhang, P. (2009), Study on forest carbon stock in Beijing of China [D], *Beijing: Beijing Forestry University*. (in Chinese)
925. Zhang, P., T. Zhang, and N. L. Chen (2009), Vertical distribution patterns of soil organic carbon and total nitrogen and related affecting factors along northern slope of Qilian mountains, *Chinese Journal of Applied Ecology*, *20*(3), 518-524. (in Chinese)
926. Zhang, Q. Z., and C. K. Wang (2010), Carbon density and distribution of six Chinese temperate forests, *Science China Life Sciences*, *53*(53), 831-840.
927. Zhang, S., J. Wen, T. Li, X. Xu, L. Deng, G. Gong, and C. Hu (2012), Soil carbon fractions of restored lands in Liusha River Valley, Sichuan, *Ecological Engineering*, *40*(3), 27-36.
928. Zhang, S., M. X. Xu, Y. F. Zhang, C. H. Wang, and G. Chen (2014), Effects of land use change on storage of soil organic carbon in deep soil layers in the hilly Loess Plateau region, China, *Acta Scientiae Circumstantiae*, *34*(12), 3094-3101. (in Chinese)
929. Zhang, S., X. Zhang, Z. Liu, Y. Sun, W. Liu, L. Dai, and S. Fu (2014), Spatial heterogeneity of soil organic matter and soil total nitrogen in a Mollisol watershed of Northeast China, *Environmental Earth Sciences*, *72*(1), 275-288.
930. Zhang, S. J. (2008), The study of biomass and carbon storage in the ecosystem of secondary forests of Pinus massoniana, Acacia confusa along the coast [D], *Fujian: Fujian Agriculture and Forestry University*. (in Chinese)
931. Zhang, S. P. (2008), The analysis of bio-activity and relativity of different type wetlands soil in Chongming Dongtan [D], *Shanghai: Tongji University*. (in Chinese)
932. Zhang, T., Y. Su, J. Cui, Z. Zhang, and X. Chang (2006), A leguminous shrub (Caragana microphylla) in semiarid sandy soils of North China, *Pedosphere*, *16*(3), 319-325.
933. Zhang, T. T. (2012), Biomass and carbon storage of Larix principis-rupprechtii forest plantation [D], *Beijing: Beijing Forestry University*. (in Chinese)
934. Zhang, W. G., W. D. Xie, G. X. Jiang, J. Y. Lai, H. M. Shi, and H. W. Hu (2011), Effects of age structure of Juglans regia young forest in rocky desertification area on its carbon stock, *Journal of Central South Forestry University*, *31*(8), 96-101. (in Chinese)
935. Zhang, W. J., H. K. Liao, J. Long, J. Li, and L. F. Liu (2014), Effects of land use on soil organic carbon and its turnover rate in Karst mountain areas of Guizhou province, *Chinese Journal of Ecology*, *33*(5), 1297-1303. (in Chinese)
936. Zhang, W. J., P. Q. Peng, C. L. Tong, X. L. Wang, and J. S. Wu (2005), Characteristics of distribution and composition of organic carbon in Dongting lake floodplain, *Chinese Journal of Environmental Science*, *26*(3), 56-60. (in Chinese)
937. Zhang, W. J., J. S. Wu, H. A. Xiao, and C. L. Tong (2004), Profile distribution characteristics and accumulation of organic carbon typical wetlands in Sanjiang Plain, *Advances in Earth Sciences*, *19*(4), 558-563. (in Chinese)
938. Zhang, W. J., H. A. Xiao, C. L. Tong, Y. R. Su, W. S. Xiang, D. Y. Huang, J. K. Syers, and J. Wu (2008), Estimating organic carbon storage in temperate wetland profiles in Northeast China, *Geoderma*, *146*(1), 311-316.
939. Zhang, W. M., M. Wu, X. X. Shao, X. S. Jiang, and B. Zhou (2014), Changes in soil organic carbon and its active fractions during different reclamation period on the south coast of Hangzhou bay, *Journal of Soil and Water Conservation*, *28*(2), 226-231. (in Chinese)
940. Zhang, W. M., M. Wu, M. Wang, X. X. Shao, X. S. Jiang, and B. Zhou (2014), Distribution characteristics of organic carbon and its components in soils under different types of vegetation in wetland of Hangzhou Bay, *Acta Pedologica Sinica*, *51*(6), 1351-1360. (in Chinese)
941. Zhang, X., H. Li, J. He, Q. Wang, and M. H. Golabi (2009), Influence of conservation tillage practices on soil properties and crop yields for maize and wheat cultivation in Beijing, China, *Soil Research*, *47*(4), 362-371.
942. Zhang, X., X. Meng, L. Gao, X. Sun, J. Fan, and L. Xu (2010), Potential impacts of climate warming on active soil organic carbon contents along natural altitudinal forest transect of Changbai Mountain, *Acta Ecologica Sinica*, *30*(2), 113-117.
943. Zhang, X., Y. Sheng, J. Li, J. Wu, J. Chen, and Y. Cao (2012), Changes of alpine ecosystem along the ground temperature of permafrost in the source region of Datong River in the Northeastern Qinghai-Tibet Plateau, *Journal of Food, Agriculture & Environment*, *10*(1), 970-976.
944. Zhang, X. H., Z. P. Fan, X. K. Sun, Y. L. Hu, D. H. Zeng, and Q. Wang (2009), Effects of land use change on ecosystem carbon stock in semi-arid region, *Chinese Journal of Ecology*, *28*(12), 2424-2430. (in Chinese)
945. Zhang, X. L., S. L. Shi, G. X. Pan, L. Q. Li, X. H. Zhang, and Z. P. Li (2008), Changes in eco-chemical properties of a Mangrove wetland under Spartina invasion from Zhangjiangkou, Fujian, China, *Advances in Earth Sciences*, *23*(9), 974-981. (in Chinese)
946. Zhang, X. L., S. Wang, X. Wang, G. S. Wen, H. W. Liu, and J. L. Li (2014), Soil organic carbon storage of Diaoluoshan natural reserve in Hainan, *Chinese Journal of Tropical Crops*, *35*(2), 362-368. (in Chinese)
947. Zhang, X. L., Z. H. Zhang, Z. J. Xu, X. J. Hou, and Q. F. Cai (2012), On the relation between carbon storage and reinforced fixation of the coastal wetland vegetation in the Yellow River delta area, *Journal of Safety and Environment*, *12*(6), 145-149. (in Chinese)
948. Zhang, X. N. (2011), Study on soil carbon pool in Ebinur lake wetland natural reserve [D], *Xinjiang: Xinjiang University*. (in Chinese)
949. Zhang, X. R., Z. Q. Xu, X. L. Ji, Y. L. Jia, X. R. Huang, J. P. Lu, and J. S. Zhang (2010), Soil organic carbon storage and its distribution of the typical communities in the north region of Yanshan mountain, *Journal of Soil and Water Conservation*, *24*(1). (in Chinese)
950. Zhang, X. S., W. F. Feng, P. X. Li, G. S. Liu, Y. X. Shan, and Y. H. Zhang (2014), Carbon distribution characteristics of Jigongshan mountain deciduous oak forest ecosystem in the transition region from warm temperate zone to subtropics, *Journal of Xinyang Normal University (Natural Science Edition)*, *27*(3), 363-367. (in Chinese)
951. Zhang, X. S., B. Wang, W. F. Feng, H. T. Dai, G. S. Liu, Y. X. Shan, X. Q. Li, and H. H. Dong (2013), Spatial patterns of soil organic carbon in oak and pine-oak mixed forests in Jigong Mountain in temperate-subtropical ecotone, *Journal of Anhui Agricultural University*, *40*(1), 18-22. (in Chinese)
952. Zhang, X. W. (2013), Study on cropland and soil carbon sequestration rate at county scale of the northwest semi-humid and semi-arid area [D], *Shanxi: Northwest Agriculture and Forestry University*. (in Chinese)
953. Zhang, X. W., and M. X. Xu (2013), Soil organic carbon sequestration rate and its influencing factors in farmland of Guanzhong Plain: a case study in Wugong county, Shanxi province, *Environmental Science*, *34*(7), 2793-2799. (in Chinese)
954. Zhang, X. Y., D. S. Guan, H. S. Li, K. Y. Huang, and Z. C. Xu (2009), Allocation characteristics of organic carbon pool in typical forest soils in Guangzhou, *Acta Scientiarum Naturalium Universitatis Sunyatseni*, *48*(5), 137-142. (in Chinese)
955. Zhang, X. Y., Z. C. Xu, F. T. Zeng, X. B. Hu, and Q. P. Han (2011), Carbon density distribution and storage dynamics of forest ecosystem in Pearl River Delta of low subtropical China, *China Environmental Science*, *31*, 69-77. (in Chinese)
956. Zhang, Y. (2012), Study on the carbon stock of Baxianshan forest with remote sensing technology [D], *Tianjing: Tianjing Normal University*. (in Chinese)
957. Zhang, Y., B. Duan, J. R. Xian, H. Korpelainen, and C. Li (2011), Links between plant diversity, carbon stocks and environmental factors along a successional gradient in a subalpine coniferous forest in Southwest China, *Forest Ecology & Management*, *262*(3), 361–369.
958. Zhang, Y., F. Gu, S. Liu, Y. Liu, and C. Li (2013), Variations of carbon stock with forest types in subalpine region of southwestern China, *Forest Ecology & Management*, *300*(300), 88-95.
959. Zhang, Y. J., S. L. Guo, Y. F. Nan, and Z. Li (2012), The changes and influencing factors of soil C:N ratio in small watershed of hilly region of Loess Plateau, *Journal of Natural Resources*, *27*(7), 1214-1223. (in Chinese)
960. Zhang, Y. N., Y. L. Li, L. Wang, J. H. Chen, Y. Hu, X. H. Fu, and Y. Q. Le (2012), Variability in organic carbon storage capability of soils at different successional stages in Chongming Dongtan wetland and its microbial mechanism, *Journal of Agro-Environment Science*, *31*(3), 631-637. (in Chinese)
961. Zhang, Y. T., S. S. Hu, J. M. Li, J. J. Lu, and W. D. Wang (2013), Characteristic of root biomass of three main forest types in Xinjiang, *Arid Land Geography*, *36*(2), 269-276. (in Chinese)
962. Zhang, Y. W. (2009), Plant Flora and physicochemical properties of soil of peat swamp in Xiaoxing' an mountains [D], *Heilongjiang: Northeast Forestry University*. (in Chinese)
963. Zhang, Z., Q. L. Zhong, D. L. Cheng, C. B. Xu, B. Hu, and Y. N. Chang (2014), The structure characteristics of carbon storage of ecosystem of ever-green broad-leaved mixed forest with different forest ages in the north-west of Fujian province, *Ecology and Environmnet*, *23*(2), 203-210. (in Chinese)
964. Zhang, Z. H., L. C. Wang, D. R. Zheng, and J. X. Luo (2011), Study on artificial forest stand biomass of Pinus yunnanensis faranch in northwest Yunnan province, *Journal of Anhui Agricultural Sciences*, *39*(31), 19203-19205. (in Chinese)
965. Zhang, Z. J., X. Q. Zhang, Y. H. Wang, Y. J. Luo, Z. Y. Li, and L. Cao (2009), Carbon storage and distribution of Pinus massoniana forest ecosystem in Tiesbanping of Chongqing, *Scientia Silvae Sinicae*, *45*(5), 49-53. (in Chinese)
966. Zhang, Z. M., S. X. Lin, Q. H. Zhang, Y. Guo, and C. H. Lin (2013), The distribution characteristics of soil carbon, nitrogen and phosphorus under different land use patterns in Caohai Plateau wetland, *Journal of Soil and Water Conservation*, *27*(6), 199-204. (in Chinese)
967. Zhao, B. B., K. C. Niu, and G. Z. Du (2009), The effect of grazing on above-ground biomass allocation of 27 plant species in an alpine meadow plant community in Qinghai-Tibetan Plateau, *Acta Ecologica Sinica*, *29*(3), 1596-1606. (in Chinese)
968. Zhao, C. D., G. D. Liu, K. Yang, H. F. Nie, M. Peng, T. Li, F. Liu, and Y. S. Li (2011), Estimation of soil carbon storage and its change since 1986 in Zhalong wetland and its surrounding areas, Heilongjiang Province, *Earth Science Frontiers*, *18*(6), 27-33. (in Chinese)
969. Zhao, C. Z., and H. Ren (2012), Individual spatial pattern and spatial association of Stipa krylovii population in Alpine Degraded Grassland, *Acta Ecologica Sinica*, *32*(22), 6946-6954. (in Chinese)
970. Zhao, G. S., F. D. Li, Y. S. Li, Y. Zhang, Z. Y. Ouyang, and Z. R. Tian (2012), Effects of long term fertilization on soil organic carbon matter accumulation, *Ecology and Environmental Sciences*, *21*(5), 840-847. (in Chinese)
971. Zhao, H., W. L. Liu, X. D. Wang, Y. J. Cai, and Z. Y. Du (2014), Distribution of soil carbon and nitrogen under different water conditionsin alpine salty wetlands, northern Tibet Plateau, *Journal of Mountain Research*, *32*(4), 431-437. (in Chinese)
972. Zhao, H., R. Zhou, and S. Drake (2007), Effects of aeolian deposition on soil properties and crop growth in sandy soils of northern China, *Geoderma*, *142*(142), 342-348.
973. Zhao, H. C. (2013), Study on the influence mechanism of agricultural measures on soil organic carbon in spring corn fields [D], *Inner: Inner Mongolia Agricultural University*. (in Chinese)
974. Zhao, H. L., Y. Z. Su, H. Zhang, L. Y. Zhao, and R. L. Zhou (2007), Multiple effects of shrub on soil properties and understory vegetation in Horqin sand land, Inner, Mongolia, *Journal of Desert Research*, *27*(3), 385-390. (in Chinese)
975. Zhao, H. T. (2007), Controls of carbon stocks and carbon mineralization potential in Inner Mongolia grassland [D], *Beijing: Institute of Botany, CAS*. (in Chinese)
976. Zhao, J. (2011), The spatial variability of the soil organic carbon and total nitrogen content in a broad-leaved Korean pine mixed forest in the Changbai Mountains [D], *Liaoning: Institute of Applied Ecology, CAS*. (in Chinese)
977. Zhao, J. M. (2006), Study on the soil organic carbon stock of alpine grassland under different degraded degrees in Eastern Qilian Mountains [D], *Gansu: Gansu Agricultural University*. (in Chinese)
978. Zhao, K. (2010), Study on the carbon storage in pure and mixture plantations Fokienia Hodginsi and Michelia Macclurei [D], *Fujian: Fujian Agriculture and Forestry University*. (in Chinese)
979. Zhao, L. (2013), Analysis of the spatial structure and seasonal variations of the isolated wetland soil carbon, nitrogen and phosphorus in Sanjiang Plain [D], *Jilin: Jilin Normal University*. (in Chinese)
980. Zhao, M., W. Xiang, C. Peng, and D. Tian (2009), Simulating age-related changes in carbon storage and allocation in a Chinese fir plantation growing in southern China using the 3-PG model, *Forest Ecology & Management*, *257*(6), 1520-1531.
981. Zhao, M. J., Y. M. Chen, Z. M. Ai, Y. Cao, and J. H. Xu (2013), Distribution characteristics of soil organic carbon and their affecting factors of typical plantations in Loess Hilly Region, *Bulletin of Soil and Water Conservation*, *33*(2), 270-275. (in Chinese)
982. Zhao, M. Q., and Y. F. Shi (2014), Carbon storage and distribution in Mango plantation ecosystems in Sanya, *Journal of Anhui Agricultural Sciences*, *42*(4), 1088-1090. (in Chinese)
983. Zhao, M. Y., W. W. Zhao, and L. N. Zhong (2014), Scale effect analysis of the influence of land use and environmental factors on surface soil organic carbon: a case study in the hilly and gully area of northern Shaanxi province, *Acta Ecologica Sinica*, *34*(5), 1105-1113. (in Chinese)
984. Zhao, N., P. Meng, J. S. Zhang, S. Lu, and Z. Q. Cheng (2014), Comparison of soil respiration under various land uses in hilly area of northern China, *Scientia Silvae Sinicae*, *50*(2), 1-7. (in Chinese)
985. Zhao, N., Y. Zhuang, and J. Zhao (2014), Effects of grassland managements on soil organic carbon and microbial biomass carbon, *Pratacultural Science*, *31*(3), 367-374. (in Chinese)
986. Zhao, R. F., L. H. Zhang, H. L. Zhao, P. H. Jiang, and J. Z. Wang (2013), Distribution of soil organic carbon of wetlands in the middle reaches of the Heihe river and its influencing factors, *Scientia Geographica Sinica*, *33*(3), 363-370. (in Chinese)
987. Zhao, W., S. Chen, X. Han, and G. Lin (2009), Effects of long-term grazing on the morphological and functional traits of Leymus chinensis in the semiarid grassland of Inner Mongolia, China, *Ecological Research*, *24*(1), 99-108.
988. Zhao, X., P. Wu, X. Gao, L. Tian, and H. Li (2014), Changes of soil hydraulic properties under early-stage natural vegetation recovering on the Loess Plateau of China, *Catena*, *113*, 386-391.
989. Zhao, X. C., L. M. Lai, L. H. Zhu, J. J. Wang, Y. J. Wang, J. H. Zhou, L. H. Jiang, H. B. Lu, C. Q. Zhao, and Y. R. Zheng (2014), Correlation between characteristics of Reaumuria soongarica communities and soil factors in the Sangong River basin, *Acta Ecologica Sinica*, *34*(4), 878-889. (in Chinese)
990. Zhao, Y., W. Fan, Z. M. Wu, J. Zhang, and Y. F. Zhu (2009), The nutrient allocation and cycling pattern in the Platycladus orientalis plantation in Hilly region of Taihang mountains, *Journal of Soil and Water Conservation*, *23*(2), 143-147. (in Chinese)
991. Zhao, Y., M. Z. Wu, W. Fan, Y. F. Zhu, and D. Zhao (2009), The Variation of carbon storage during the community succession processing in hilly region of Taihang mountain, *Journal of Soil and Water Conservation*, *23*(4), 208-212. (in Chinese)
992. Zhao, Z. Y., R. H. Wang, H. Z. Zhang, and L. Wang (2006), Aboveground biomass of Tamarix on piedmont plain of Tianshan mountains south slope, *Chinese Journal of Applied Ecology*, *17*(9), 1557-1562. (in Chinese)
993. Zheng, H. (2012), The estimation of soil organic carbon density of the typical discontinuous karst soil, southwest China [D], *Hunan: Institute of Subtropical Agriculture, CAS*. (in Chinese)
994. Zheng, H., Z. Ouyang, W. Xu, X. Wang, H. Miao, X. Li, and Y. Tian (2008), Variation of carbon storage by different reforestation types in the hilly red soil region of southern China, *Forest Ecology & Management*, *255*(3–4), 1113-1121.
995. Zheng, H., Y. Su, X. He, L. Hu, J. Wu, D. Huang, L. Li, and C. Zhao (2012), Modified method for estimating the organic carbon density of discontinuous soils in peak-karst regions in southwest China, *Environmental Earth Sciences*, *67*(6), 1743-1755.
996. Zheng, X. X., J. M. Zhao, Y. G. Zhang, Y. Q. Wu, T. T. Jin, and G. H. Liu (2007), Variation of grassland biomass and its relationships with environmental factors in Hulunbeier, Inner Mongolia, *Chinese Journal of Ecology*, *26*(4), 533-538. (in Chinese)
997. Zheng, Z., H. M. Liu, and Z. L. Feng (2006), Biomass of tropical montane rain forest in Xishuangbanna of southwest China, *Chinese Journal of Ecology*, *25*(4), 347-353. (in Chinese)
998. Zheng, Z., Y. F. Yang, L. Z. Kong, S. Liu, W. J. Liu, H. J. Si, and P. J. Zhang (2014), Changes of soil physical and chemical properties and microbial biomass in wetland returning farmland to lake in the Caizi Lake, Anhui province, *Resources and Environment in the Yangtza Basin*, *23*(6), 821-826. (in Chinese)
999. Zhong, C. Q. (2009), Impact of land use changes on the soil organic carbon of the Min river estuarine wetlands [D], *Fujian: Fujian Normal University*. (in Chinese)
1000. Zhong, C. Q., J. X. Wang, W. Xing, and W. K. Zhang (2010), Effects of vegetation and hydrological conditions on the profile characteristics of TN, TP and OM in coastal salt marshes in northern Jiangsu Province, *Journal of Beijing Forestry University*, *32*(3), 186-190. (in Chinese)
1001. Zhong, Q. C. (2013), The effects of temperature and water table on the carbon processes in coastal reclaimed wetland: a case study at Dongtan of Chongming island [D], *Shanghai: East China Normal University*. (in Chinese)
1002. Zhong, Q. C., Y. Z. Guan, Q. Liu, L. F. Cao, Y. Lu, L. Wang, and K. Y. Wang (2013), Effects of water table manipulation on the soil respiration in a reclaimed tidal wetland at Dongtan of Chongming Island, China, *Chinese Journal of Applied Ecology*, *24*(8), 2141-2150. (in Chinese)
1003. Zhong, X. F. (2007), Effects of successive monoculture on carbon storage and soil labile carbon in Chinese fir plantations [D], *Fujian: Fujian Normal University*. (in Chinese)
1004. Zhong, Y. X., Y. C. Zhou, and Z. J. Li (2014), Research on the carbon storage and potential carbon sequestration of vegetation in the Trough Valley of a karst area, Yinjiang, *Earth and Environment*, *42*(1), 82-89. (in Chinese)
1005. Zhou, C. F., H. Zhao, Z. Y. Sun, L. X. Zhou, C. Fang, Y. Xiao, Z. F. Deng, Y. B. Zhi, Y. Q. Zhao, and S. Q. An (2014), The invasion of Spartina alterniflora alters carbon dynamics in China’s Yancheng natural reserve, *CLEAN Soil, Air, Water, 43*(2), 159-165.
1006. Zhou, G., C. Zhou, S. Liu, X. Tang, X. Ouyang, D. Zhang, S. Liu, J. Liu, J. Yan, and C. Zhou (2006), Belowground carbon balance and carbon accumulation rate in the successional series of monsoon evergreen broad-leaved forest, *Science in China*, *49*(3), 311-321.
1007. Zhou, G., S. Zhuang, P. Jiang, Q. Xu, H. Qin, M. Wong, and Z. Cao (2011), Soil organic carbon accumulation in intensively managed *Phyllostachys praecox* stands, *The Botanical Review*, *77*(3), 296-303.
1008. Zhou, G. M., J. M. Xu, and P. K. Jiang (2006), Effect of management practices on seasonal dynamics of organic carbon in soils under bamboo plantations, *Pedosphere*, *16*(4), 525-531.
1009. Zhou, H., Y. Chen, and W. Li (2010), Soil properties and their spatial pattern in an oasis on the lower reaches of the Tarim River, northwest China, *Agricultural Water Management*, *97*(11), 1915-1922.
1010. Zhou, M. H. (2008), The study on distribution characteristics of the natural grassland vegetation roots in Yunwu mountain in Ningxia [D], *Shanxi: Northwest Sci-tech University of Agriculture and Forestry*. (in Chinese)
1011. Zhou, P., W. Z. Zhu, J. Luo, Y. C. Chen, Y. Yang, and J. Xie (2013), Aboveground biomass and carbon storage of typical forest types in Gongga mountain, *Acta Botanica Boreali-Occidentalia Sinica*, *33*(1), 162-168. (in Chinese)
1012. Zhou, Q. Q. (2012), Study on the biomass and carbon storage of Pinus massionnina forest in Niumulin natural reserve, Yongchun [D], *Fujian: Fujian Agriculture and Forestry University*. (in Chinese)
1013. Zhou, W. C., C. C. Mu, X. Liu, and H. Gu (2012), Effects of fire disturbance on litter mass and soil carbon storage of Betula platyphylla and Larix gmelinii-Carex schmidtii swamps in the Xiaoxing' an mountains of northeast China, *Acta Ecologica Sinica*, *32*(20), 6387-6395. (in Chinese)
1014. Zhou, W. H., R. Z. Feng, and Y. R. Man (2008), Characteristics of soil in different degraded pasture in the headwaters of the yellow rivers, *Grassland and Turf (Bimonthly), 4*, 24-28. (in Chinese)
1015. Zhou, Y., Z. Pei, J. Su, J. Zhang, Y. Zheng, J. Ni, C. Xiao, and R. Wang (2012), Comparing soil organic carbon dynamics in perennial grasses and shrubs in a saline-alkaline arid region, northwestern China, *PloS one*, *7*(8), e42927.
1016. Zhou, Y., J. Su, I. A. Janssens, G. Zhou, and C. Xiao (2014), Fine root and litterfall dynamics of three Korean pine (*Pinus koraiensis*) forests along an altitudinal gradient, *Plant & Soil*, *374*(1-2), 19-32.
1017. Zhou, Y., L. H. Wang, G. S. Zhang, Y. J. Feng, D. B. Gao, Z. C. Gao, M. R. Hasi, and L. Bian (2013), Distribution characteristics of biomass and carbon of aboveground modules of four shrubs in Mu Us sandy land, *Guangdong Agricultural Sciences*, *1*, 154-157. (in Chinese)
1018. Zhou, Y. J. (2012), A study on soil carbon sequestration performance and fate for high yield soil of the corn belt of the Songliao Plain [D], *Jilin: Jilin Agricultural University*. (in Chinese)
1019. Zhou, Y. T., G. Fu, Z. X. Shen, X. Z. Zhang, J. S. Wu, Y. L. Li, and P. W. Yang (2013), Estimation model of aboveground biomass in the Northern Tibet Plateau based on remote sensing date, *Acta Prataculturae Sinica*, *22*(1), 120-129. (in Chinese)
1020. Zhou, Z. C., Z. T. Gan, Z. P. Shanguan, F. P. Zhang (2013), Effects of long-term repeated mineral and organic fertilizer applications on soil organic carbon and total nitrogen in a semi-arid cropland, *European Journal of Agronomy*, *45*, 20-26.
1021. Zhou, Z. Y. (2006), Effects of land-use change on grassland ecosytems in an agro-pastoral ecotone of Inner Mongolia [D], *Beijing: Institute of Botany, CAS*. (in Chinese)
1022. Zhu, B., X. Wang, J. Fang, S. Piao, H. Shen, S. Zhao, and C. Peng (2010), Altitudinal changes in carbon storage of temperate forests on Mt Changbai, Northeast China, *Journal of Plant Research*, *123*(4), 439-452.
1023. Zhu, J. X., Q. F. Wang, N. P. He, R. M. Wang, and J. Z. Dai (2013), Soil nitrogen mineralization and associated temperature sensitivity of different Inner Mongolian grasslands, *Acta Ecologica Sinica*, *33*(19), 6320-6327. (in Chinese)
1024. Zhu, J. Y., L. J. Mo, Y. C. Ye, Z. Y. Su, H. R. Lv, G. Liu, and S. S. Liu (2011), Study on carbon storage of the forest ecosystem in Dongguan, *Forestry Science and Technology of Guangdong Province*, *27*(2), 22-29. (in Chinese)
1025. Zhu, L. Y., J. J. Pan, and W. Zhang (2013), Study on soil organic carbon pools and turnover characteristics along an elevation gradient in Qilian mountain, *Chinese Journal of Environmental Science*, *34*(2), 668-675. (in Chinese)
1026. Zhu, Q. G. (2007), Research on soil respiration dynamic and poplar fine root for hybird polar (Populus ×euramericana cv.) plantation patterns in the northern areas of Jiangsu province [D], *Jiangsu: Nanjing Forestry University*. (in Chinese)
1027. Zhu, S. Y., K. L. Wang, F. P. Zeng, S. X. Zeng, and T. Q. Song (2009), Properties of soil organic layer of three shrub communities in karst area,Northwest Guangxi, China, *Chinese Journal of Applied and Environmental Biology*, *15*(4), 448-452. (in Chinese)
1028. Zhu, T. H., S. L. Cheng, H. J. Fang, G. R. Yu, J. J. Zheng, and Y. N. Li (2011), Early responses of soil CO2 emission to simulating atmospheric nitrogen deposition in an alpine meadow on the Qinghai-Tibetan Plateau, *Acta Ecologica Sinica*, *31*(10), 2687-2696. (in Chinese)
1029. Zhu, X. Y., and Y. C. Hu (2014), Characteristics of soil nutrient and enzyme activities of the wetland in old yellow river of eastern Henan, *Hubei Agricultural Sciences*, *53*(10), 2268-2272. (in Chinese)
1030. Zhu, Y. F. (2013), Researches on aboveground carbon storage and energy of north subtropical Quercus acutissima plantations [D], *Jiangsu: Nanjing Forestry University*. (in Chinese)
1031. Zhu, Y. F., H. B. Hu, N. Xu, and S. H. Zhang (2014), The distributing characteristics of aboveground biomass and carbon storage of Quercus acutissima plantation with different ages, *China Forestry Science and Technology*, *28*(1), 20-24. (in Chinese)
1032. Zhuang, H. L. (2012), Study on the carbon dynamic of Metasequoia glyptostroboides plantation Ecosystems in Chongming island, Shanghai [D], *Shanghai: Shanghai Jiao Tong University*. (in Chinese)
1033. Zhuang, Q. L. (2007), Distribution of carbon, nitrogen and phosphorus in farmland soil of Songliao Plain [D], *Liaoning: Institute of Applied Ecology, CAS*. (in Chinese)
1034. Zou, C., K. Wang, T. Wang, and W. Xu (2007), Overgrazing and soil carbon dynamics in eastern Inner Mongolia of China, *Ecological Research*, *22*(1), 135-142.
1035. Zou, J. L. (2012), Watershed scale variation of soil organic carbon and soil water during vegetation restoration in Loess Plateau [D], *Shanxi: Northwest Agriculture and Forestry University*. (in Chinese)
1036. Zuo, X., H. Zhao, X. Zhao, Y. Guo, J. Yun, S. Wang, and T. Miyasaka (2009), Vegetation pattern variation, soil degradation and their relationship along a grassland desertification gradient in Horqin Sandy Land, northern China, *Environmental Geology*, *58*(6), 1227-1237.

**Part 2 Unpublished data**

Hu Zhongmin^1, 2^ supply above-ground and below-ground biomass of forest in 149 sampling sites and SOC of forest in 101 sampling sites

(E-mail: [huzm@igsnrr.ac.cn](mailto:huzm@igsnrr.ac.cn) (Z. H.))

Wang Qiufeng^1, 2^ supply above-ground and below-ground biomass of forest and shrub in 98 sampling sites

(E-mail: [qfwang@igsnrr.ac.cn](mailto:qfwang@igsnrr.ac.cn) (Q. W.))

Chen Quansheng^3^ supply above-ground biomass and SOC of grassland in 35 sampling sites

(E-mail: [cqs@ns.ibcas.ac.cn](mailto:cqs@ns.ibcas.ac.cn) (Q. C.))

Huang Mei^1, 2^ supply above-ground, below-ground biomass and SOC of grassland in 36 sampling sites

(E-mail: [huangm@igsnrr.ac.cn](mailto:huangm@igsnrr.ac.cn) (M. H.))

Hu Zhongmin^1, 2^ supply above-ground and below-ground biomass of grassland in 360 sampling sites

(E-mail: [huzm@igsnrr.ac.cn](mailto:huzm@igsnrr.ac.cn) (Z. H.))

Wang Changhui^4^ supply above-ground, below-ground biomass and SOC of grassland in 70 sampling sites

(E-mail: [wangch@ibcas.ac.cn](mailto:wangch@ibcas.ac.cn) (C. W.))

Li Jie^5^ supply above-ground and below-ground biomass of grassland in 13 sampling sites

(E-mail: [lij625@nenu.edu.cn](mailto:lij625@nenu.edu.cn) (J. L.))

Xue Jingyue^6^ supply above-ground biomass of grassland in 35 sampling sites

(E-mail: [xuejy@cib.ac.cn](mailto:xuejy@cib.ac.cn) (J. X.))

He Nianpeng^1, 2^ supply above-ground biomass of grassland in 342 sampling sites, below-ground biomass of grassland in 68 sampling sites, and SOC of grassland in 8 sampling sites

(E-mail: [henp@igsnrr.ac.cn](mailto:henp@igsnrr.ac.cn) (N. H.))

^1^ Key Laboratory of Ecosystem Network Observation and Modeling, Institute of Geographic Sciences and Natural Resources Research, Chinese Academy of Sciences, Beijing 100101, China

^2^ College of Resources and Environment, University of Chinese Academy of Sciences, Beijing 100049, China

^3^ Laboratory of Quantitative Vegetation Ecology, Institute of Botany, Chinese Academy of Sciences, Beijing 100093, China

^4^ State Key Laboratory of Vegetation and Environmental Change, Institute of Botany, Chinese Academy of Sciences, Beijing 100093, China

^5^ Key Laboratory of Vegetation Ecology, Institute of Grassland Science, Northeast Normal University, Ministry of Education, Changchun 130024, China

^6^ Chengdu Institute of Biology, Chinese Academy of Sciences, Chengdu 610041, China

**Supplementary Appx.S10**

Appx.S10 Key code we used to analyze the dataset

(1) Data preparation

According to the C density estimation methods (section 2.2), we estimated the AGBC, BGBC, Veg_C (AGBC + BGBC), SOC, and the ecosystem C (Veg_C + SOC) density of the 18 ecological regions. Meanwhile, we prepared data of the climate (MAT and MAP), soil nutrient (Soil_N, Soil_P, and Soil_K), and soil texture (Soil_Clay, Soil_Silt, and Soil_Sand) of the 18 ecological regions. And the third part of section 2.1 introduced the data sources and processing. We stored this data in “csv” format, and named it “data1”. Then, this file was stored in computer E disk.

(2) Data analysis

① We used general linear model (GLM) to assess variation in AGBC, BGBC, Veg_C (AGBC + BGBC), SOC (0–20 cm and 0–100 cm soil layers), and the ecosystem (Veg-C + SOC) explained by climate, soil nutrient, and soil texture. For Veg_C storage, analyzed factors included climate (MAT and MAP), soil nutrient (soil N, P, and K), and soil texture (clay, silt, and sand); for SOC storage, analyzed factors included SOM input (Veg-C), climate (MAT and MAP) and soil properties (clay, silt, and sand, soil N, P, and K).The GLM analysis was conducted using the lm function in the R package (R project 3.1.2, R development team, 2014).

1) For AGBC, the codes were as follows:

setwd("E:/R")

l<-read.table('E:/R/data1.csv',header=TRUE,sep=',')

l

m<-lm(AGCD~MAP+MAT+Soil_Clay+Soil_Silt+Soil_Sand+Soil_N+Soil_P+Soil_K,data=l)

anova(m)

2) For BGBC, the codes were as follows:

setwd("E:/R")

l<-read.table('E:/R/data1.csv',header=TRUE,sep=',')

l

m<-lm(BGCD~MAP+MAT+Soil_Clay+Soil_Silt+Soil_Sand+Soil_N+Soil_P+Soil_K,data=l)

anova(m)

3) For Veg_C (AGBC+BGBC), the codes were as follows:

setwd("E:/R")

l<-read.table('E:/R/data1.csv',header=TRUE,sep=',')

l

m<-lm(Veg_C~MAP+MAT+Soil_Clay+Soil_Silt+Soil_Sand+Soil_N+Soil_P+Soil_K,data=l)

anova(m)

4) For SOC020 (0–20 cm), the codes were as follows:

setwd("E:/R")

l<-read.table('E:/R/data1.csv',header=TRUE,sep=',')

l

m<-lm(SOC020~Veg_C+MAP+MAT+Soil_Clay+Soil_Silt+Soil_Sand+Soil_N+Soil_P+Soil_K,data=l)

anova(m)

5) For SOC0100 (0–100 cm), the codes were as follows:

setwd("E:/R")

l<-read.table('E:/R/data1.csv',header=TRUE,sep=',')

l

m<-lm(SOC0100~Veg_C+MAP+MAT+Soil_Clay+Soil_Silt+Soil_Sand+Soil_N+Soil_P+Soil_K,data=l)

anova(m)

6) For Ecosystem_C (Veg_C+ SOC0100), the codes were as follows:

setwd("E:/R")

l<-read.table('E:/R/data1.csv',header=TRUE,sep=',')

l

m<-lm(Ecosystem_C~MAP+MAT+Soil_Clay+Soil_Silt+Soil_Sand+Soil_N+Soil_P+Soil_K,data=l)

anova(m)

② Then，path analysis was used to quantitatively investigate the main factors influencing the spatial patterns of Veg-C and SOC (0–20 cm soil layer). Because some of the predictors are correlated, we used path analysis to determine significant direct predictors of the Veg_C and SOC, as well as indirect pathways. For Veg_C storage, analyzed factors included climate (MAT and MAP), soil nutrient (soil N, P, and K), and soil texture (clay, silt, and sand); for SOC storage, analyzed factors included SOM input (Veg-C), climate (MAT and MAP) and soil properties (clay, silt, and sand, soil N, P, and K). The initial path analysis models for Veg_C and SOC density were fully identified, including all possible causal links between observed predictors (e.g., MAT and MAP) and response variable (e.g., soil N, K), and all correlations among predictors. These models provided estimates and significance tests for all potential paths among variables. We trimmed the initial models by retaining significant direct predictor variables. Significance and goodness of fit of the trimmed models were assessed with following indices: a *χ*^2^ test, Bentler’s comparative fit index (CFI) (>0.95), and the standardized root mean residual (<0.08). We built path diagrams using standardized path coefficients between the predictors and response variable, and correlation coefficients between predictors. The analysis was performed by SPSS software (version 18.0, Chicago, IL, USA).
